# Supplementary figures and images for: Complex evolution in Aphis gossypii group (Hemiptera: Aphididae), evidence of primary host shift and hybridization between sympatric species
Source: PLoS One. 2021 Feb 4;16(2):e0245604. doi: 10.1371/journal.pone.0245604 (PMC7861460; doi:10.1371/journal.pone.0245604)

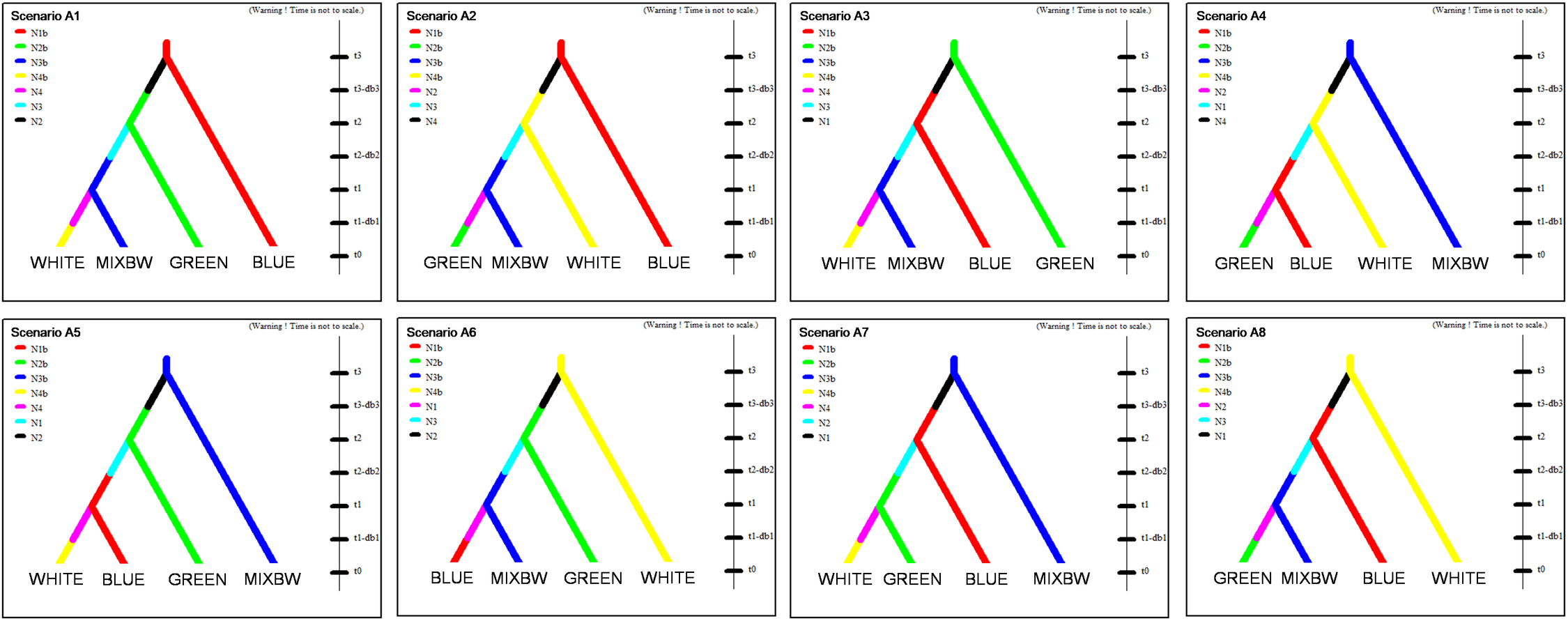

Supplement: S1 Fig — The first eight scenarios (A1–A8) for the DIYABC analyses to infer the host evolution of the two Aphis species, using a dataset that includes 578 individuals from four population groups, which consisted of 75 individuals from the ‘BLUE’ group (Ar_SE, Ar_PE, An_IX, An_YO, Ar_CO, Ar_PH, Ar_RH, Ar_LE); 90 from the ‘GREEN’ group (Ar_ST, Ar_VE, Ar_LY, Ar_CB, Ar_RU); 30 from the ‘MIXBW (BLUE+WHITE)’ group (Ag_RH, Ag_CJ); and 361 from the ‘WHITE’ group (Ag-IL, Ag_CE, Ag_EU, Ag_EJ, Ag_PU, Ag_CU, Ag_CM, Ag_KA, Ag_EL, Ag_HI, Ag_HR, Ag_FO, Ag_CI, Ag_ER, Ag_SN, Ag_CO, Ag_SO, Ag_CA, Ag_CP, Ag_CL, Ag_CT). (TIF) [file pone.0245604.s001.tif]

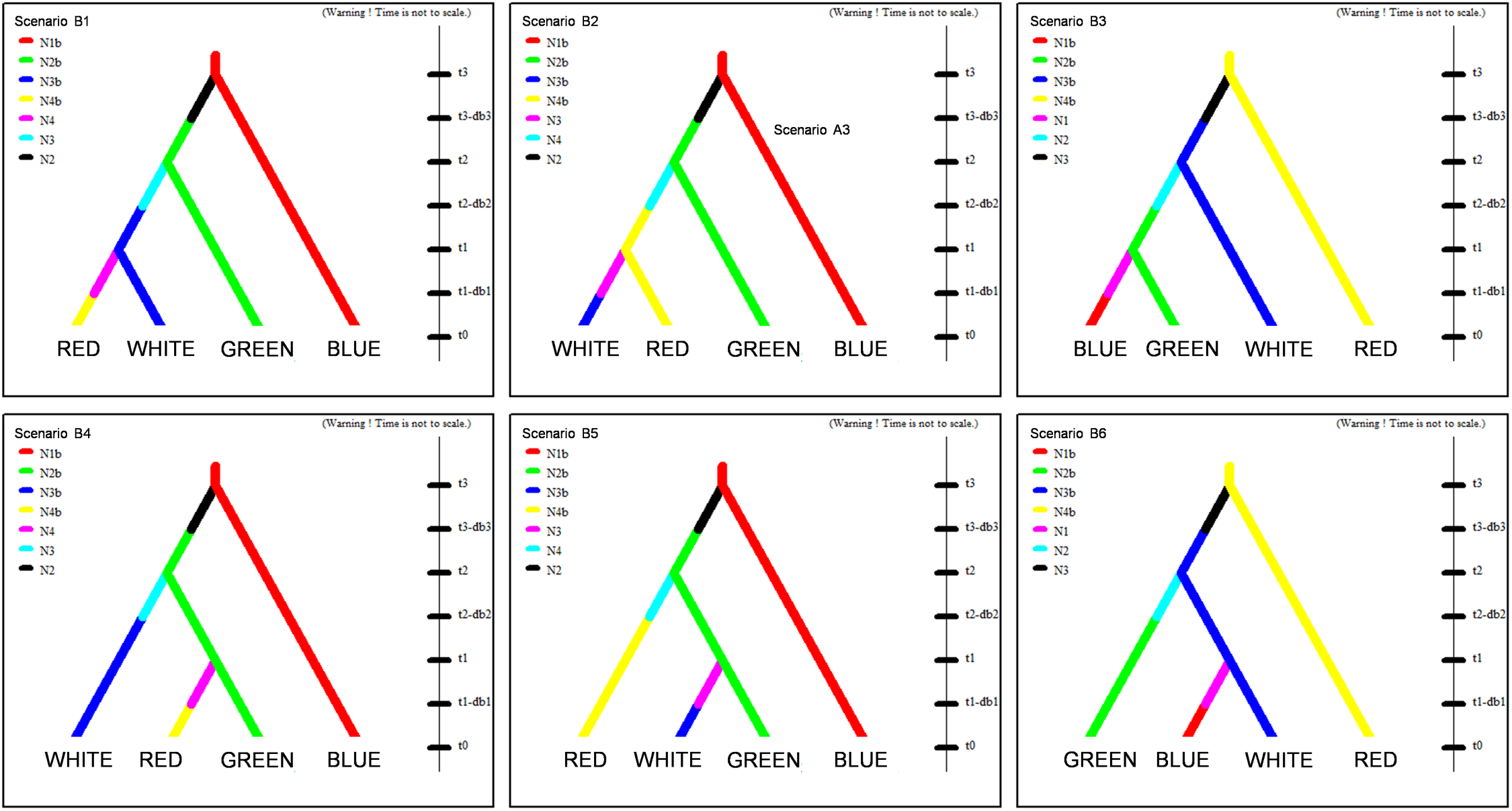

Supplement: S2 Fig — The second six scenarios (B1–B6) for the DIYABC analyses to infer the host evolution of the two Aphis species, using a dataset that includes 311 individuals from four population groups, which consisted of 75 individuals from the ‘BLUE’ group (Ar_CO, Ar_PH, Ar_RH, Ar_SE, Ar_PE, Ar_LE); 90 from the ‘GREEN’ group (Ar_ST, Ar_VE, Ar_LY, Ar_CB, Ar_RU); 60 from the ‘RED’ group (Ag_IL, Ag_CU, Ag_CA, Ag_CP); and 86 from the ‘WHITE’ group (Ag_CE, Ag_FO, Ag_ER, Ag_SN, Ag_CO, Ag_CL, Ag_CT). (TIF) [file pone.0245604.s002.tif]

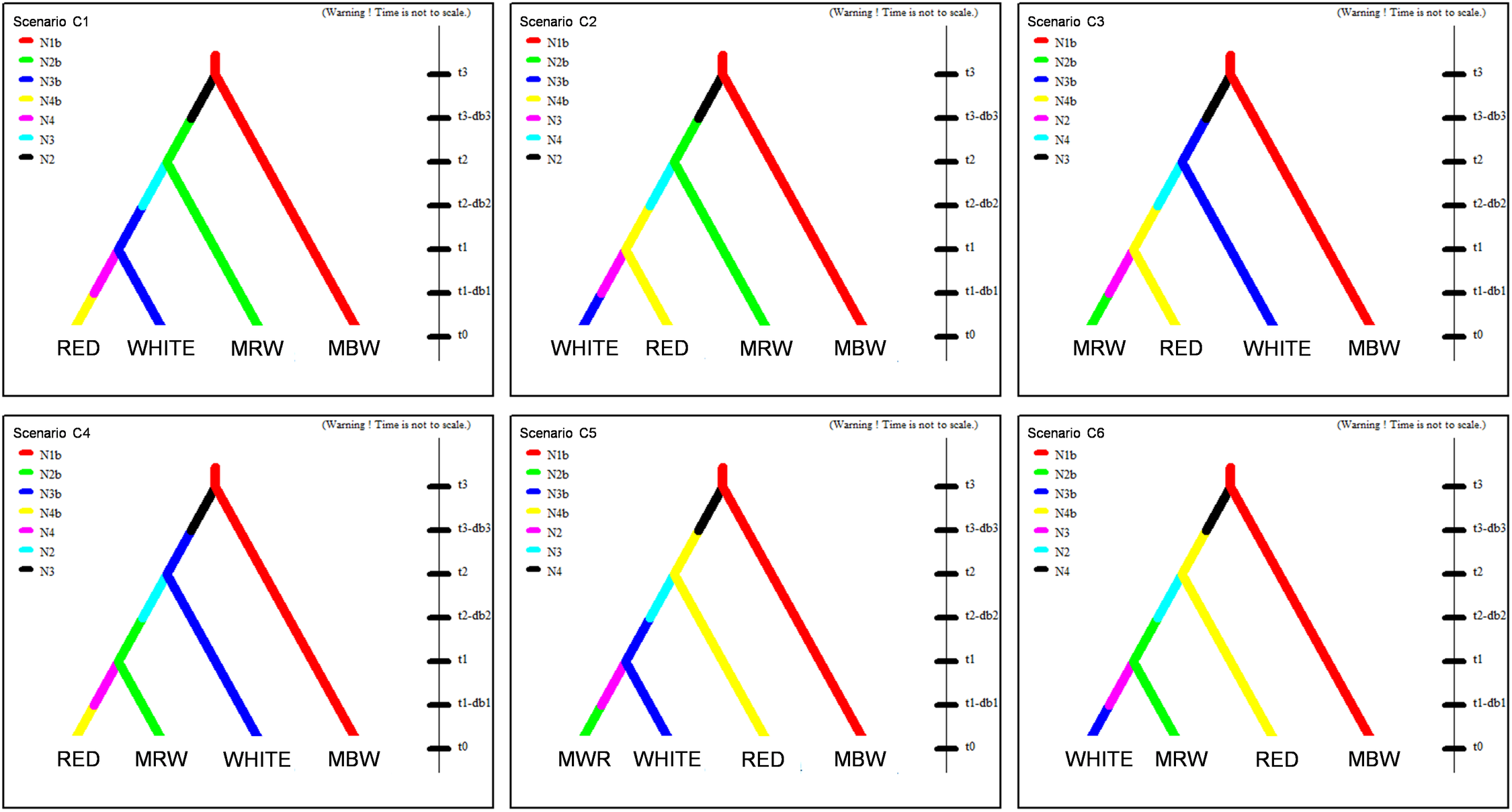

Supplement: S3 Fig — The third six scenarios (C1–C6) for the DIYABC analyses to infer the host evolution of Aphis gossypii, using a dataset that includes 391 individuals from four population groups except for BLUE and GREEN groups in the first and second analysis, which consisted of 30 individuals from the ‘MBW (BLUE+WHITE)’ group (Ag_RH, Ag_CJ); 207 from the ‘MRW (RED+WHITE)’ group (Ag_EU, Ag_EJ, Ag_PU, Ag_SO, Ag_CM, Ag_EL, Ag_HI, Ag_HR, Ag_CI); 68 from the ‘RED’ group (Ag-IL, Ag_CU, Ag_KA, Ag_CA, Ag_CP); and 86 from the ‘WHITE’ group (Ag_CE, Ag_FO, Ag_ER, Ag_SN, Ag_CO, Ag_CL, Ag_CT). (TIF) [file pone.0245604.s003.tif]

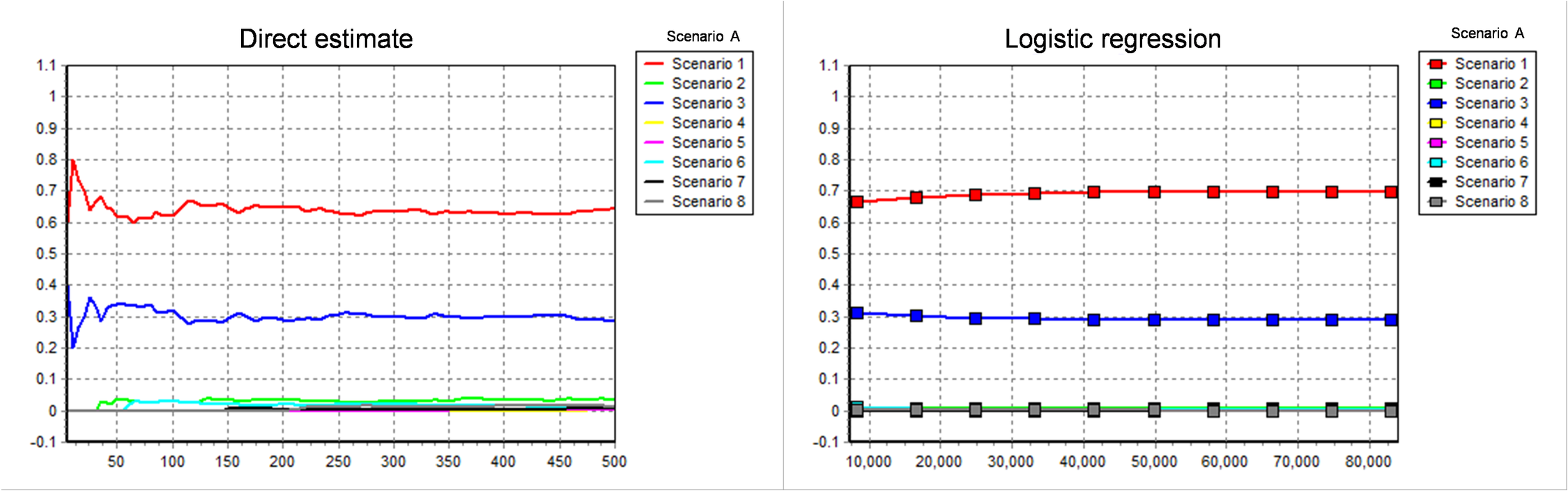

Supplement: S4 Fig — Plots output by DIYABC showing the PP (y-axis) of the first eight scenarios (A1–A8) through the direct estimate (left), and the logistic regression (right) approaches, as output by DIYABC. The x-axis corresponds to the different nδ values used in the computations. The results have been obtained by performing the first analysis with four scenarios. (TIF) [file pone.0245604.s004.tif]

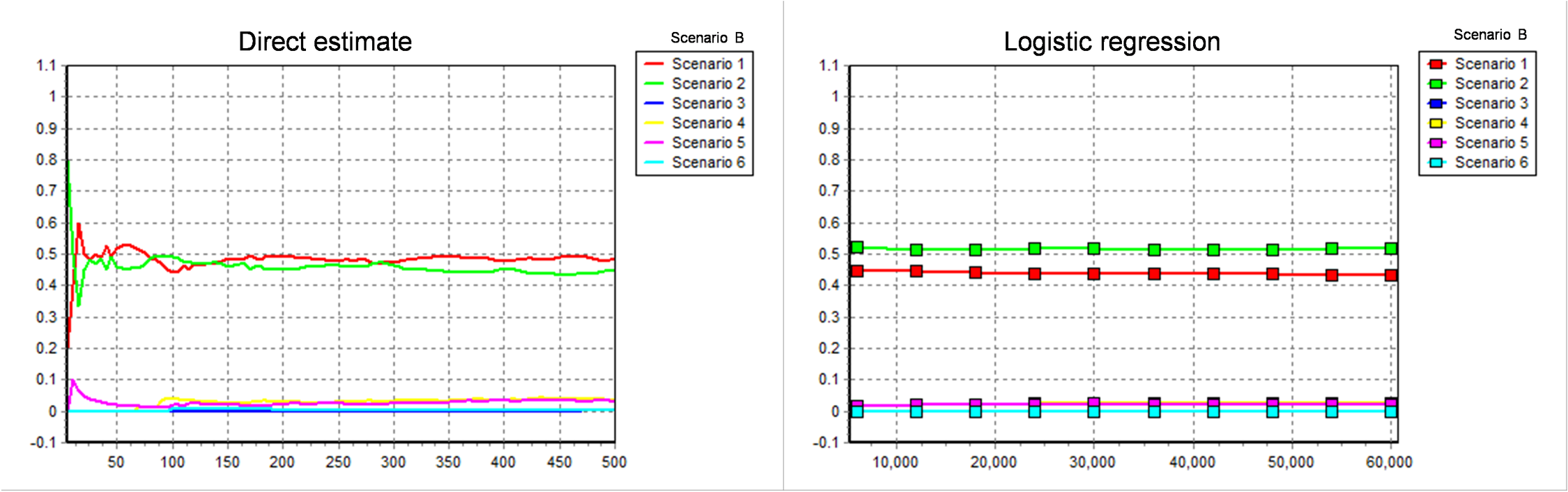

Supplement: S5 Fig — Plots output by DIYABC showing the PP (y-axis) of the second six scenarios (B1–B6) through the direct estimate (left), and the logistic regression (right) approaches, as output by DIYABC. The x-axis corresponds to the different nδ values used in the computations. The results have been obtained by performing the first analysis with four scenarios. (TIF) [file pone.0245604.s005.tif]

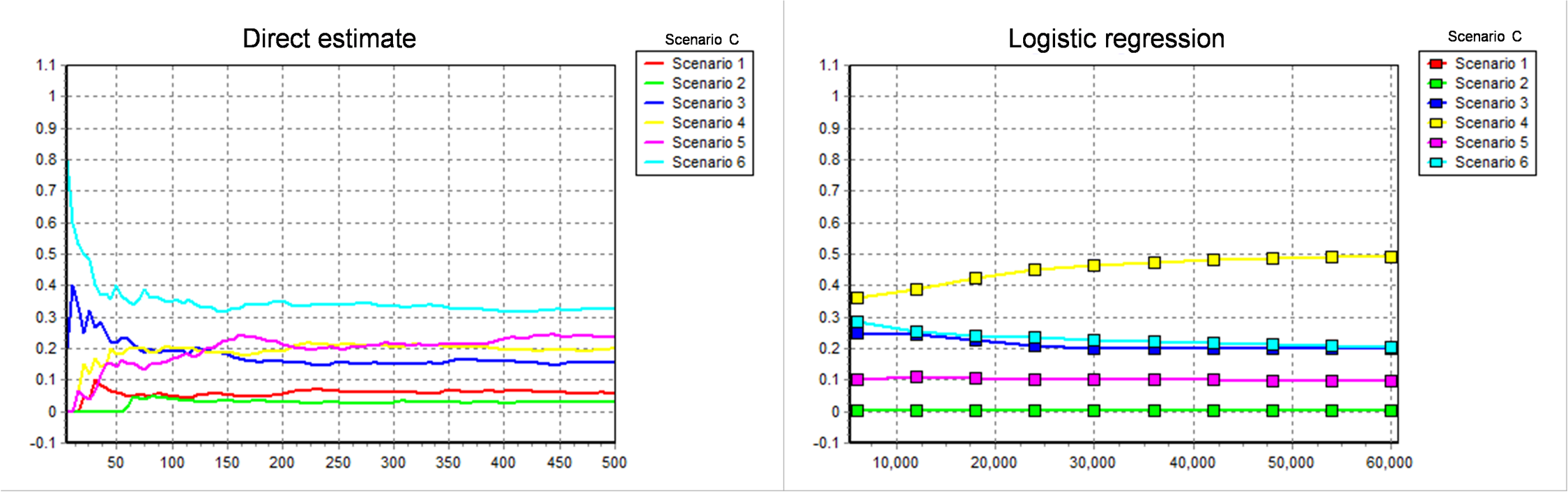

Supplement: S6 Fig — Plots output by DIYABC showing the PP (y-axis) of the third six scenarios (C1–C6) through the direct estimate (left), and the logistic regression (right) approaches, as output by DIYABC. The x-axis corresponds to the different nδ values used in the computations. The results have been obtained by performing the first analysis with four scenarios. (TIF) [file pone.0245604.s006.tif]
