# Supplementary material for: Complex evolution in Aphis gossypii group (Hemiptera: Aphididae), evidence of primary host shift and hybridization between sympatric species
Source: PLoS One. 2021 Feb 4;16(2):e0245604. doi: 10.1371/journal.pone.0245604 (PMC7861460; doi:10.1371/journal.pone.0245604)
Supplement: S1 Table — † possibly A. rhamnicola or other cryptic species †† possibly other cryptic species. (DOCX) [file pone.0245604.s007.docx]

Table S1. Collection data for 578 aphids analyzed in this study. ^†^ possibly *A. rhamnicola* or other cryptic species ^††^ possibly other cryptic species

| Pop. ID | Species | Host plant | No. | No. per site | Date | Collection site | GPS | Collected by† |
| --- | --- | --- | --- | --- | --- | --- | --- | --- |
| Ag_IL | *Aphis gossypii* | *Ilex cornuta* | 10 | 10 | 26.iv.2011 | Korea: GG, Pocheon-si, near Gwangreung | N37° 46' 33.60'' E127° 09'35.75'' | H. Kim & Y. Lee |
| Ag_CU | *Aphis gossypii* | *Cucumis sativus* | 20 | 10 | 5.vi.2011 | Korea: JB, Gimje-si | N35° 48' 18.50'' E126° 53'20.98'' | H. Kim & Y. Lee |
|  |  |  |  | 2 | 23.vi.2011 | Korea: GG, Suwon-si, Institute of Crop Science, the Sericulture Experiment Station | N37° 19' 06.38'' E126° 59' 26.89'' | H. Kim & Y. Lee |
|  |  |  |  | 2 | 7.vii.2011 | Korea: GG, Suwon-si, RDA | N37° 16' 07.20'' E126° 59' 36.60'' | H. Kim & Y. Lee |
|  |  |  |  | 2 | 14.vii.2011 | Korea: Seoul-si, Itaewon, near US Army | N37° 29' 40.20'' E126° 59' 24.00'' | Y. Lee |
|  |  |  |  | 2 | 15.viii.2011 | Korea: Seoul-si, Dongdaemun-gu, Sindap-dong | N35° 34' 23.40'' E127° 02'47.40'' | Y. Lee |
|  |  |  |  | 2 | 10.ix.2011 | Korea: GG, Icheon-si, Moga-myeon, Eonong-ri | N37° 11' 09.18'' E127° 25'54.13'' | Y. Lee |
| Ag_CM | *Aphis gossypii* | *Cucurbita moschata* | 30 | 3 | 4.vi.2011 | Korea: JB, Gimje-si, community hall | N35° 50' 22.14'' E126° 44'25.27'' | H. Kim & Y. Lee |
|  |  |  |  | 2 | 5.vi.2011 | Korea: JB, Gimje-si, Jinbong-myeon | N35° 51' 34.02'' E126° 45'53.10'' | H. Kim & Y. Lee |
|  |  |  |  | 5 | 6.vi.2011 | Korea: JB, Gimje-si, community hall | N35° 50' 22.14'' E126° 44'25.27'' | H. Kim & Y. Lee |
|  |  |  |  | 1 | 16.vi.2011 | Korea: GG, Icheon-si, Moga-myeon, Eonong-ri | N37° 10' 36.60'' E127° 25'51.60'' | Y. Lee |
|  |  |  |  | 2 | 23.vi.2011 | Korea: GG, Suwon-si, Gwonseong-gu, Senior Citizen center | N37° 16' 18.00'' E126° 59' 07.80'' | H. Kim & Y. Lee |
|  |  |  |  | 2 | 23.vi.2011 | Korea: GG, Suwon-si, Institute of Crop Science | N37° 16' 10.20'' E126° 59' 28.20'' | H. Kim & Y. Lee |
|  |  |  |  | 10 | 29.vi.2011 | Korea: GW, Gangneung-si, Jumunjin-eup, Jangseong 5th street | N37° 57' 09.60'' E128° 46'04.20'' | H. Kim & Y. Lee |
|  |  |  |  | 1 | 14.vii.2011 | Korea: GG, Suwon-si, RDA | N37° 16' 07.20'' E126° 59' 36.60'' | H. Kim & Y. Lee |
|  |  |  |  | 1 | 8.ix.2011 | Korea: Seoul-si, Seoudaemun-gu, Ahyeon-station | N37° 33' 25.20'' E126° 57'20.40'' | Y. Lee |
|  |  |  |  | 1 | 10.ix.2011 | Korea: GG, Icheon-si, Moga-myeon, Eonong-ri | N37° 10' 36.60'' E127° 25'51.60'' | Y. Lee |
|  |  |  |  | 1 | 16.ix.2011 | Korea: Seoul-si, Seoudaemun-gu, Ehwa womans University | N37° 33' 36.60'' E126° 56' 54.00'' | Y. Lee |
|  |  |  |  | 1 | 23.ix.2011 | Korea: GG, Suwon-si, RDA | N37° 16' 07.20'' E126° 59' 36.60'' | H. Kim, Y. Lee, & B. Lee |
| Ag_KA | *Aphis gossypii* | *Kalanchoe daigremontiana* | 8 | 8 | 1.v.2011 | Korea: JJ, Yeomiji Botanical garden | N33° 15' 15.04'' E126° 24'52.51'' | Y. Lee |
| Ag_SO | *Aphis gossypii* | *Solanum melongena* | 20 | 2 | 16.vi.2011 | Korea: GG, Icheon-si, Moga-myeon, Eonong-ri | N37° 10' 36.60'' E127° 25'51.60'' | Y. Lee |
|  |  |  |  | 3 | 23.vi.2011 | Korea: GG, Suwon-si, Gwonseong-gu | N37° 16' 18.00'' E126° 59' 07.80'' | H. Kim & Y. Lee |
|  |  |  |  | 7 | 23.vi.2011 | Korea: GG, Suwon-si, Institute of Crop Science, the Sericulture Experiment Station | N37° 19' 06.38'' E126° 59' 26.89'' | H. Kim & Y. Lee |
|  |  |  |  | 8 | 14.vii.2011 | Korea: GG, Suwon-si, RDA | N37° 16' 07.20'' E126° 59' 36.60'' | H. Kim & Y. Lee |
| Ag_CA | *Aphis gossypii* | *Capsicum annuum* | 25 | 10 | 16.vi.2011 | Korea: GG, Icheon-si, Moga-myeon, Eonong-ri | N37° 10' 36.60'' E127° 25'51.60'' | Y. Lee |
|  |  |  |  | 5 | 23.vi.2011 | Korea: GG, Suwon-si, Gwonseong-gu, Senior Citizen center | N37° 16' 18.00'' E126° 59' 07.80'' | H. Kim & Y. Lee |
|  |  |  |  | 10 | 29.vi.2011 | Korea: GW, Gangneung-si, Jumunjin-eup, Jangseong 5th street | N37° 57' 09.60'' E128° 46'04.20'' | H. Kim & Y. Lee |
| Ag_CP | *Aphis gossypii* | *Capsicum annuum* var*. angulosum* | 5 | 5 | 14.vii.2011 | Korea: GG, Suwon-si, RDA | N37° 16' 07.20'' E126° 59' 36.60'' | H. Kim & Y. Lee |
| Ag_PU | *Aphis gossypii* | *Punica granatum* | 27 | 7 | 17.v.2011 | Korea: GN, Hapcheon-gun, Daebyeong-myeon | N35° 31' 28.80'' E128° 00' 53.40'' | Y. Lee |
|  |  |  |  | 7 | 19.v.2011 | Korea: Seoul-si, Seoudaemun-gu, Ehwa womans University | N37° 34' 10.17'' E126° 56' 51.92'' | Y. Lee |
|  |  |  |  | 2 | 20.vi.2011 | Korea: Seoul-si, Gyeongbok-palace station | N37° 34' 34.20'' E126° 58' 32.40'' | Y. Lee |
|  |  |  |  | 1 | 25.v.2011 | Korea: Seoul-si, Seoudaemun-gu, Ehwa womans University | N37° 34' 10.17'' E126° 56' 51.92'' | Y. Lee |
|  |  |  |  | 1 | 22.vi.2011 | Korea: Seoul-si, Seoudaemun-gu, Ahyeon-station | N37° 34' 03.00'' E126° 55' 41.40'' | Y. Lee |
|  |  |  |  | 1 | 2.vii.2011 | Korea: Seoul-si, Yongsan-gu, Sukmyeong woman University | N37° 32' 39.59'' E126° 58' 23.63'' | Y. Lee |
|  |  |  |  | 8 | 15.xi.2011 | Korea: Seoul-si, Gyeongbok-palace station | N37° 34' 34.20'' E126° 58' 32.40'' | Y. Lee |
| Ag_EL | *Aphis gossypii* | *Eleutherococcus senticosus* | 10 | 10 | 13.x.2011 | Korea: JB, Byeonsan, Daemyeong resort | N35° 37' 31.80'' E126° 28' 19.20'' | H. Kim, Y. Lee, & B. Lee |
| Ag_HI | *Aphis gossypii* | *Hibiscus syriacus* | 60 | 2 | 29.iv.2011 | Korea: JJ, Jeju-si, Haean-village, Cheongok house | N33° 27' 29.40'' E126° 26'43.80'' | Y. Lee |
|  |  |  |  | 2 | 1.v.2011 | Korea: JJ, Jeju-si, Nohyeong-dong | N33° 38' 40.20'' E126° 28'01.20'' | Y. Lee |
|  |  |  |  | 2 | 1.v.2011 | Korea: JJ, Jeju-si, Haean-village, Cheongok house | N33° 28' 46.80'' E126° 28'01.20'' | Y. Lee |
|  |  |  |  | 1 | 8.v.2011 | Korea: Seoul-si, Jamsil-dong, Jamsil Jugong APT | N37° 34' 55.03'' E127° 04' 54.98'' | H. Kim |
|  |  |  |  | 3 | 13.v.2011 | Korea: GW, Hongcheon-gun, Mt. Palbongsan | N37° 41' 51.27'' E127° 41' 43.90'' | H. Kim & Y. Lee |
|  |  |  |  | 3 | 13.v.2011 | Korea: GW, Chuncheon-si, Gangwon University, Agriculture and life science department | N36° 25' 26.64'' E128° 01' 57.14'' | H. Kim & Y. Lee |
|  |  |  |  | 2 | 14.v.2011 | Korea: GW, Hoengseong-gun, Hoengseong-eup, Taegi-ro 15, Hoengseong Citi hall | N33° 30' 01.20'' E126° 30'16.80'' | H. Kim & Y. Lee |
|  |  |  |  | 10 | 16.v.2011 | Korea: GB, Goryeong-gun, Ssangrim-myeon, Ssangrim elementry school | N35° 40' 52.63'' E128° 14'31.74'' | H. Kim & Y. Lee |
|  |  |  |  | 2 | 20.v.2011 | Korea: Seoul-si, Dongdaemun-gu, Sindap-dong | N37° 34' 20.05'' E127° 02'39.03'' | Y. Lee |
|  |  |  |  | 1 | 24.iv.2011 | Korea: Seoul-si, Seoudaemun-gu, Yonsei University | N37° 33' 34.68'' E126° 56' 08.72'' | Y. Lee |
|  |  |  |  | 1 | 26.v.2011 | Korea, GG, Suwon-si, Seoul National Univiversity | N37° 15' 53.80'' E126° 58'49.03'' | H. Kim & Y. Lee |
|  |  |  |  | 5 | 6.vi.2011 | Korea: JB, Gunsan-si, Gunsan elementry school | N35° 51' 07.38'' E126° 49'05.01'' | H. Kim & Y. Lee |
|  |  |  |  | 3 | 4.vi.2011 | Korea: JB, Gimje-si, community hall | N35° 50' 22.14'' E126° 44'25.27'' | H. Kim & Y. Lee |
|  |  |  |  | 2 | 5.vi.2011 | Korea: JB, Gimje-si, Jinbong-myeon | N35° 51' 34.02'' E126° 45'53.10'' | H. Kim & Y. Lee |
|  |  |  |  | 1 | 17.vi.2011 | Korea: Seoul-si, Gwanak-gu, Seoul National University | N37° 27' 15.61'' E126° 57'13.28'' | H. Kim & Y. Lee |
|  |  |  |  | 2 | 30.vi.2011 | Korea: GW, Yangyang-gun, Namhyeon-myeon | N37° 56' 54.65'' E128° 46'41.60'' | H. Kim & Y. Lee |
|  |  |  |  | 1 | 2.vii.2011 | Korea: Seoul-si, Mapo-gu, Gongdeok-station | N37° 32' 40.12'' E126° 57'05.02'' | Y. Lee |
|  |  |  |  | 1 | 9.vii.2011 | Korea: Seoul-si, Seoudaemun-gu, Yonsei University | N37° 33' 34.68'' E126° 56' 08.72'' | Y. Lee |
|  |  |  |  | 4 | 14.vii.2011 | Korea: GG, Suwon-si, RDA | N37° 15' 49.88'' E126° 58' 26.85'' | H. Kim & Y. Lee |
|  |  |  |  | 2 | 24.iv.2011 | Korea: GG, Goyang-si, Haengsin-dong, Muwon APT | N37° 36' 59.78'' E126° 49' 55.96'' | Y. Lee |
|  |  |  |  | 2 | 24.iv.2011 | Korea: Seoul-si, Jamsil-dong, Jamsil Jugong APT | N37° 34' 55.03'' E127° 04' 54.98'' | H. Kim |
|  |  |  |  | 3 | 25.iv.2011 | Korea: Seoul-si, Seoudaemun-gu, Ehwa womans University | N37° 33' 43.18'' E126° 56' 45.03'' | H. Kim & Y. Lee |
|  |  |  |  | 1 | 28.iv.2011 | Korea, GG, Suwon-si, Seoul National Univiversity | N37° 23' 43.70'' E127° 01' 22.45'' | H. Kim & Y. Lee |
|  |  |  |  | 2 | 29.iv.2011 | Korea: JJ, Jeju-si, Yena hospital | N33° 30' 01.20'' E126° 30'16.80'' | Y. Lee |
|  |  |  |  | 2 | 29.iv.2011 | Korea: JJ, Jeju-si, Seoyeon-ro | N33° 29' 54.60'' E126° 29'59.40'' | Y. Lee |
| Ag_HR | *Aphis gossypii* | *Hibiscus rosa-sinensis* | 10 | 10 | 22.iv.2011 | Korea: JN, Wando-gun, Wando-arboretum | N34° 21' 31.95'' E126° 40' 09.00'' | H. Kim & Y. Lee |
| Ag_EU | *Aphis gossypii* | *Euonymus trapococca* | 10 | 5 | 19.v.2011 | Korea: Seoul-si, Seoudaemun-gu, Ehwa womans University | N37° 33' 44.38'' E126° 56' 39.00'' | Y. Lee |
|  |  |  |  | 5 | 20.v.2011 | Korea: Seoul-si, Dongdaemun-gu, Sindap station | N37° 34' 16.20'' E127° 02'42.00'' | Y. Lee |
| Ag_EJ | *Aphis gossypii* | *Euonymus japonicus* | 20 | 10 | 5.vi.2011 | Korea: JB, Gimje-si, Jinbong-myeon | N35° 51' 34.02'' E126° 45'53.10'' | H. Kim & Y. Lee |
|  |  |  |  | 10 | 9.vi.2011 | Korea: Seoul-si, Dongdaemun-gu, Sindap-dong | N37° 34' 20.05'' E127° 02'39.03'' | Y. Lee |
| Ag_CI | *Aphis gossypii* | *Citrus unshiu* | 20 | 5 | 30..iv.2011 | Korea: JJ, Seoguipo-si, Namwon-eup, Jeju agriculture and ecology senter | N33° 17' 20.40'' E126° 37'24.60'' | Y. Lee |
|  |  |  |  | 15 | 1.v.2011 | Korea: JJ, Jeju-si, Jeju international airport | N33° 30' 01.16'' E126° 29'51.33'' | Y. Lee |
| Ag_FO | *Aphis gossypii* | *Forsythia koreana* | 10 | 10 | 19.v.2011 | Korea: Seoul-si, Seoudaemun-gu, Ehwa womans University | N37° 33' 48'.41' E126° 56' 41.28'' | Y. Lee |
| Ag_CE | *Aphis gossypii* | *Celastrus orbiculatus* | 20 | 4 | 10.ix.2011 | Korea: GG, Icheon-si, Moga-myeon, Eonong-ri | N37° 10' 36.60'' E127° 25'51.60'' | Y. Lee |
|  |  |  |  | 4 | 16.vi.2011 | Korea: GG, Icheon-si, Moga-myeon, Eonong-ri | N37° 10' 36.60'' E127° 25'51.60'' | Y. Lee |
|  |  |  |  | 5 | 19.v.2011 | Korea: Seoul-si, Seoudaemun-gu, Ehwa womans University | N37° 33' 58.93'' E126° 56'54.85'' | Y. Lee |
|  |  |  |  | 1 | 26.v.2011 | Korea, GG, Suwon-si, Seoul National Univ. arboretum | N37° 26' 24.99'' E127° 01'58.72'' | H. Kim & Y. Lee |
|  |  |  |  | 6 | 26.v.2011 | Korea, GG, Suwon-si, Seoul National Univiversity | N37° 15' 53.80'' E126° 58'49.03'' | H. Kim & Y. Lee |
| Ag_ER | *Aphis gossypii* | *Erigeron annuus* | 10 | 10 | 4.vi.2011 | Korea: JB, Gunsan-si, Gunsan elementry school | N35° 51' 07.38'' E126° 49'05.01'' | H. Kim & Y. Lee |
| Ag_SN | *Aphis gossypii* | *Sonchus oleraceus* | 8 | 8 | 5.vi.2011 | Korea: JB, Gimje-si, Jinbong-myeon | N35° 51' 34.02'' E126° 45'53.10'' | H. Kim & Y. Lee |
| Ag_CO | *Aphis gossypii* | *Cosmos bipinnatus* | 18 | 2 | 23.vi.2011 | Korea: GG, Suwon-si, Gwonseong-gu, CU convenience store | N37° 15' 24.00'' E127° 02' 09.00'' | H. Kim & Y. Lee |
|  |  |  |  | 8 | 5.vii.2011 | Korea: Seoul, Yeouido Park, Nanji-camping ground | N37° 34' 13.80'' E126° 52' 19.80'' | H. Kim & Y. Lee |
|  |  |  |  | 4 | 16.ix.2011 | Korea: Seoul-si, Seoudaemun-gu, Ehwa womans University | N37° 33' 36.60'' E126° 56' 54.00'' | Y. Lee |
|  |  |  |  | 4 | 23.ix.2011 | Korea: GG, Suwon-si, RDA | N37° 16' 07.20'' E126° 59' 36.60'' | H. Kim, Y. Lee, & B. Lee |
| Ag_CL | *Aphis gossypii* | *Clinopodium chinense* var*. parviflorum* | 10 | 10 | 13.x.2011 | Korea: JB, Byeonsan, Daemyeong resort | N35° 37' 54.60'' E126° 28' 19.20'' | H. Kim, Y. Lee, & B. Lee |
| Ag_CT | *Aphis gossypii* | *Catalpa ovata* | 10 | 10 | 20.viii.2011 | UK: London, Big Ben | N51° 30' 00.60'' E0° 07'34.20'' | Y. Lee |
| Ag_CJ | *Aphis gossypii* | *Callicarpa japonica* | 10 | 10 | 23.ix.2011 | Korea, GG, Suwon-si, Seoul National Univ. arboretum | N37° 15' 43.32'' E126° 58'57.48'' | H. Kim, Y. Lee, & B. Lee |
| Ag_RH | *Aphis gossypii* | *Rhamnus davurica* | 20 | 1 | 21.iv.2011 | Korea: CN, Sejong-si, Geumgang arboretum | N36° 26' 11.7'' E127° 14'20.8'' | H. Kim & Y. Lee |
|  |  |  |  | 7 | 28.iv.2011 | Korea: GG, Osan-si, Mulhyanggi-arboretum | N37° 27' 13.26'' E126° 57'36.49'' | H. Kim & Y. Lee |
|  |  |  |  | 1 | 3.xi.2011 | Korea: GG, Osan-si, Mulhyanggi-arboretum | N37° 26' 24.99'' E127° 01'58.72'' | H. Kim & Y. Lee |
|  |  |  |  | 10 | 28.iv.2011 | Korea, GG, Suwon-si, Seoul National Univ. arboretum | N37° 26' 24.99'' E127° 01'58.72'' | H. Kim & Y. Lee |
|  |  |  |  | 1 | 3.xi.2011 | Korea, GG, Suwon-si, Seoul National Univ. arboretum | N37° 26' 24.99'' E127° 01'58.72'' | H. Kim & Y. Lee |
| Ag_SE | *Aphis gossypii* ^†^ | *Sedum kamtschaticum* | 10 | 10 | 14.v.2011 | Korea: GW, Hoengseong-gun, Holocene | N37° 31' 33.00'' E128° 09'01.80'' | H. Kim & Y. Lee |
| Ag_PE | *Aphis gossypii* ^†^ | *Perilla frutescens* var*. frutescens* | 10 | 10 | 14.x.2011 | Korea: JB, Buan-gun, Byeonsan-myeon | N35° 41' 31.77'' E126° 33' 43.75'' | H. Kim, Y. Lee, & B. Lee |
| Ag_YO | *Aphis gossypii* ^††^ | *Youngia sonchifolia* | 11 | 2 | 8.vi.2011 | Korea: Seoul-si, Dongdaemun-gu, Sindap-dong | N37° 34' 20.05'' E127° 02'39.03'' | Y. Lee |
|  |  |  |  | 2 | 9.vi.2011 | Korea: Seoul-si, Seoudaemun-gu, Ehwa womans University | N37° 33' 43.18'' E126° 56' 45.03'' | Y. Lee |
|  |  |  |  | 2 | 13.vi.2011 | Korea: Seoul-si, Dongdaemun-gu, Sindap-dong | N37° 34' 20.05'' E127° 02'39.03'' | Y. Lee |
|  |  |  |  | 2 | 17.vi.2011 | Korea: Seoul-si, Gwanak-gu, Seoul National University | N37° 27' 15.61'' E126° 57'13.28'' | H. Kim & Y. Lee |
|  |  |  |  | 3 | 20.vi.2011 | Korea: Seoul-si, Seoudaemun-gu, Ehwa womans University | N37° 33' 43.18'' E126° 56' 45.03'' | Y. Lee |
| Ag_IX | *Aphis gossypii*. ^††^ | *Ixeris strigosa* | 11 | 8 | 7.vii.2011 | Korea: GG, Suwon-si, RDA | N37° 16' 07.20'' E126° 59' 36.60'' | H. Kim & Y. Lee |
|  |  |  |  | 3 | 30.viii.2011 | Korea: Seoul-si, Gwanak-gu, Seoul National University | N37° 27' 15.61'' E126° 57'13.28'' | H. Kim & Y. Lee |
| Ar_RH | *Aphis rhamnicola* | *Rhamnus davurica* | 8 | 8 | 28.iv.2011 | Korea: GG, Osan-si, Mulhyanggi-arboretum | N37° 27' 13.26'' E126° 57'36.49'' | H. Kim & Y. Lee |
| Ar_CO | *Aphis rhamnicola* | *Commelina communis* | 30 | 10 | 10.ix.2011 | Korea: GG, Icheon-si, Moga-myeon, Eonong-ri | N37° 10' 36.60'' E127° 25'51.60'' | Y. Lee |
|  |  |  |  | 5 | 16.ix.2011 | Korea: Seoul-si, Seoudaemun-gu, Ehwa womans University | N37° 33' 36.60'' E126° 56' 54.00'' | Y. Lee |
|  |  |  |  | 5 | 23.ix.2011 | Korea: GG, Suwon-si, RDA | N37° 16' 07.20'' E126° 59' 36.60'' | H. Kim, Y. Lee, & B. Lee |
|  |  |  |  | 5 | 14.x.2011 | Korea: JB, Byeonsan, Daemyeong resort | N35° 37' 54.60'' E126° 28' 19.20'' | H. Kim, Y. Lee, & B. Lee |
|  |  |  |  | 5 | 15.x.2011 | Korea: JB, Buan-gun, Byeonsan-myeon | N35° 41' 31.77'' E126° 33' 43.76'' | H. Kim, Y. Lee, & B. Lee |
| Ar_LE | *Aphis rhamnicola* | *Leonurus japonicus* | 10 | 10 | 25.viii.2011 | Korea: GN, Geoje-si, Dongbu-myeon | N34° 49' 16.60" E128 °36' 25.50" | H. Kim & Y. Lee |
| Ar_PH | *Aphis rhamnicola* | *Phryma leptostachy* | 7 | 7 | 15.x.2011 | Korea: CN, Boryeong-si, Mt. Oseosan | N36° 27' 13.86" E126° 40' 39.56" | H. Kim, Y. Lee, & B. Lee |
| Ar_ST | *Aphis rhamnicola* | *Stellaria media* | 6 | 6 | 7.iv.2011 | Korea: GN, Masan-si, Jincheon-myeon | N35° 9' 33.76" E128° 22' 16.75" | H. Kim & Y. Lee |
| Ar_LY | *Aphis rhamnicola* | *Lysimachia coreana* | 10 | 10 | 30.vi.2011 | Korea: GW, Yangyang-gun, Namhyeon-myeon | N37° 56' 54.65" E128° 46' 41.60" | H. Kim & Y. Lee |
| Ar_CB | *Aphis rhamnicola* | *Capsella bursa-pastoris* | 24 | 1 | 29.iv.2011 | Korea: JJ, Jeju-si, Hallim-eup | N33° 24' 37.58" E 126° 16' 1.85" | Y. Lee |
|  |  |  |  | 8 | 4.vi.2011 | Korea: CN, Taean-gun, Cheonripo arboretum | N 36° 47' 55.41" E126° 8' 57.16" | H. Kim & Y. Lee |
|  |  |  |  | 5 | 29.iv.2011 | Korea: JJ, Jeju-si, Hallim-eup | N33° 24' 37.58" E 126° 16' 1.85" | Y. Lee |
|  |  |  |  | 10 | 15.v.2011 | Korea: GN, Sacheon-si | N35° 0' 13.60" E128° 3' 51.07" | H. Kim & Y. Lee |
| Ar_VE | *Aphis rhamnicola* | *Veronica insularis* | 10 | 10 | 8.vi.2011 | Korea: Daegu-si, Buk-gu, Chilgok-gun, Agricultural Technology Center | N35° 55' 5.15" E128° 15' 9.44" | H. Kim & Y. Lee |
| Ar_RU | *Aphis rhamnicola* | *Rubia akane* | 40 | 10 | 7.vii.2011 | Korea, GG, Suwon-si, Seoul National Univ. arboretum | N37° 16' 07.20'' E126° 59' 36.60'' | H. Kim & Y. Lee |
|  |  |  |  | 10 | 10.ix.2011 | Korea: GG, Icheon-si, Moga-myeon, Eonong-ri | N37° 10' 36.60'' E127° 25'51.60'' | Y. Lee |
|  |  |  |  | 10 | 1.v.2011 | Korea: JJ, Jeju-si, Hallim-eup | N33° 24' 37.58" E126° 16' 1.85" | Y. Lee |
|  |  |  |  | 10 | 30.vi.2011 | Korea: GW, Yangyang-gun, Seo-myeon | N37° 56' 42.05" E128° 31' 6.19" | H. Kim & Y. Lee |
|  |  | TOTAL | 578 | 578 |  |  |  |  |
