# Supplementary material for: Complex evolution in Aphis gossypii group (Hemiptera: Aphididae), evidence of primary host shift and hybridization between sympatric species
Source: PLoS One. 2021 Feb 4;16(2):e0245604. doi: 10.1371/journal.pone.0245604 (PMC7861460; doi:10.1371/journal.pone.0245604)
Supplement: S2 Table — Values in bold indicate the proportions of individuals assigned to the source population (self-assignment). Values less than 0.001 were excluded from the table. (DOCX) [file pone.0245604.s008.docx]

Table S2. Mean assignment rate of individuals into (rows) and from (columns) each population using GeneClass 2 (Piry et al. 2004). Values in bold indicate the proportions of individuals assigned to the source population (self-assignment). Values less than 0.001 were excluded from the table

|  | Ag_CA | Ag_CE | Ag_CI | Ag_CJ | Ag_CL | Ag_CM | Ag_CO | Ag_CP | Ag_CT | Ag_CU | Ag_EJ | Ag_EL | Ag_ER | Ag_EU | Ag_FO | Ag_HI | Ag_HR | Ag_IL | Ag_KA |
| --- | --- | --- | --- | --- | --- | --- | --- | --- | --- | --- | --- | --- | --- | --- | --- | --- | --- | --- | --- |
| /Ag_CA | **0.731** |  | 0.069 | 0.003 |  | 0.008 |  | 0.742 |  | 0.000 | 0.001 |  |  | 0.001 |  | 0.011 |  |  | 0.001 |
| /Ag_CE |  | **0.432** | 0.042 | 0.003 | 0.000 | 0.032 | 0.007 |  |  |  | 0.132 | 0.061 | 0.001 | 0.053 | 0.039 | 0.104 |  | 0.000 | 0.000 |
| /Ag_CI | 0.000 | 0.051 | **0.519** | 0.002 |  | 0.056 | 0.026 | 0.001 |  | 0.004 | 0.042 | 0.001 | 0.008 | 0.073 | 0.048 | 0.092 |  | 0.000 | 0.012 |
| /Ag_CJ |  | 0.001 | 0.006 | **0.438** |  | 0.005 | 0.013 |  |  |  | 0.026 | 0.001 | 0.000 | 0.005 | 0.038 | 0.020 |  | 0.001 |  |
| /Ag_CL |  | 0.548 | 0.079 | 0.002 | **0.557** | 0.011 |  |  |  |  | 0.017 | 0.448 |  | 0.309 |  | 0.019 |  |  | 0.000 |
| /Ag_CM | 0.002 | 0.052 | 0.086 | 0.008 |  | **0.525** | 0.008 | 0.012 |  | 0.489 | 0.139 | 0.014 | 0.000 | 0.117 | 0.016 | 0.353 |  | 0.003 | 0.072 |
| /Ag_CO |  | 0.015 | 0.061 | 0.011 |  | 0.028 | **0.426** |  |  | 0.002 | 0.297 | 0.001 | 0.057 | 0.085 | 0.428 | 0.237 |  | 0.004 | 0.000 |
| /Ag_CP | 0.532 |  | 0.052 | 0.004 |  | 0.008 |  | **0.713** |  |  |  |  |  | 0.000 |  | 0.016 |  |  | 0.001 |
| /Ag_CT |  | 0.253 | 0.170 | 0.006 |  | 0.033 | 0.035 |  | **0.591** | 0.001 | 0.217 | 0.002 | 0.041 | 0.133 | 0.071 | 0.097 |  | 0.000 | 0.000 |
| /Ag_CU | 0.000 | 0.000 | 0.047 | 0.008 |  | 0.490 | 0.000 |  |  | **0.569** | 0.064 | 0.004 |  | 0.035 | 0.000 | 0.246 |  | 0.002 | 0.011 |
| /Ag_EJ |  | 0.051 | 0.088 | 0.009 |  | 0.077 | 0.214 |  |  | 0.001 | **0.498** | 0.002 | 0.080 | 0.148 | 0.206 | 0.258 |  | 0.002 | 0.000 |
| /Ag_EL |  |  |  |  |  |  |  |  |  |  |  |  |  |  |  |  |  |  |  |
| /Ag_ER |  | 0.012 | 0.295 | 0.008 |  | 0.019 | 0.645 |  |  |  | 0.452 |  | **0.452** | 0.206 | 0.513 | 0.358 |  | 0.000 |  |
| /Ag_EU |  | 0.037 | 0.094 | 0.005 |  | 0.035 | 0.112 |  |  | 0.000 | 0.180 | 0.003 | 0.058 | **0.384** | 0.110 | 0.210 |  | 0.000 |  |
| /Ag_FO |  | 0.013 | 0.027 | 0.008 |  | 0.028 | 0.445 |  |  | 0.000 | 0.363 | 0.000 | 0.026 | 0.037 | **0.376** | 0.275 |  | 0.002 |  |
| /Ag_HI | 0.000 | 0.033 | 0.060 | 0.009 |  | 0.125 | 0.110 |  |  | 0.024 | 0.223 | 0.008 | 0.008 | 0.126 | 0.110 | **0.479** | 0.000 | 0.005 | 0.007 |
| /Ag_HR |  | 0.012 | 0.012 | 0.010 |  | 0.126 | 0.038 |  |  | 0.016 | 0.139 | 0.004 | 0.002 | 0.008 | 0.063 | 0.270 | **0.461** | 0.004 | 0.001 |
| /Ag_IL |  | 0.001 | 0.020 | 0.009 |  | 0.052 | 0.023 |  |  | 0.006 | 0.074 |  |  | 0.014 | 0.015 | 0.168 |  | **0.702** | 0.032 |
| /Ag_KA |  | 0.005 | 0.126 | 0.004 |  | 0.106 |  |  |  | 0.006 | 0.009 | 0.004 |  | 0.013 |  | 0.167 |  | 0.000 | **0.570** |
| /Ag_PU |  | 0.015 | 0.045 | 0.004 |  | 0.042 | 0.144 |  | 0.000 | 0.056 | 0.118 | 0.000 | 0.014 | 0.050 | 0.174 | 0.122 |  | 0.001 | 0.000 |
| /Ag_RH |  | 0.003 | 0.008 | 0.002 |  | 0.004 | 0.012 |  |  |  | 0.024 | 0.001 | 0.003 | 0.004 | 0.031 | 0.024 |  |  |  |
| /Ag_SN |  | 0.080 | 0.303 | 0.010 |  | 0.053 | 0.581 |  |  | 0.001 | 0.671 | 0.003 | 0.343 | 0.281 | 0.503 | 0.422 |  | 0.002 | 0.001 |
| /Ag_SO | 0.393 | 0.009 | 0.080 | 0.004 |  | 0.025 | 0.044 | 0.395 |  | 0.001 | 0.086 | 0.000 | 0.011 | 0.039 | 0.084 | 0.079 |  | 0.002 | 0.000 |
| /Ag_SE |  |  |  | 0.000 |  |  |  |  |  |  |  |  |  |  |  |  |  |  |  |
| /Ag_PE |  |  |  |  |  |  |  |  |  |  |  |  |  |  |  |  |  |  |  |
| /Ag_IX |  |  |  | 0.000 |  |  |  |  |  |  |  |  |  |  |  |  |  |  |  |
| /Ag_YO |  |  |  |  |  |  |  |  |  |  |  |  |  |  |  |  |  |  |  |
| /Ar_CB |  |  | 0.002 | 0.001 |  | 0.002 |  |  |  | 0.000 | 0.001 | 0.000 |  | 0.001 |  | 0.070 |  |  | 0.000 |
| /Ar_CO |  |  |  | 0.000 |  |  |  |  |  |  |  |  |  |  |  |  |  |  |  |
| /Ar_LE |  |  |  | 0.000 |  |  |  |  |  |  |  |  |  |  |  |  |  |  |  |
| /Ar_LY |  |  |  |  |  |  |  |  |  |  |  |  |  |  |  |  |  |  |  |
| /Ar_PH |  |  |  | 0.073 |  |  |  |  |  |  |  |  |  |  |  |  |  |  |  |
| /Ar_RH |  |  |  | 0.001 |  |  |  |  |  |  |  |  |  |  |  |  |  |  |  |
| /Ar_RU |  |  |  | 0.000 |  |  |  |  |  |  |  |  |  | 0.000 |  | 0.001 |  |  |  |
| /Ar_ST |  | 0.019 | 0.028 | 0.011 |  | 0.186 | 0.005 |  |  | 0.069 | 0.133 | 0.019 |  | 0.139 | 0.009 | 0.531 |  | 0.005 | 0.102 |
| /Ar_VE |  |  | 0.000 | 0.001 |  |  |  |  |  |  |  |  |  | 0.000 |  | 0.013 |  |  |  |

Table S2. (continued)

|  | Ag_PU | Ag_RH | Ag_SN | Ag_SO | Ag_SE | Ag_PE | Ag_IX | Ag_YO | Ar_CB | Ar_CO | Ar_LE | Ar_LY | Ar_PH | Ar_RH | Ar_RU | Ar_ST | Ar_VE |
| --- | --- | --- | --- | --- | --- | --- | --- | --- | --- | --- | --- | --- | --- | --- | --- | --- | --- |
| /Ag_CA | 0.008 | 0.111 |  | 0.732 |  |  |  |  |  |  |  |  | 0.000 |  |  |  |  |
| /Ag_CE | 0.120 | 0.357 | 0.063 | 0.017 |  |  |  |  |  |  |  |  | 0.001 |  |  |  |  |
| /Ag_CI | 0.195 | 0.485 | 0.042 | 0.155 |  |  |  |  |  |  |  |  | 0.000 |  |  |  |  |
| /Ag_CJ | 0.085 | 0.283 | 0.045 | 0.003 |  |  |  |  |  |  |  |  | 0.123 |  |  |  |  |
| /Ag_CL | 0.060 | 0.269 | 0.015 | 0.003 |  |  |  |  |  |  |  |  | 0.001 |  |  |  |  |
| /Ag_CM | 0.164 | 0.354 | 0.017 | 0.112 |  |  |  |  | 0.002 |  |  | 0.001 | 0.001 | 0.000 | 0.000 | 0.004 | 0.003 |
| /Ag_CO | 0.391 | 0.776 | 0.186 | 0.035 |  |  |  |  | 0.000 |  |  |  | 0.000 |  |  | 0.000 | 0.000 |
| /Ag_CP | 0.005 | 0.050 |  | 0.590 |  |  |  |  |  |  |  |  | 0.000 |  |  |  |  |
| /Ag_CT | 0.385 | 0.282 | 0.094 | 0.040 |  |  |  |  |  |  |  |  | 0.000 |  |  |  |  |
| /Ag_CU | 0.169 | 0.235 | 0.003 | 0.111 |  |  |  |  | 0.000 |  |  |  | 0.001 |  |  | 0.000 | 0.000 |
| /Ag_EJ | 0.329 | 0.596 | 0.192 | 0.067 |  |  |  |  |  |  |  |  | 0.001 |  |  |  | 0.000 |
| /Ag_EL |  |  |  |  |  |  |  |  |  |  |  |  |  |  |  |  |  |
| /Ag_ER | 0.602 | 0.886 | 0.399 | 0.142 |  |  |  |  |  |  |  |  |  |  |  |  |  |
| /Ag_EU | 0.239 | 0.620 | 0.050 | 0.041 |  |  |  |  | 0.000 |  |  | 0.003 | 0.001 |  | 0.000 |  | 0.000 |
| /Ag_FO | 0.464 | 0.867 | 0.191 | 0.040 |  |  |  |  |  |  |  |  | 0.000 |  |  |  |  |
| /Ag_HI | 0.182 | 0.433 | 0.081 | 0.078 |  |  |  |  | 0.013 |  |  | 0.009 | 0.002 | 0.000 | 0.000 | 0.013 | 0.006 |
| /Ag_HR | 0.172 | 0.660 | 0.060 | 0.012 |  |  |  |  |  |  |  |  | 0.001 |  |  |  |  |
| /Ag_IL | 0.218 | 0.277 | 0.045 | 0.095 |  |  |  |  |  |  |  |  | 0.001 |  |  |  |  |
| /Ag_KA | 0.114 | 0.184 | 0.001 | 0.044 |  |  |  |  |  |  |  |  | 0.001 |  |  |  | 0.001 |
| /Ag_PU | **0.460** | 0.655 | 0.089 | 0.051 |  |  |  |  |  |  |  |  | 0.000 |  |  |  |  |
| /Ag_RH | 0.076 | **0.382** | 0.012 | 0.004 |  |  |  |  |  | 0.001 |  |  | 0.000 |  |  |  |  |
| /Ag_SN | 0.686 | 0.788 | **0.465** | 0.179 |  |  |  |  |  |  |  |  | 0.000 |  |  |  |  |
| /Ag_SO | 0.083 | 0.314 | 0.029 | **0.575** |  |  |  |  |  |  |  |  | 0.001 |  |  |  |  |
| /Ag_SE | 0.000 | 0.020 | 0.000 |  | **0.416** |  |  |  |  |  |  |  | 0.001 |  |  |  |  |
| /Ag_PE |  |  |  |  |  | **0.583** |  |  |  |  |  |  | 0.012 |  |  |  |  |
| /Ag_IX |  |  |  |  |  |  | **0.351** | 0.129 |  |  |  |  | 0.000 |  |  |  |  |
| /Ag_YO |  |  |  |  |  |  | 0.021 | **0.333** |  |  |  |  | 0.001 |  |  |  |  |
| /Ar_CB | 0.000 | 0.016 | 0.000 | 0.000 |  |  |  |  | **0.464** |  | 0.000 | 0.405 | 0.002 |  | 0.004 | 0.014 | 0.186 |
| /Ar_CO |  | 0.107 |  |  |  |  |  |  |  | **0.388** | 0.000 | 0.001 | 0.002 | 0.018 |  |  | 0.000 |
| /Ar_LE |  | 0.001 |  |  |  |  |  |  |  |  | **0.387** |  | 0.002 |  | 0.000 |  |  |
| /Ar_LY |  |  |  |  |  |  |  |  |  |  |  |  |  |  |  |  |  |
| /Ar_PH |  | 0.283 |  | 0.001 |  | 0.001 |  |  |  | 0.001 |  |  | **0.565** | 0.002 | 0.000 |  |  |
| /Ar_RH |  | 0.284 |  |  |  |  |  |  |  | 0.117 |  |  | 0.002 | **0.322** |  |  |  |
| /Ar_RU |  | 0.003 |  |  |  |  |  |  | 0.004 |  | 0.001 | 0.025 | 0.002 |  | **0.491** | 0.000 | 0.011 |
| /Ar_ST | 0.035 | 0.212 | 0.025 | 0.013 |  |  |  |  | 0.268 |  | 0.001 | 0.216 | 0.003 |  | 0.008 | **0.482** | 0.227 |
| /Ar_VE |  | 0.284 |  |  |  |  |  |  | 0.344 |  | 0.001 | 0.306 | 0.002 |  | 0.012 |  | **0.441** |
