# Supplementary material for: Complex evolution in Aphis gossypii group (Hemiptera: Aphididae), evidence of primary host shift and hybridization between sympatric species
Source: PLoS One. 2021 Feb 4;16(2):e0245604. doi: 10.1371/journal.pone.0245604 (PMC7861460; doi:10.1371/journal.pone.0245604)
Supplement: Attachment — Submitted filename: PONE-D-20-16606_R2-1.pdf [file pone.0245604.s012.pdf]

PLOS ONE

# Complex evolution in Aphis gossypii group (Hemiptera: Aphididae), evidence of primary host shift and hybridization between sympatric species

--Manuscript Draft--

| | |
|---|---|
| **Manuscript Number:** | PONE-D-20-16606R2 |
| **Article Type:** | Research Article |
| **Full Title:** | Complex evolution in Aphis gossypii group (Hemiptera: Aphididae), evidence of primary host shift and hybridization between sympatric species |
| **Short Title:** | Complex evolution in Aphis gossypii group |
| **Corresponding Author:** | Hyojoong Kim<br>Kunsan National University<br>Gunsan, KOREA, REPUBLIC OF |
| **Keywords:** | host alternation, loss of primary host, molecular ecology, population genetics, speciation |
| **Abstract:** | Aphids provide a good model system to understand the ecological speciation concept, since the majority of the species are host-specific, and they spend their entire lifecycle on certain groups of host plants. Aphid species that apparently have wide host plant ranges have often turned out to be complexes of host-specialized biotypes. Here we investigated the various host-associated populations of the two recently diverged species, Aphis gossypii and A. rhamnicola , having multiple primary hosts, to understand the complex evolution with host-associated speciation. Using mitochondrial DNA marker and nine microsatellite loci, we reconstructed the haplotype network, and analyzed the genetic structure and relationships. Approximate Bayesian computation was also used to infer the ancestral primary host and host-associated divergence, which resulted in Rhamnus being the most ancestral host for A. gossypii and A. rhamnicola . As a result, Aphis gossypii and A. rhamnicola do not randomly use their primary and secondary host plants; rather, certain biotypes use only some secondary and specific primary hosts. Some biotypes are possibly in a diverging state through specialization to specific primary hosts. Our results also indicate that a new heteroecious race can commonly be derived from the heteroecious ancestor, showing strong evidence of ecological specialization through a primary host shift in both A. gossypii and A. rhamnicola . Interestingly, A. gossypii and A. rhamnicola shared COI haplotypes with each other, thus there is a possibility of introgression by hybridization between them by cross-sharing same primary hosts. Our results contribute to a new perspective in the study of aphid evolution by identifying complex evolutionary trends in the gossypii sensu lato complex. |
| **Order of Authors:** | Yerim Lee |
| | Thomas Thieme |
| | Hyojoong Kim |
| **Opposed Reviewers:** | |
| **Response to Reviewers:** | [1]<br>Reviewer 1: The potential effects of incomplete lineage sorting should not be ignored in the Discussion. You should consider stating that incomplete lineage sorting cannot be discounted as a cause before revealing that interspecific matings have been observed (which gives more credence to the hybridization hypothesis).<br><br>Editor: In other respects, this revision failed to address justified criticism by the reviewers. Reviewer 2 made the perfectly valid point that shared mitochondrial haplotypes between A. gossypii and A. rhamnicola (and between other potential species showing strong differentiation with nuclear markers) could be the result of incomplete lineage sorting rather than evidence of ongoing hybridization. I cannot judge which explanation is more likely, and the authors have every right to discuss why they consider hybridization more likely based on the available evidence. But this is not done in the paper. There is some unconvincing rebuttal in the cover letter and the term incomplete lineage sorting does not even show up in the paper's Discussion. This is |

Powered by Editorial Manager® and ProduXion Manager® from Aries Systems Corporation

not thorough.
>>
[Response and rebuttal]
We have reflected in the manuscript what the reviewer and editor pointed out. As pointed out by reviews comments, doing lineage sorting by COI haplotype results is likely unclear at the first submitted manuscript. Since A. gossypii and A. rhamnicola cross-sharing haplotypes in several host plants, there is a problem with lineage sorting or DNA identification with only COI haplotypes (barcoding). However, I am not saying clearly that our sample is misidentified with the two species, A. gossypii and A. rhamnicola. In particular, it was revealed that genotyping by microsatellite was the only way to distinguish between these two evolutionarily intermediate species, even for organizing the sampled individuals in HAP type and utilizing them for analysis. Accepting these editor and reviewer's opinions, the identification content by COI haplotype (barcoding) was deleted and supplemented with the concept of species lineage (group) sorting instead. Therefore, 'Groups' sorted by lineage in the table are specified and subdivided into A. gossypii Group1, A. g. Group 2, A. rhamnicola Group 1, A. r. Group 2, A. r. Group 3. In addition, according to our new lineage sorting, species names were deleted in Fig 2 and Fig 4. since specifying the species creates a rather confusing situation and misunderstanding of original purpose of study. The changes in the text are as follows.
"The two Aphis species, A. gossypii and A. rhamnicola, we study here are not only very similar in morphology, but also share several host plants due to the polyphagy. Although we performed species identification through morphology and host plant relationships as a first step and also tested DNA barcoding for all individuals collected on their shared host plants (eg Capsella, Rhamnus, and Rubia), we found that there were a lot of the haplotypes cross-shared between A. gossypii and A. rhamnicola (see Results). Therefore, instead of identifying the species with 36 HAPs, we applied the dominant assignment (white, green, blue, red, dark blue) of the genetic structure (K =3, 4, 5) by STRUCTURE as well as the PCoA results (see Results) to sort their lineags into five groups as Aphis gossypii Group 1, A. g. Group 2, A. rhmanicola Group 1, A. r. Group 2 and A. r. Group 3 (Table 1). 'Aphis gossypii' and'A. rhamincola', which are mentioned later, are meant to include all group lin eages containing the HAPs assigned by the results. Table S3 shows detailed information for lineage sorted samples used in DNA analyses." -> LINE 202-211, Table 1, Fig2, Fig4 revised
In addition, the content of HAP mixing in the intermediating host plant based on COI or 16S hybridization is sufficiently mentioned in the discussion by citing a paper by Lozier et al. [89]. Please check the contents at the end of the discussion. In relation to COI hybridization, hybridized population of Hyalopterus pruni has already found by Lozier et al. [89]. It has been explored and studied by and presented the second observation in aphids and corroborating results in our study. It is emphasized once again that the phenomenon of cross-sharing of COI haplotypes between the two species is by no means due to errors in lineage sorting.

[2]
Reviewer 1 continues to be concerned with over- or mis-interpretation of your population genetics data. You should address the following points in your revision:
The conclusions you draw about the cause of heterozygote excess or heterozygote deficit (lines 455-464) are not appropriate for the Results section. If retained, this section should be moved to the Discussion, and the comments of Reviewer 1 addressed.
Editor: Similarly, reviewer 1 criticized the misleading interpretation of some standard population genetic indices (heterozygosities etc.). While the authors did check whether some of the deviations from HWE might be due to the inclusion of multiple clonal copies of the same genotypes within populations, the (over-)interpreted summary of the results remained completely unchanged in the paper. For example, this part:

"Heterozygote excess in Ag_CA, Ag_CP, and Ar_PE were likely the result of heterosis or over-dominance related to selection preference toward heterozygous combination [81], or fixation of heterozygous genotypes; and, correspondingly, negative FIS values also showed an increase in heterozygosity that was generally due to random mating or outbreeding [82]. In contrast, heterozygote deficit (i.e., homozygote excess) in Ag_CJ, Ar_YO, Ar_PH, and Ar_RU was likely caused by retaining numerous unique genotypes

with private alleles within a population, and positive FIS values explained that the amount of heterozygous offspring in the population decreased, usually due to inbreeding [82]."

This is scientifically unsound in several respects. Without other, independent evidence it is simply not possible to infer heterosis as the cause of heterozygote excess at neutral markers.
Random mating will restore HWE and not lead to "an increase in heterozygosity". Etc.
>>
[Response and rebuttal]

Because aphids 'parthenogenetically' reproduce in their secondary host, especially under anholocyclic life, the result of heterosis or over-dominance related to selection preference toward heterozygous combination or fixation of heterozygous genotypes have been commonly reported in study of aphids. This is far from the normal reproductive situation under random mating on other diploid organisms. We cited additional references. Please see citations below:
81.Delmotte F, Sabater-Munoz B, Prunier-Leterme N, Latorre A, Sunnucks P, Rispe C, et al. Phylogenetic evidence for hybrid origins of asexual lineages in an aphid species. Evolution. 2003;57(6):1291-303. doi: Doi 10.1554/02-557. PubMed PMID: WOS:000183997400007.
82.Simon JC, Baumann S, Sunnucks P, Hebert PDN, Pierre JS, Le Gallic JF, et al. Reproductive mode and population genetic structure of the cereal aphid Sitobion avenae studied using phenotypic and microsatellite markers. Molecular Ecology. 1999;8(4):531-45. doi: DOI 10.1046/j.1365-294x.1999.00583.x. PubMed PMID: WOS:000080177700003.
83.Delmotte F, Leterme N, Gauthier JP, Rispe C, Simon JC. Genetic architecture of sexual and asexual populations of the aphid Rhopalosiphum padi based on allozyme and microsatellite markers. Molecular Ecology. 2002;11(4):711-23. doi: DOI 10.1046/j.1365-294X.2002.01478.x. PubMed PMID: WOS:000175250300007.
84.Wilson ACC, Sunnucks P, Blackman RL, Hales DF. Microsatellite variation in cyclically parthenogenetic populations of Myzus persicae in south-eastern Australia. Heredity. 2002;88(4):258-66. doi: 10.1038/sj.hdy.6800037.
In the respects to the description of heterozygote deficit, in the contrary to parthenogenetic life, sexual lineages generally show the heterozygote deficits [81-84]. However, as you pointed out, I cannot connect it without confirming clear evidence of correlation between sexual lineages and ecology of HAPs sampled from our study, thus deleted it (explanation of heterozygote deficits. Finally, the sentences were amended as below:
"Heterozygote excess in Ag_CA, Ag_CP, and Ar_PE were likely the result of heterosis or over-dominance related to selection preference toward heterozygous combination or fixation of heterozygous genotypes due to parthenogenesis of aphids in secondary host, especially under anholocyclic (permanently asexual) life [81]. Similar to our results, this phenomenon was already reported from several aphid species such as Sitobion avenae, Myzus persicae and Rhopalosiphum padi having permanently or temporary asexual life, which showed the significant heterozygote excess [82-84]. Negative FIS values also showed an increase in heterozygosity that was generally due to random mating or outbreeding, whereas positive FIS values explained that the amount of heterozygous offspring in the population decreased, usually due to inbreeding [85]." -> LINE 459-466 revised

Unlike the reviewer's request, because we do not treat discussion of the heterozygote excess in Ag_CA, Ag_CP, and Ar_PE, we still remain these contents in the results section in view of consistency of manuscript content.

[3]
You might also consider:
Providing a citation and/or example species in support of the statement in lines 632-634.

Add the related references to support the statement:
"This is similar to the tendency found in most polyphagous aphids that the primary host is shared, but the secondary host is completely different [32, 86-89]."
32.Moran NA. The Evolution of Aphid Life-Cycles. Annual Review of Entomology.

Powered by Editorial Manager® and ProduXion Manager® from Aries Systems Corporation

1992;37:321-48. doi: DOI 10.1146/annurev.ento.37.1.321. PubMed PMID: WOS:A1992GY50200014.
86.Lozier JD, Roderick GK, Mills NJ. Genetic evidence from mitochondrial, nuclear, and endosymbiont markers for the evolution of host plant associated species in the aphid genus Hyalopterus (Hemiptera : Aphididae). Evolution. 2007;61(6):1353-67. doi: DOI 10.1111/j.1558-5646.2007.00110.x. PubMed PMID: WOS:000247125100009.
87.Margaritopoulos JT, Tsitsipis JA, Goudoudaki S, Blackman RL. Life cycle variation of Myzus persicae (Hemiptera: Aphididae) in Greece. Bulletin of Entomological Research. 2002;92(4):309-19. Epub 2002/08/23. doi: 10.1079/BER2002167 S0007485302000366 [pii]. PubMed PMID: 12191439.
88.Guldemond JA. Host Plant Relationships and Life-Cycles of the Aphid Genus Cryptomyzus. Entomologia Experimentalis Et Applicata. 1991;58(1):21-30. PubMed PMID: WOS:A1991EZ54000003.
89.Jousselin E, Genson G, Coeur d'Acier A. Evolutionary lability of a complex life cycle in the aphid genus Brachycaudus. BMC evolutionary biology. 2010;10(1):295. doi: 10.1186/1471-2148-10-295. PubMed PMID: 20920188; PubMed Central PMCID: PMCPMC2958166.

**Additional Information:**

| Question | Response |
|---|---|
| **Financial Disclosure**<br><br>Enter a financial disclosure statement that describes the sources of funding for the work included in this submission. Review the submission guidelines for detailed requirements. View published research articles from *PLOS ONE* for specific examples.<br><br>This statement is required for submission and **will appear in the published article** if the submission is accepted. Please make sure it is accurate.<br><br>**Unfunded studies**<br>Enter: *The author(s) received no specific funding for this work.*<br><br>**Funded studies**<br>Enter a statement with the following details:<br>• Initials of the authors who received each award<br>• Grant numbers awarded to each author<br>• The full name of each funder<br>• URL of each funder website<br>• Did the sponsors or funders play any role in the study design, data collection and analysis, decision to publish, or preparation of the manuscript?<br>• **NO** - Include this sentence at the end of your statement: *The funders had no role in study design, data collection and analysis, decision to publish, or preparation of the manuscript.*<br>• **YES** - Specify the role(s) played. | The Korea Environment Industry & Technology Institute (KEITI) through Exotic Invasive Species Management Program funded this study via a grant (2018002270005) awarded to HK. (www.keiti.re.kr) The Basic Science Research Program through the National Research Foundation of Korea (NRF) funded by the Ministry of Education funded this study via a grant (2018R1D1A3B07044298) awarded to HK. (www.nrf.re.kr) The funders had no role in study design, data collection and analysis, decision to publish, or preparation of the manuscript.<br>BTL Bio-Test Labor GmbH provided support via salary for TT, but did not have any additional role in the study design, data collection and analysis, decision to publish, or preparation of the manuscript. (www.biotestlab.de) The specific roles of these authors are articulated in the 'author contributions' section. |

| | |
|---|---|
| * typeset | |
| **Competing Interests**<br><br>Use the instructions below to enter a competing interest statement for this submission. On behalf of all authors, disclose any competing interests that could be perceived to bias this work—acknowledging all financial support and any other relevant financial or non-financial competing interests.<br><br>This statement **will appear in the published article** if the submission is accepted. Please make sure it is accurate. View published research articles from *PLOS ONE* for specific examples.<br><br>**NO authors have competing interests**<br><br>Enter: *The authors have declared that no competing interests exist.*<br><br>**Authors with competing interests**<br><br>Enter competing interest details beginning with this statement:<br><br>*I have read the journal's policy and the authors of this manuscript have the following competing interests: [insert competing interests here]*<br><br>* typeset | The authors have read the journal's policy and declare the following competing interests: TT is a paid employee of BTL Bio-Test Labor GmbH (www.biotestlab.de). There are no patents, products in development or marketed products associated with this research to declare. This does not alter our adherence to PLOS ONE policies on sharing data and materials. |
| **Ethics Statement**<br><br>Enter an ethics statement for this submission. This statement is required if the study involved:<br><br>• Human participants<br>• Human specimens or tissue<br>• Vertebrate animals or cephalopods<br>• Vertebrate embryos or tissues<br>• Field research<br><br>Write "N/A" if the submission does not require an ethics statement. | As all collections have not been carried out in restricted areas, national parks, etc. where permits are required, it is clearly stated that there is no content regarding collection permits. |

| | |
|---|---|
| General guidance is provided below. Consult the submission guidelines for detailed instructions. **Make sure that all information entered here is included in the Methods section of the manuscript.**<br><br>**Format for specific study types**<br><br>**Human Subject Research (involving human participants and/or tissue)**<br>• Give the name of the institutional review board or ethics committee that approved the study<br>• Include the approval number and/or a statement indicating approval of this research<br>• Indicate the form of consent obtained (written/oral) or the reason that consent was not obtained (e.g. the data were analyzed anonymously)<br><br>**Animal Research (involving vertebrate animals, embryos or tissues)**<br>• Provide the name of the Institutional Animal Care and Use Committee (IACUC) or other relevant ethics board that reviewed the study protocol, and indicate whether they approved this research or granted a formal waiver of ethical approval<br>• Include an approval number if one was obtained<br>• If the study involved *non-human primates*, add *additional details* about animal welfare and steps taken to ameliorate suffering<br>• If anesthesia, euthanasia, or any kind of animal sacrifice is part of the study, include briefly which substances and/or methods were applied<br><br>**Field Research**<br><br>Include the following details if this study involves the collection of plant, animal, or other materials from a natural setting:<br>• Field permit number<br>• Name of the institution or relevant body that granted permission | |
| **Data Availability**<br><br>Authors are required to make all data | Yes - all data are fully available without restriction |

*Powered by Editorial Manager® and ProduXion Manager® from Aries Systems Corporation*

| | |
|---|---|
| underlying the findings described fully available, without restriction, and from the time of publication. PLOS allows rare exceptions to address legal and ethical concerns. See the PLOS Data Policy and FAQ for detailed information.<br><br>A Data Availability Statement describing where the data can be found is required at submission. Your answers to this question constitute the Data Availability Statement and **will be published in the article**, if accepted.<br><br>**Important:** Stating 'data available on request from the author' is not sufficient. If your data are only available upon request, select 'No' for the first question and explain your exceptional situation in the text box.<br><br>Do the authors confirm that all data underlying the findings described in their manuscript are fully available without restriction? | |
| **Describe where the data may be found in full sentences. If you are copying our sample text, replace any instances of XXX with the appropriate details.**<br><br>• If the data are **held or will be held in a public repository**, include URLs, accession numbers or DOIs. If this information will only be available after acceptance, indicate this by ticking the box below. For example: *All XXX files are available from the XXX database (accession number(s) XXX, XXX.*).<br>• If the data are all contained **within the manuscript and/or Supporting Information files**, enter the following: *All relevant data are within the manuscript and its Supporting Information files.*<br>• If neither of these applies but you are able to provide **details of access elsewhere**, with or without limitations, please do so. For example:<br><br>*Data cannot be shared publicly because of [XXX]. Data are available from the* | All relevant data are within the manuscript and its Supporting Information files (input files included in ZIP file). |

| | |
|---|---|
| *XXX Institutional Data Access / Ethics Committee (contact via XXX) for researchers who meet the criteria for access to confidential data.*<br><br>*The data underlying the results presented in the study are available from (include the name of the third party and contact information or URL).*<br>• This text is appropriate if the data are owned by a third party and authors do not have permission to share the data.<br><br>* typeset | |
| Additional data availability information: | Tick here if the URLs/accession numbers/DOIs will be available only after acceptance of the manuscript for publication so that we can ensure their inclusion before publication.; Tick here if your circumstances are not covered by the questions above and you need the journal's help to make your data available. |

*Powered by Editorial Manager® and ProduXion Manager® from Aries Systems Corporation*

Dear Editor in PLoS One

I resubmit a manuscript entitled “Complex evolution in *Aphis gossypii* group (Hemiptera: Aphididae), evidence of primary host shift and hybridization between sympatric species” to ‘PLoS One’.

All authors carefully reviewed the reviewer's review content, and faithfully revised it to complete the 2nd manuscript. Thank you very much to the editor and the two anonymous reviewers.
In addition, please check the revised statements of 'Financial Disclosure' and 'Competing interests' that all authors confirmed as below:

| | |
|---|---|
| Financial Disclosure | The Korea Environment Industry & Technology Institute (KEITI) through Exotic Invasive Species Management Program funded this study via a grant (2018002270005) awarded to HK. (www.keiti.re.kr) The Basic Science Research Program through the National Research Foundation of Korea (NRF) funded by the Ministry of Education funded this study via a grant (2018R1D1A3B07044298) awarded to HK. (www.nrf.re.kr) The funders had no role in study design, data collection and analysis, decision to publish, or preparation of the manuscript.<br>BTL Bio-Test Labor GmbH provided support via salary for TT, but did not have any additional role in the study design, data collection and analysis, decision to publish, or preparation of the manuscript. (www.biotestlab.de) The specific roles of these authors are articulated in the ‘author contributions’ section. |
| Competing interests | The authors have read the journal's policy and declare the following competing interests: TT is a paid employee of BTL Bio-Test Labor GmbH (www.biotestlab.de). There are no patents, products in development or marketed products associated with this research to declare. This does not alter our adherence to PLOS ONE policies on sharing data and materials. |

For response to second review, please see the 'response to reviewer comments' file. Thank you.

Best wishes and stay healthy,

Hyojoong Kim, Ph.D.
Associate Professor

Animal Systematics Lab., Dept. of Biology
Kunsan National University
Daehangno 558, Gunsan, Jeonbuk
573-701 Rep. of Korea
Tel: +82-63-469-4586
Fax: +82-63-469-7421
E-mail: hkim@kunsan.ac.kr

Manuscript

Click here to access/download;Manuscript;!+Manuscript_Aphis_210102_PO

# Complex evolution in *Aphis gossypii* group (Hemiptera: Aphididae), evidence of primary host shift and hybridization between sympatric species

**Yerim Lee[1], Thomas Thieme[2], Hyojoong Kim[1*]**

[1] Animal Systematics Laboratory, Department of Biology, Kunsan National University, Gunsan, Republic of Korea

[2] BTL Bio-Test Labor GmbH Sagerheide, RG Phyto-Entomology, Gross Lüsewitz, Germany

* Corresponding author

E-mail: hkim@kunsan.ac.kr **(HK)**

# Abstract

Aphids provide a good model system to understand the ecological speciation concept, since the majority of the species are host-specific, and they spend their entire lifecycle on certain groups of host plants. Aphid species that apparently have wide host plant ranges have often turned out to be complexes of host-specialized biotypes. Here we investigated the various host-associated populations of the two recently diverged species, *Aphis gossypii* and *A. rhamnicola*, having multiple primary hosts, to understand ~~the~~ complex evolution with host-associated speciation. Using mitochondrial DNA marker and nine microsatellite loci, we reconstructed ~~the~~ haplotype network, and analyzed the genetic structure and relationships. Approximate Bayesian computation was also used to infer the ancestral primary host and host-associated divergence, which resulted in *Rhamnus* being the most ancestral host for *A. gossypii* and *A. rhamnicola*. As a result, *Aphis gossypii* and *A. rhamnicola* do not randomly use their primary and secondary host plants; rather, certain biotypes use only some secondary and specific primary hosts. Some biotypes are possibly in a diverging state through specialization to specific primary hosts. Our results also indicate that a new heteroecious race can commonly be derived from the heteroecious ancestor, showing strong evidence of ecological specialization through a primary host shift in both *A. gossypii* and *A. rhamnicola*. Interestingly, *A. gossypii* and *A. rhamnicola* shared *COI* haplotypes with each other, thus there is a possibility of introgression by hybridization between them by cross-sharing same primary hosts. Our results contribute to a new perspective in the study of aphid evolution by identifying complex evolutionary trends in the *gossypii* sensu lato complex.

**KEY WORDS: host alternation, loss of primary host, molecular ecology, population genetics, speciation**

# Introduction

Phytophagous insects are a group of tremendous diversity that covers a quarter of all known terrestrial biodiversity [1, 2]. It has long been a concern to identify the evolutionary force of their remarkable diversity [3]. In most cases, phytophagous lineages have a much higher diversity than their closely related non-phytophagous lineages [3, 4]. They have an intimate relationship with certain and non-random host plant groups [5]. These findings often lead to the assumption that the host plant relationship holds the key to diversification in phytophagous insects [5, 6]. In particular, many observations of host-specific races have provided crucial evidence to support these assumptions [2, 7, 8]. Walsh [9] first proposed an ecological speciation scenario to explain the formation of sympatric host-associated populations (HAPs). The basic scenario of ecological speciation is that the transition to new host plants provides opportunities to have novel ecological niches for phytophagous insects, and contribute to different host preferences and genetic isolation; and this subsequently resulted in speciation [10].

The ecological speciation concept has also been extensively applied to explain the process of aphid speciation [11-14]. Aphids provide a good model system, since the majority of species are host-specific, and they spend their entire lifecycles on certain groups of host plants [15, 16]. Over the past decades, numerous studies have focused on the host relationship as a major factor in their speciation [15]. As one of the decisive examples, aphid species that apparently have wide host plant ranges (i.e. polyphagous) have often turned out to be complexes of host-specialized biotypes [11, 12]. It is well known that the pea aphid, *Acyrthosiphon pisum* (Harris), the most well-studied aphid species, is a set of genetically well-distinguished biotypes linked with different legume species, which can be described as an

example of sympatric speciation [11, 17-19]. A similar pattern is also found in *Uroleucon* spp., which live on certain species or some closely related plants within Asteraceae [20]. These groups of aphids show typical diversification patterns on a narrow range of related host plants (within the same family) through a trade-off in host use, gradual reduction of gene flow, and genetic drift [11, 19]. In addition, these examples are only possible if there is a mass of communities between closely related plants, which are mainly reported when many species of host plants live in similar conditions, such as asters and legumes [11, 20, 21].

In contrast to these classic examples, several species exhibit extreme polyphagous behavior, which occurs on a wide variety of unrelated plant families [15, 22]. As one of the most representative polyphagous aphids, the cotton-melon aphid, *Aphis gossypii* Glover, is associated with about 900 plants belonging to 116 plant families, including more than 100 important crops worldwide [22, 23]. The lifecycle of *A. gossypii* is as highly variable as its wide distribution range [22, 24, 25]. It has long been described as permanently anholocyclic [26], which is why studies on host change and primary hosts have rarely been conducted. ~~First,~~ Kring (27) reported that this aphid can perform a holocycly in North America. Today we have to assume that *gossypii* occurs in North America, East Asia, and Europe in numerous lines, some of which have a permanent anholocyclic reproduction, while others also have a holocyclic generation cycle [28]. Those points aside, the most unusual feature of this species is that they use primary hosts belonging to various unrelated plant families (e.g. Malvaceae, Punicaceae, Rhamnaceae, Rubiaceae and Rutaceae) [22, 26]. This is particularly interesting in evolutionary terms, and makes *A. gossypii* a good model for understanding the evolutionary process associated with the primary hosts of heteroecious aphids.

Approximately 10 % of 5,000 aphid species exhibit ~~the~~ seasonal host alternation (i.e. heteroecy) between primary and secondary hosts, which mysteriously are comprised with a set

of phylogenetically unrelated host plants [22, 29, 30]. In addition, among all phytophagous insects, the complex life cycle completed by multiple generations is known to be limited to the aphids (Aphidoidea) [31, 32]. In particular, the success of species diversity and remarkable host plant relationship are believed to be attributed to multiple acquisition and the loss of heteroecy [33, 34]. Lifecycles of heteroecious aphids usually comprise migration from the primary host to the secondary host [35]. For one cycle, sexual and asexual reproductions occur accompanied by several morphological changes as the generation passes [35]. Most heteroecious aphids use a much narrower range of primary host (within the same family or genus), even if they have a wide range of secondary hosts [36]. These patterns are even observed to extend to the closely related aphid species. For example, eight *Hyperomyzus* species are known to have host alternation with the primary hosts within the genus *Ribes*, even though these species have wider relationships with various secondary hosts, such as Asteraceae and Scrophulariaceae [22].

There are different views about these contrasting host ranges of primary and secondary hosts. The first view suggested that primary host specialization is in evolutionary terms more favored, because a primary host not only provides nutrients, but also a mating place [37]. For heteroecious aphids, a primary host is a place where sexual reproduction takes place, as well as the overwintering eggs hatch, and the following fundatrix stage [35, 38]. However, on the secondary host, only asexual reproduction occurs before the migration for overwintering [35, 38]. Thus, having different primary hosts, rather than those with different secondary hosts, may have a greater significance in reproduction. In other words, a constraint of primary host is possibly linked to the mating success of a species [15, 37]. The second view hypothesized that host alternation is only a by-product of an evolutionary process that occurs due to the phylogenetic constraints of fundatrix to the primary host [32, 33]. This is the so-called fundatrix specialization hypothesis (Fig 1), which is based on the following assumptions: i) monoecy is

evolutionarily more favored than heteroecy, ii) primary hosts are more ancestral than secondary hosts, iii) the fundatrix is highly adaptive to the primary host, but maladaptive to the secondary host, and iv) secondary hosts are more labile, and more recently obtained than primary hosts [31, 32, 39]. Under this hypothesis, the loss of a primary host was described as escape, and specialization to a specific secondary host was believed to be the only evolutionary way [31-33, 39, 40].

**Fig 1. A hypothetical scenario of the fundatrix specialization.** Concept of speciation by loss of primary host from Moran [33].

Having multiple primary hosts in *A. gossypii* [22, 24] suggests the possibility of host-associated speciation. Indeed, previous population genetic studies have shown that *A. gossypii* is a complex of several genetically distinct biotypes associated with some plant families (e.g. Cucurbitaceae, Malvaceae, and Solanaceae) [12, 41]. However, these studies only targeted anholocyclic lineages of *A. gossypii* collected from certain crops, and only a few primary hosts (e.g. *Hibiscus syriacus* and *Punica granatum*) [12, 41]. They live on a much broader range of wild plants, and the primary hosts are also very diverse. Nevertheless, we know surprisingly little about the primary host-associated genetic structure in this species. In particular, there is no study on the genetic structure between primary and secondary HAPs of *A. gossypii*. Therefore, to better understand the evolutionary trends in *A. gossypii*, further genetic analyses encompassing wild HAPs are needed.

In addition, confirming the ancestral host plant is crucial to understanding the evolutionary process of aphids. Among several host plants used as the primary host of *A. gossypii*, the genus *Rhamnus* (incl. *Frangula*) in Rhamnaceae is the most strongly presumed to be a ancestral host of the *gossypii* sensu lato complex group [42, 43]. There are several reasons

why *Rhamnus* is regarded as a ancestral host. The first reason is that most species belonging to the *gossypii* group show congruent use of primary hosts in *Rhamnus* [44], while the second reason is the possibility that the *gossypii* complex group and *Rhamnus* chronologically co-evolved based on molecular dating and fossil record [44, 45]. Because of this, *Rhamnus* has been believed to be at the center of the host-associated evolution in the *gossypii* complex group. Nevertheless, the relationships between *Rhamnus* and other secondary HAPs have not yet been investigated.

This study aims to investigate the evolutionary trends of the two closely-related host-alternating species, *A. gossypii* and *A. rhamnicola*, based on population genetic analyses of various primary and secondary HAPs. *Aphis gossypii* shows a typical heteroecious holocyclic lifecycle in Korea, for which various perennials and woody plants are known to be used as primary hosts [22], even though several anholocyclic isolates have been found in the secondary hosts [12]. *Aphis rhamnicola* is a recently described cryptic species of *A. gossypii* that shares *Rhamnus* spp. as rimary hosts, but has a somewhat different range of secondary hosts [46]. We conducted population genetic analyses of the two species in a comprehensive set of populations from primary and secondary host plants that were mostly collected from South Korea (except for one population from the UK). We used two molecular approaches in this study. First, reconstructing the haplotype network based on *COI* barcode, we confirmed their speciation pattern and genetic relationships of the aphid HAPs specialized on various host plants. Second, using nine microsatellite loci, we analyzed the genetic structure to identify the relationships between the HAPs of the two species, and to clarify the host shifting or switching process between the primary and secondary hosts. We also inferred the most likely ancestral host for the primary HAPs, which were strongly suggested through the results of this study, by using approximate Bayesian computation methods.

# Materials and Methods

## Taxon Sampling and DNA extraction

As all collections have not been carried out in restricted areas, national parks, etc. where permits are required, it is clearly stated that there is no content regarding collection permits. To examine the genetic structure, diversity and host-associated evolution between primary and secondary HAPs, we used 578 individual aphid samples of 36 HAPs, selectively pooled from 116 different collections, within the two species, *A. gossypii* and *A. rhamnicola*, which were collected from 36 different host plants - perennial, annual, and biannual; woody and herbaceous - in 16 plant families (Table 1). For forthcoming analyses, primary hosts and secondary hosts were defined based on the following criteria: i) The obvious primary hosts are plants ~~that have collected~~ sexuparae and fundatrix morphs. In addition to the obvious primary host, we also considered plants meeting the following two conditions as primary hosts. ii) Plants previously recorded as primary hosts of *A. gossypii* with reference to Inaizumi [23, 25] and Blackman and Eastop [28] or iii) the case when the collecting time is early spring (April-May) or late autumn (October-November) based on the lifecycle of *A. gossypii* on the Korean Peninsula; However, even if these two conditions were met, annual or biennial plants were excluded from ~~the~~ primary host. ~~It was also not~~ considered as ~~the~~ primary host ~~if aphid collected in a~~ greenhouse. As a consequence, all remaining plants not falling under the above conditions were considered as the secondary hosts. In our study, the host-associated population (HAP) means a collective population pooled from several temporally and/or geographically different collections in the same plant species (Table S1). In *A. gossypii* with a large spectrum of host utilization, 25 HAPs were collected from its various primary and secondary hosts (Table S1). As *A. rhamnicola* was recently recorded found in *Rhamnus* spp., sharing and co-existing with

*A. gossypii* in *Rhanmnus* as a primary host [46], nine HAPs of *A. rhamnicola* were also collected from its various primary and secondary hosts (Table S1).

These collections were acquired from South Korea, except for those of *A. gossypii* from *Catalpa ovata* in the UK (Table S1). To avoid the chance of sampling individuals from the same parthenogenetic colony, each aphid was collected from a different host plant, or a different isolated colony. All of the fresh aphid specimens used for molecular analyses were collected and preserved in (95 or 99) % ethanol, and stored at -70 °C. Total genomic DNA was extracted from single individuals using a DNeasy® Blood & Tissue Kit (QIAGEN, Inc., Dusseldorf). To preserve voucher specimens from the DNA extracted samples, we used a non-destructive DNA extraction protocol [43]. The entire body of the aphid was left in the lysis buffer with protease K solution at 55 °C for 24 h, and the cleared cuticle dehydrated.

**Table 1. Summary statistics for microsatellite data from all aphid populations.** Number of multilocus genotypes (MLGs); genotypic diversity (GD); observed heterozygosity (Ho); expected heterozygosity (He); Hardy-Weinberg Equilibrium (HWE); gene diversity (HS); mean number of alleles (NA); allelic richness (RS). ns: non-significance in HWE ($P > 0.05$). *$P$ values for heterozygote deficit or heterozygote excess. ($P < 0.001$) [a]possibly other cryptic species A. [b]possibly other cryptic species B. [c]known as primary host [25, 28]. [d]Host type, P: perennial, A: annual, B: biennial or annual, W: woody, H: herbaceous. [e] HWE estimated excluding the clonal copies of MLGs [f]*FIS* multiple loci.

| Pop. ID | No. | Sorted lineage | Host plant | Host type[d] | MLGs | $N_A$ | $H_S$ | $R_S$ | *Ho* (±s.e.) | *He* (±s.e.) | HWE[e] | *FIS*[f] |
|---|---|---|---|---|---|---|---|---|---|---|---|---|
| Ag_IL | 10 | *Aphis gossypii* Group 2 | *Ilex cornuta* | P, W | 5 | 2.33 | 0.36 | 2.12 | 0.49 (0.15) | 0.37 (0.10) | ns | -0.35 |
| Ag_CU | 20 | *Aphis gossypii* Group 2 | *Cucumis sativus* | A, H | 16 | 3.11 | 0.43 | 2.43 | 0.49 (0.11) | 0.43 (0.08) | ns | -0.16 |
| Ag_CM | 30 | *Aphis gossypii* Group 2 | *Cucurbita moschata* | A, H | 22 | 4.56 | 0.46 | 2.80 | 0.46 (0.11) | 0.46 (0.08) | ns | 0.00 |
| Ag_KA | 8 | *Aphis gossypii* Group 2 | *Kalanchoe daigremontiana* | P, W | 6 | 2.56 | 0.50 | 2.48 | 0.68 (0.14) | 0.51 (0.08) | ns | -0.35 |
| Ag_SO | 20 | *Aphis gossypii* Group 2 | *Solanum melongena* | P, W | 10 | 4.56 | 0.50 | 3.04 | 0.53 (0.13) | 0.50 (0.11) | ns | -0.07 |
| Ag_CA | 25 | *Aphis gossypii* Group 2 | *Capsicum annuum* | P, W | 6 | 2.44 | 0.39 | 2.12 | 0.64 (0.16) | 0.40 (0.10) | *excess | -0.63 |
| Ag_CP | 5 | *Aphis gossypii* Group 2 | *Capsicum annuum* var. *angulosum* | P, W | 4 | 1.89 | 0.36 | 1.89 | 0.64 (0.16) | 0.39 (0.10) | *excess | -0.79 |
| Ag_PU | 27 | *Aphis gossypii* Group 1 | *Punica granatum*[c] | P, W | 25 | 5.56 | 0.56 | 3.41 | 0.57 (0.08) | 0.56 (0.08) | ns | -0.01 |
| Ag_EL | 10 | *Aphis gossypii* Group 1 | *Eleutherococcus senticosus* | P, W | 9 | 2.67 | 0.43 | 2.40 | 0.42 (0.11) | 0.43 (0.09) | ns | 0.03 |
| Ag_HI | 60 | *Aphis gossypii* Group 1 | *Hibiscus syriacus*[c] | P, W | 59 | 7.33 | 0.50 | 3.25 | 0.49 (0.09) | 0.50 (0.08) | ns | 0.03 |
| Ag_HR | 10 | *Aphis gossypii* Group 1 | *Hibiscus rosa-sinensis*[c] | P, W | 8 | 2.22 | 0.31 | 2.02 | 0.38 (0.12) | 0.31 (0.09) | ns | -0.23 |
| Ag_EU | 10 | *Aphis gossypii* Group 1 | *Euonymus trapococca* | P, W | 9 | 3.56 | 0.52 | 3.04 | 0.56 (0.11) | 0.52 (0.10) | ns | -0.07 |
| Ag_EJ | 20 | *Aphis gossypii* Group 1 | *Euonymus japonicus* | P, W | 17 | 4.56 | 0.52 | 3.20 | 0.58 (0.10) | 0.52 (0.09) | ns | -0.12 |
| Ag_CI | 20 | *Aphis gossypii* Group 1 | *Citrus unshiu* | P, W | 16 | 4.89 | 0.51 | 3.17 | 0.57 (0.10) | 0.52 (0.06) | ns | -0.11 |
| Ag_FO | 10 | *Aphis gossypii* Group 1 | *Forsythia koreana* | P, W | 10 | 3.44 | 0.47 | 2.86 | 0.50 (0.11) | 0.47 (0.10) | ns | -0.07 |
| Ag_CE | 20 | *Aphis gossypii* Group 1 | *Celastrus orbiculatus*[c] | P, W | 19 | 4.44 | 0.46 | 2.90 | 0.52 (0.11) | 0.47 (0.09) | ns | -0.13 |
| Ag_ER | 10 | *Aphis gossypii* Group 1 | *Erigeron annuus* | A, H | 8 | 2.78 | 0.37 | 2.31 | 0.46 (0.12) | 0.37 (0.09) | ns | -0.23 |
| Ag_SN | 8 | *Aphis gossypii* Group 1 | *Sonchus oleraceus* | B, H | 7 | 3.00 | 0.45 | 2.59 | 0.50 (0.11) | 0.45 (0.08) | ns | -0.12 |
| Ag_CO | 18 | *Aphis gossypii* Group 1 | *Cosmos bipinnatus* | A, H | 16 | 3.56 | 0.51 | 2.82 | 0.48 (0.10) | 0.50 (0.08) | ns | 0.05 |
| Ag_CL | 10 | *Aphis gossypii* Group 1 | *Clinopodium chinense* var. *parviflorum* | P, H | 4 | 1.67 | 0.20 | 1.55 | 0.28 (0.11) | 0.21 (0.08) | ns | -0.37 |
| Ag_CT | 10 | *Aphis gossypii* Group 1 | *Catalpa ovata*[c] | P, W | 8 | 1.78 | 0.30 | 1.70 | 0.38 (0.11) | 0.30 (0.08) | ns | -0.26 |
| Ag_CJ | 10 | *Aphis gossypii* Group 1 | *Callicarpa japonica* | P, W | 8 | 3.11 | 0.48 | 2.64 | 0.30 (0.10) | 0.47 (0.08) | *deficit | 0.38 |
| Ag_RH | 20 | *Aphis gossypii* Group 1 | *Rhamnus davurica*[c] | P, W | 20 | 8.56 | 0.70 | 4.62 | 0.79 (0.07) | 0.70 (0.07) | ns | -0.12 |
| Ar_SE | 10 | *Aphis rhamnicola* Group 1[a] | *Sedum kamtschaticum* | P, H | 8 | 2.67 | 0.43 | 2.38 | 0.43 (0.10) | 0.43 (0.07) | ns | -0.01 |
| Ar_PE | 10 | *Aphis rhamnicola* Group 1[a] | *Perilla frutescens* var. *frutescens* | A, H | 6 | 1.89 | 0.27 | 1.77 | 0.42 (0.15) | 0.28 (0.09) | *excess | -0.54 |
| Ar_YO | 11 | *Aphis rhamnicola* Group 3[b] | *Youngia sonchifolia* | B, H | 10 | 3.67 | 0.47 | 2.90 | 0.23 (0.07) | 0.46 (0.10) | *deficit | 0.51 |
| Ar_IX | 11 | *Aphis rhamnicola* Group 3[b] | *Ixeris strigosa* | P, H | 9 | 2.89 | 0.39 | 2.39 | 0.27 (0.08) | 0.38 (0.09) | ns | 0.30 |
| Ar_RH | 8 | *Aphis rhamnicola* Group 1 | *Rhamnus davurica*[c] | P, W | 8 | 3.44 | 0.55 | 3.02 | 0.56 (0.07) | 0.55 (0.04) | ns | -0.01 |
| Ar_CO | 30 | *Aphis rhamnicola* Group 1 | *Commelina communis* | A, H | 30 | 6.67 | 0.59 | 3.58 | 0.55 (0.09) | 0.59 (0.08) | ns | 0.07 |
| Ar_LE | 10 | *Aphis rhamnicola* Group 1 | *Leonurus japonicus* | B, H | 10 | 4.11 | 0.53 | 3.07 | 0.61 (0.10) | 0.53 (0.07) | ns | -0.16 |
| Ar_PH | 7 | *Aphis rhamnicola* Group 1 | *Phryma leptostachy* | P, H | 5 | 2.33 | 0.43 | 2.23 | 0.19 (0.08) | 0.41 (0.06) | *deficit | 0.55 |
| Ar_ST | 6 | *Aphis rhamnicola* Group 2 | *Stellaria media* | B, H | 5 | 1.89 | 0.29 | 1.83 | 0.39 (0.14) | 0.30 (0.09) | ns | -0.36 |
| Ar_LY | 10 | *Aphis rhamnicola* Group 2 | *Lysimachia coreana* | P, H | 10 | 3.56 | 0.49 | 2.92 | 0.49 (0.12) | 0.49 (0.10) | ns | 0.01 |
| Ar_CB | 24 | *Aphis rhamnicola* Group 2 | *Capsella bursa-pastoris* | B, H | 24 | 4.11 | 0.50 | 2.83 | 0.55 (0.13) | 0.50 (0.10) | ns | -0.10 |
| Ar_VE | 10 | *Aphis rhamnicola* Group 2 | *Veronica insularis* | P, H | 10 | 3.22 | 0.49 | 2.79 | 0.53 (0.14) | 0.49 (0.10) | ns | -0.10 |
| Ar_RU | 40 | *Aphis rhamnicola* Group 2 | *Rubia akane* | P, W | 32 | 5.56 | 0.49 | 2.87 | 0.36 (0.08) | 0.49 (0.07) | *deficit | 0.27 |

## Species lineage sorting

In some aphid groups, morphological identification can be ambiguous, due to the lack of conclusive morphological evidence. The *Aphis* group is one of the most typical groups with the above problem. As a complementary way to avoid misidentification, host plant relationships, morphologies, and molecular tools are widely used to identify aphids [43, 46-48]. Because our study aims to demonstrate intra-specific genetic relationships based on host plant associations, species lineage sorting is significant to prevent biases of the results. The two *Aphis* species, *A. gossypii* and *A. rhamnicola*, we study here are not only very similar in morphology, but also share several host plants due to the polyphagy. Although we performed species identification through morphology and host plant relationships as a first step and also tested DNA barcoding for all individuals collected on their shared host plants (e.g. *Capsella*, *Rhamnus*, and *Rubia*), we found that there were a lot of the haplotypes cross-shared between *A. gossypii* and *A. rhamnicola* (see Results). Therefore, instead of identifying the species with 36 HAPs, we applied the dominant assignment (white, green, blue, red, dark blue) of the genetic structure (*K* =3, 4, 5) by STRUCTURE as well as the PCoA results (see Results) to sort their lineags into five groups as *Aphis gossypii* Group 1, *A. g.* Group 2, *A. rhmanicola* Group 1, *A. r.* Group 2 and *A. r.* Group 3 (Table 1). Accordingly, '*Aphis gossypii*' and '*A. rhamincola*', which are mentioned later, are meant to include all group lineages containing the HAPs assigned by the results. Table S1 shows detailed information for lineage sorted samples used in DNA analyses.

## Haplotype analysis

A 658 bp of the partial 5' region of the *cytochrome c oxidase subunit I* gene (*COI*), namely *COI* DNA barcode [49], was amplified using the universal primer sets: LEP-F1 5'-

ATTCAACCAATCATAAAGATAT-3' and LEP-R1, 5'-TAAACTTCTGGATGTCCAAAAA-3'. A polymerase chain reaction (PCR) was performed with AccuPower® PCR Premix (Bioneer, Daejeon, Rep. of Korea) in 20 mL reaction mixtures under the following conditions: initial denaturation at 95 °C for 5 min; followed by 35 cycles at 94 °C for 30 s, an annealing temperature of 45.2 °C for 40 s, an extension at 72 °C for 45 s, and the final extension at 72 °C for 5 min. All PCR products were assessed using a 1.5 % agarose gel electrophoresis. Successfully amplified samples were purified using a QIAquick PCR purification kit (Qiagen, Inc.), and then immediately sequenced using an automated sequencer (ABI Prism 3730XL DNA Analyzer) at Bionics Inc. (Seoul, Korea). Both morphological identification, based on voucher specimens in the insect museum in Kunsan National University with descriptions of Blackman and Eastop (22), Lee and Kim (24), Heie (26), and molecular identification method using the *COI* DNA barcode region for comparison with the previous *COI* DNA barcode database, were used [43, 46, 50].

All sequences that were obtained for DNA barcoding were initially examined and assembled using CHROMAS 2.4.4 (Technelysium Pty Ltd., Tewantin, Qld, AU) and SEQMAN PRO ver. 7.1.0 (DNA Star, Inc., Madison, Wisconsin, USA). In this step, poor-quality sequences were discarded to avoid biases. The final dataset containing 187 sequences was aligned using MAFFT ver. 7 [51], an online utility. Some ambiguous front and back sequences were removed at this stage, resulting in sequences of 583 bp that were finally used for haplotype analysis. All sequences were deposited in GenBank (accession no. MT461429-MT461602). The *COI* haplotypes of *A. gossypii* complex were analyzed using DNASP ver. 6.12.03 [52]. A median-joining network (MJ) was built using NETWORK ver. 5.0.1.1 [53]. The MJ result was annotated with host plants or species, and then visually summarized in Fig 2.

## Microsatellite genotyping

In this study, all 578 individuals of four species were successfully genotyped using nine microsatellite loci (AGL1-2, AGL1-10, AGL1-11, AGL1-15, AGL1-16, AGL1-20, AGL1-21, AGL1-22, and AGL2-3b) previously isolated from the soybean aphid [54]. In the preliminary study, we had already checked the cross-species amplification test of these loci on *A. fabae*, *Hyalopterus pruni*, *Rhopalosiphum padi*, and *Schizaphis graminum*, as well as *A. gossypii* in the tribe Aphidini (data not shown). There were the previously developed loci from *A. gossypii* [55], but we used the nine loci developed from *A. glycines*, because we noticed that the polymorphism of the latter was higher than that of the former, which was advantageous to amplify the loci between different species. In the aphid group, several studies showed that microsatellite loci were available between related species within the aphid family as a utility of cross-species amplification [56, 57].

Microsatellite amplifications were performed using GeneAll® *Taq* DNA Polymerase Premix (GeneAll, Seoul, Korea) in 20 μL reaction mixtures containing 0.5 μM forward labeled with a fluorescent dye (6-FAM, HEX, or NED) & reverse primers, and 0.05 μg of DNA template. PCR was performed using a GS482 thermo-cycler (Gene Technologies, Essex), according to the following procedure: initial denaturation at 95 °C for 5 min, followed by 34 cycles of 95 °C for 30 s; annealing at 56 °C for 40 s; extension at 72 °C for 45 s, and a final extension at 72 °C for 5 min. PCR products were visualized by electrophoresis on a 1.5 % agarose gel with a low-range DNA ladder to check for positive amplifications. Automated fluorescent fragment analyses were performed on the ABI PRISM 377 Genetic Analyzer (Applied Biosystems), and allele sizes of PCR products were calibrated using the molecular size marker, ROX labeled-size standard (GenScan™ ROX 500, Applied Biosystems). Raw data on each fluorescent DNA product was analyzed using GeneMapper® version 4.0 (Applied

Biosystems).

## Microsatellite data analysis

We used GENALEX 6.503 [58] to identify multilocus genotypes (MLGs) among populations. The program FSTAT 2.9.3.2 [59] was used to estimate the mean number of alleles ($N_A$), gene diversity ($H_S$), and allelic richness ($R_S$). Observed ($H_O$) and expected heterozygosity ($H_E$) values among loci were estimated using GENEPOP 4.0.7 [60] among the population data (HAPs) sets. Levels of significance for Hardy–Weinberg equilibrium (HWE) and linkage disequilibrium tests were adjusted using the sequential Bonferroni correction for all tests involving multiple comparisons [61]. Deviations from HWE were tested for heterozygote deficiency or excess. Because the clonal copies of MLGs due to the parthenogenetic life cycle of aphids could affect and distort the estimation of HWE [62], we used a reduced data set containing only one copy of each MLG when estimating HWE. Several assumptions of HWE still can be violated, thereby these estimates are used only for descriptive purposes even although the clonal MLG copies were removed from data analysis [62]. MICRO-CHECKER [Van 63] was used to test for null alleles [64] and identify possible scoring errors, because of the large-allele dropout and stuttering.

We used ARLEQUIN 3.5.1.2 [65] for calculations of pairwise genetic differentiation ($F_{ST}$) values [66], in which populations were assigned by 36 HAPs of the two species. The statistical significance of each value was assessed by computing the pairwise comparison of the observed value in 100,000 permutations. Groupings based on three different cases, (1) *gossypii* vs *rhamnicola*, (2) perennial vs non-perennial host groups in *A. gossypii*, (3) perennial vs non-perennial host groups in *A. rhamnicola*, were tested independently with analysis of molecular variance [AMOVA; 67] in ARLEQUIN, with significance determined using the

nonparametric permutation approach described by Excoffier et al. [68].

To examine the genetic relationships between 578 individual samples of four species, principal coordinate analysis (PCoA), also in GENALEX [58], further explored population relationships using the microsatellite loci, making no *a priori* assumptions about population groupings. Codominant genotypic genetic distance was calculated to make tri-matrix of pairwise populations, and then each population plot was created with coordinates based on the first two axes.

The program STRUCTURE 2.3.3 [69] was used to test for the existence of population structuring among all samples, by estimating the number of distinct populations ($K$) present in the set of samples, using a Bayesian clustering approach. We assessed likelihoods for models with the number of clusters ranging $K$ = (1 to 15). The length of the initial burn-in period was set to 100,000 iterations, followed by a run of 1,000,000 Markov chain Monte Carlo (MCMC) repetitions, of which the analysis was replicated 10 times, to ensure convergence on parameters and likelihood values. Parameter sets of ancestry, allele frequency, and advanced models remained as defaults. Following the method of Evanno, Regnaut (70), we calculated $\Delta K$ based on the second-order rate of change in the log probability of data with respect to the number of population clusters from the STRUCTURE analysis. To determine the correct value of $K$, both the likelihood distribution being to plateau or decrease [69] and the peak value of the $\Delta K$ statistic of Evanno, Regnaut (70) was estimated. The single run at each $K$ yielding the highest likelihood of the data given the parameter values was used for plotting the distributions of individual membership coefficients (Q) with the program DISTRUCT [71].

We performed assignment tests using GENECLASS 2 [72], in which populations were assigned to 36 HAPs of the two species. For each individual of a population, the program calculates the probability of belonging to any other reference population, or of being a resident of the population where it was sampled. The sample with the highest probability of assignment

was considered the most likely source for the assigned genotype. In this study, we checked the mean assignment rate from 391 *A. gossypii* or 187 *A. rhamnicola* individuals into each population (source), to confirm the possible origin of each HAP. We used a Bayesian method of estimating population allele frequencies [73]. Monte Carlo re-sampling computation (100,000 simulated individuals) was used to infer the significance of assignments (alpha = 0.01).

## Approximate Bayesian computation analysis

To estimate the relative likelihood of the most likely ancestral HAP of *A. gossypii*, an approximate Bayesian computation (ABC) was performed for the microsatellite dataset as implemented in DIYABC version 1.0.4 [74]. DIYABC allows the comparison of complex scenarios involving bottlenecks, serial or independent introductions, and genetic admixture events in introduced populations [75]. The parameters for modeling scenarios are the times of split or admixture events, the stable effective population size, the effective number of founders in introduced populations, the duration of the bottleneck during colonization, and the rate of admixture [76]. The software generates a simulated data set used to estimate the posterior distribution of parameters, in order to select the most likelihood scenario [76]. DIYABC generates a simulated data set that is then used to select those most similar to the observed data set, and the so-called selected data set ($n_\delta$), which are finally used to estimate the posterior distribution of parameters [74]. Recently, this ABC software package has been widely used, such as for inferring the demographic history of populations and species [77, 78], and testing potential bottleneck events [79].

To infer the most likely ancestral primary host of *A. gossypii*, among the whole microsatellite dataset, we tested three different ABC analyses using the original or partial

dataset. We hypothesized the evolutionary scenarios following our results obtained from the *COI* haplotype network and two Bayesian analyses, STRUCTURE and GENECLASS2 (see "Results"). The previous studies have already revealed that *A. rhamnicola* was located in the more ancestral position within the phylogeny of the *A. gossypii* group [43, 44], which population was therefore set to the most ancestral position on the genealogy of the two ABC tests.

In the first analysis, based on the result of STRUCTURE ($K = 3$), we compared eight evolutionary scenarios (A1–A8) using a dataset that included 578 individuals from four population groups, which consisted of 75 individuals from the 'BLUE' group (Ar_SE, Ar_PE, Ar_IX, Ar_YO, Ar_CO, Ar_PH, Ar_RH, Ar_LE), 90 from the 'GREEN' group (Ar_ST, Ar_VE, Ar_LY, Ar_CB, Ar_RU), 30 from the 'MIXBW (BLUE+WHITE)' group (Ag_RH, Ag_CJ), and 361 from the 'WHITE' group (Ag-IL, Ag_CE, Ag_EU, Ag_EJ, Ag_PU, Ag_CU, Ag_CM, Ag_KA, Ag_EL, Ag_HI, Ag_HR, Ag_FO, Ag_CI, Ag_ER, Ag_SN, Ag_CO, Ag_SO, Ag_CA, Ag_CP, Ag_CL, Ag_CT) (Fig S1). Scenario A1 considered (1) GREEN originated from BLUE, (2) MIXBW subsequently originated from GREEN, and then (3) WHITE originated from MIXBW. Scenario A2 considered (1) WHITE originated from BLUE, (2) MIXBW subsequently originated from WHITE, and then (3) GREEN originated from MIXBW. Scenario A3 considered (1) BLUE originated from GREEN, (2) MIXBW subsequently originated from BLUE, and then (3) WHITE originated from MIXBW. Scenario A4 considered (1) WHITE originated from MIXBW, (2) BLUE subsequently originated from WHITE, and then (3) GREEN originated from BLUE. Scenario A5 considered (1) GREEN originated from MIXBW, (2) BLUE subsequently originated from GREEN, and then (3) WHITE originated from BLUE. Scenario A6 considered (1) GREEN originated from WHITE, (2) MIXBW subsequently originated from GREEN, and then (3) BLUE originated from MIXBW. Scenario A7 considered (1) BLUE originated from MIXBW, (2) GREEN subsequently originated from

BLUE, and then (3) WHITE originated from GREEN. Scenario A8 considered (1) BLUE originated from WHITE, (2) MIXBW subsequently originated from BLUE, and then (3) GREEN originated from MIXBW.

In the second analysis, based on the result of STRUCTURE ($K$ = 4), we compared six evolutionary scenarios (B1–B6) using a dataset that included 311 individuals from four population groups, which consisted of 75 individuals from the 'BLUE' group (Ar_CO, Ar_PH, Ar_RH, Ar_SE, Ar_PE, Ar_LE), 90 from the 'GREEN' group (Ar_ST, Ar_VE, Ar_LY, Ar_CB, Ar_RU), 60 from the 'RED' group (Ag_IL, Ag_CU, Ag_CA, Ag_CP), and 86 from the 'WHITE' group (Ag_CE, Ag_FO, Ag_ER, Ag_SN, Ag_CO, Ag_CL, Ag_CT) (Fig S2). Scenario B1 considered (1) GREEN originated from BLUE, (2) WHITE subsequently originated from GREEN, and then (3) RED originated from WHITE. Scenario B2 was basically similar to scenario B1, except for (1) WHITE originated from RED. Scenario B3 considered (1) WHITE originated from RED, (2) GREEN subsequently originated from WHITE, and then (3) BLUE originated from GREEN. Scenario B4 considered (1) GREEN originated from BLUE, (2) WHITE formerly originated from GREEN, and then (3) RED later originated from GREEN. Scenario B5 considered (1) GREEN originated from BLUE, (2) RED formerly originated from GREEN, and then (3) WHITE later originated from GREEN. Scenario B6 considered (1) WHITE originated from RED, (2) GREEN formerly originated from WHITE, and then (3) BLUE later originated from GREEN.

In the third analysis, based on the result of STRUCTURE ($K$ = 4), we compared six evolutionary scenarios (C1–C6) using a dataset including 391 individuals from four population groups, except for BLUE and GREEN groups in the first and second analysis, which consisted of 30 individuals from the 'MBW (BLUE+WHITE)' group (Ag_RH, Ag_CJ), 207 from the 'MRW (RED+WHITE)' group (Ag_EU, Ag_EJ, Ag_PU, Ag_SO, Ag_CM, Ag_EL, Ag_HI, Ag_HR, Ag_CI), 68 from the 'RED' group (Ag_IL, Ag_CU, Ag_KA, Ag_CA, Ag_CP), and

86 from the 'WHITE' group (Ag_CE, Ag_FO, Ag_ER, Ag_SN, Ag_CO, Ag_CL, Ag_CT) (Fig S3). Scenario C1 considered (1) MRW originated from MBW, (2) WHITE subsequently originated from MRW, and then (3) RED originated from WHITE. Scenario C2 was basically similar to scenario C1, except for (3) WHITE originated from RED. Scenario C3 considered (1) WHITE originated from MBW, (2) RED subsequently originated from WHITE, and then (3) MRW originated from RED. Scenario C4 was basically similar to scenario C3, except for (2) MRW subsequently originated from WHITE, and then (3) RED originated from MRW. Scenario C5 considered (1) RED originated from MBW, (2) WHITE subsequently originated from RED, and then (3) MRW originated from WHITE. Scenario C6 was basically similar to scenario C5, except for (3) WHITE originated from MRW.

We produced 1 000 000 simulated data sets for each scenario. We used a generalized stepwise model (GSM) as the mutational model for microsatellites, which assumes increases or reductions by single repeat units [74]. To identify the posterior probability (PP) of these three scenarios, the $n_\delta$ = 30 000 (1 %) simulated datasets closest to the pseudo-observed dataset were selected for the logistic regression, which is similar to the $n_\delta$ = 300 (0.01 %) ones for the direct approach [76]. The summary of statistics was calculated from the simulated and observed data for each of the tested scenarios, such as the mean number of alleles per locus ($A$), mean genetic diversity for each group and between group, genetic differentiation between pairwise groups ($F_{ST}$), classification index, shared alleles distance ($D_{AS}$), and Goldstein distance.

# Results

## Haplotype analysis

A total of 18 haplotypes were recognized from the 187 *COI* sequences of 36 host-associated populations of the two *Aphis* species (Fig 2). The most common haplotype was H9, followed by H2. Aphid samples from the three primary hosts: *Hibiscus*, *Rhamnus*, and *Rubia* were spread across these two major haplotypes (Fig 2A). All the samples from the remaining primary hosts (i.e. *Catalpa*, *Celastrua*, *Citrus*, *Euonymus*, and *Punica*) had H9 haplotype (Fig 2A). Unique haplotypes were mostly observed among the *Rubia* population (Fig 2A). *Cucurbita*, *Hibiscus*, *Punica*, *Rhamnus*, *Sedum*, and *Youngia* associated populations also had unique haplotypes. Among the secondary HAPs, samples from the *Capsella* were found in both H2 and H9 haplotypes (Fig 2A). Samples from the *Youngia* was also observed in H1, H2, and H3 haplotypes (Fig 2A). However, the populations associated with the majority of secondary hosts only had one haplotype. H1 consisted of samples from secondary hosts, such as *Ixeris*, *Leonurus*, *Perilla*, *Phryma*, and *Youngia*. To compare *COI* haplotype and microsatellite genotype results, we overlaid the five biotypes that were identified from STRUCTURE ($K$ = 5) on the haplotype network (Fig 2B). The result of haplotype analysis was highly discordant with the STRUCTURE results (see below). Among the five biotypes, red, blue, and green types were observed in both H2 and H9 haplotypes (Fig 2B). The majority of white type aphids belonged to H9, while blue and green types were mostly found in H2 (Fig 2B). Aphids with green type showed the most diverse haplotype diversity (Fig 2B). The haplotype H1 contained blue and dark blue types.

**Fig 2. (A) Haplotype network for *COI* DNA barcode dataset (583 bp) using NETWORK ver. 5.0.1.1 [80].** Pie chart distribution based on each HAP; **(B) Haplotype network for *COI* DNA barcode dataset (583 bp) using NETWORK.** Pie chart distribution based on each group of five lineages in the two species.

## Microsatellite data analysis

We successfully genotyped 578 aphid individuals of 36 HAPs of the two species using 9 microsatellite loci, and then found 463 non-clonal mLGs from all samples (Table 1). Generally, genetic diversity was high throughout the HAPs collected from woody perennials, which seemed to be regarded as the primary (overwintering) hosts. The mean number of alleles ($N_A$) and gene diversity ($H_S$) in *A. gossypii* host populations averaged (4.17 and 0.45), respectively, whereas *A. rhamnicola* populations averaged (4.98 and 0.48), respectively. Similarly, allelic richness ($R_S$) ($R_S$, mean ± s.d., 2.67 ± 0.67) in the *A. gossypii* populations was slightly lower than $R_S$ (2.79 ± 0.50) in those of *A. rhamnicola*. Surprisingly, among all HAPs, Ag_RH, Ag_HI, and Ar_CO had relatively very high $N_A$ at (8.56, 7.33, and 6.67), respectively, of which the $R_S$ values were also high at (4.62, 3.25, and 3.58), respectively. The expected heterozygosity ($H_E$) values in the *A. gossypii* populations ranged (0.21 to 0.70), whereas $H_E$ values in the *A. rhamnicola* populations ranged (0.30 to 0.59). In HWE, there were significant deviations in Ag_CA, Ag_CP, and Ar_PE by heterozygote excess, and in Ag_CJ, Ar_YO, Ar_PH, and Ar_RU by heterozygote deficit. Heterozygote excess in Ag_CA, Ag_CP, and Ar_PE were likely the result of heterosis or over-dominance related to selection preference toward heterozygous combination or fixation of heterozygous genotypes due to parthenogenesis of aphids in secondary host, especially under anholocyclic (permanently asexual) life [81]. Similar to our results, this phenomenon was already reported from several aphid species such as *Sitobion avenae, Myzus persicae* and *Rhopalosiphum padi* having permanently or temporary asexual life, which showed the significant heterozygote excess [82-84]. Negative *FIS* values also showed an increase in heterozygosity that was generally due to random mating or outbreeding, whereas positive *FIS* values explained that the amount of heterozygous offspring in the population decreased, usually due to inbreeding [85]. There was

no evidence of significant linkage disequilibrium or frequency of null alleles.

## Genetic differentiation between host-associated populations and AMOVA

We estimated pairwise genetic differentiation ($F_{ST}$) between 36 different HAPs of the two species (Table 2). The averaging pairwise $F_{ST}$ values among the HAPs of all, only *A. gossypii* (Ar_SE, Ar_PE, Ar_YO, and Ar_IX) and only *A. rhamnicola* were 0.329, 0.209 and 0.392, respectively. In *A. gossypii*, it appeared that the averaging pairwise $F_{ST}$ values among the different HAPs obtained from host plants within the same plant genus or family were relatively low, such as Cucurbitaceae (Ag_CU, Ag_CM; averaging pairwise $F_{ST}$ = 0.040), Solanaceae (Ag_SO, Ag_CA, Ag_CP; 0.029), *Euonymus* (Ag_EU, Ag_EJ; 0.016), and Asteraceae (Ag_ER, Ag_SN, Ag_CO; 0.025). Remarkably, Ag_PU (0.130), Ag_HI (0.152), Ag_FO (0.159), and Ag_RH (0.134), considered to be the primary host, showed relatively low average $F_{ST}$ values toward the other *A. gossypii* populations. In *A. rhamnicola*, Ar_LY, Ar_CB, and Ar_VE were genetically close (0.050) to each other, despite belonging to the same host family/genus or being locally similar. In addition, Ar_RH was close to Ar_CO (0.089). Between *A. gossypii* and *A. rhamnicola* populations, Ar_ST and Ag_HI showed the lowest $F_{ST}$ value (0.095).

Two cases to confirm the genetic variance between the preordained groups were analyzed using AMOVA implemented in ARLEQUIN [67]. In the case of the analysis grouped by case 1, percentages of the genetic variance (PV) ‘among groups’ and ‘among populations within groups’ were 14.59 % and 22.60 %, respectively, which shows that there is some grouping effect by host plants, even though the majority of genetic variation was found ‘among individuals within populations’ as approximately 63 % (Table 3). However, the genetic variance of about -1 ~ 0 % ‘among groups’ in the both analyses grouped by cases 2 and 3

suggests that there are no grouped structures according to their lives in the perennial or non-perennial hosts on both *A. gossypii* and *A. rhamnicola* (Table 3). Interestingly, PV of ‘among populations within groups’ in *A. rhamnicola* was about 20 % higher than that in *A. gossypii*, which means that the HAPs of *A. rhamnicola* is genetically differentiated further than those of *A. gossypii* (Table 3).

**Table 2. Pairwise $F_{ST}$ divergence between 36 HAPs of the two species, *A. gossypii* groups (Ag) and *A. rhamnicola* groups (Ar)**. Values are significantly different from zero at $P < 0.05$ unless indicated as bold and italic

| | Ag_IL | Ag_CU | Ag_CM | Ag_KA | Ag_SO | Ag_CA | Ag_CP | Ag_PU | Ag_EL | Ag_HI | Ag_HR | Ag_EU | Ag_EJ | Ag_CI | Ag_FO | Ag_CE | Ag_ER | Ag_SN | Ag_CO |
|---|---|---|---|---|---|---|---|---|---|---|---|---|---|---|---|---|---|---|---|
| Ag_CU | 0.230 | | | | | | | | | | | | | | | | | | |
| Ag_CM | 0.183 | 0.040 | | | | | | | | | | | | | | | | | |
| Ag_KA | 0.202 | 0.145 | 0.092 | | | | | | | | | | | | | | | | |
| Ag_SO | 0.217 | 0.147 | 0.101 | 0.106 | | | | | | | | | | | | | | | |
| Ag_CA | 0.339 | 0.223 | 0.206 | 0.199 | 0.066 | | | | | | | | | | | | | | |
| Ag_CP | 0.352 | 0.209 | 0.187 | 0.163 | ***0.049*** | ***-0.030*** | | | | | | | | | | | | | |
| Ag_PU | 0.194 | 0.196 | 0.140 | 0.128 | 0.117 | 0.234 | 0.219 | | | | | | | | | | | | |
| Ag_EL | 0.387 | 0.267 | 0.203 | 0.140 | 0.224 | 0.337 | 0.311 | 0.216 | | | | | | | | | | | |
| Ag_HI | 0.169 | 0.137 | 0.060 | 0.090 | 0.121 | 0.244 | 0.216 | 0.111 | 0.196 | | | | | | | | | | |
| Ag_HR | 0.350 | 0.293 | 0.255 | 0.291 | 0.329 | 0.453 | 0.486 | 0.202 | 0.384 | 0.223 | | | | | | | | | |
| Ag_EU | 0.227 | 0.206 | 0.096 | 0.128 | 0.120 | 0.252 | 0.227 | 0.060 | 0.165 | 0.061 | 0.305 | | | | | | | | |
| Ag_EJ | 0.173 | 0.178 | 0.090 | 0.141 | 0.094 | 0.231 | 0.211 | 0.046 | 0.216 | 0.048 | 0.258 | 0.016 | | | | | | | |
| Ag_CI | 0.254 | 0.240 | 0.160 | 0.132 | 0.116 | 0.219 | 0.204 | 0.061 | 0.206 | 0.144 | 0.327 | 0.067 | 0.093 | | | | | | |
| Ag_FO | 0.234 | 0.260 | 0.159 | 0.184 | 0.149 | 0.300 | 0.295 | ***0.017*** | 0.266 | 0.106 | 0.235 | 0.064 | 0.040 | 0.087 | | | | | |
| Ag_CE | 0.299 | 0.295 | 0.194 | 0.169 | 0.215 | 0.332 | 0.312 | 0.134 | 0.114 | 0.152 | 0.283 | 0.090 | 0.124 | 0.119 | 0.137 | | | | |
| Ag_ER | 0.329 | 0.327 | 0.225 | 0.269 | 0.178 | 0.339 | 0.351 | 0.066 | 0.369 | 0.151 | 0.389 | 0.093 | 0.065 | 0.121 | 0.084 | 0.240 | | | |
| Ag_SN | 0.214 | 0.246 | 0.158 | 0.202 | 0.160 | 0.300 | 0.289 | 0.036 | 0.294 | 0.106 | 0.307 | ***0.045*** | 0.027 | 0.094 | 0.039 | 0.169 | ***0.028*** | | |
| Ag_CO | 0.204 | 0.240 | 0.161 | 0.179 | 0.146 | 0.287 | 0.274 | 0.015 | 0.279 | 0.100 | 0.237 | 0.057 | 0.033 | 0.086 | ***-0.008*** | 0.162 | 0.040 | ***0.008*** | |
| Ag_CL | 0.563 | 0.499 | 0.404 | 0.391 | 0.422 | 0.516 | 0.584 | 0.331 | 0.193 | 0.362 | 0.577 | 0.310 | 0.361 | 0.290 | 0.408 | 0.147 | 0.531 | 0.457 | 0.398 |
| Ag_CT | 0.432 | 0.353 | 0.279 | 0.311 | 0.271 | 0.346 | 0.386 | 0.191 | 0.371 | 0.257 | 0.435 | 0.214 | 0.209 | 0.176 | 0.254 | 0.241 | 0.297 | 0.232 | 0.232 |
| Ag_CJ | 0.234 | 0.255 | 0.205 | 0.225 | 0.201 | 0.308 | 0.301 | 0.119 | 0.342 | 0.170 | 0.312 | 0.176 | 0.127 | 0.213 | 0.136 | 0.264 | 0.213 | 0.126 | 0.115 |
| Ag_RH | 0.209 | 0.200 | 0.144 | 0.120 | 0.111 | 0.215 | 0.185 | 0.037 | 0.181 | 0.121 | 0.188 | 0.063 | 0.072 | 0.085 | 0.045 | 0.128 | 0.107 | 0.085 | 0.058 |
| Ar_SE | 0.473 | 0.439 | 0.400 | 0.351 | 0.344 | 0.417 | 0.403 | 0.304 | 0.411 | 0.361 | 0.515 | 0.359 | 0.335 | 0.356 | 0.367 | 0.379 | 0.435 | 0.392 | 0.365 |
| Ar_PE | 0.626 | 0.572 | 0.546 | 0.549 | 0.524 | 0.591 | 0.625 | 0.478 | 0.587 | 0.492 | 0.659 | 0.533 | 0.507 | 0.522 | 0.572 | 0.547 | 0.623 | 0.587 | 0.530 |
| Ar_YO | 0.469 | 0.411 | 0.411 | 0.397 | 0.421 | 0.479 | 0.468 | 0.374 | 0.466 | 0.387 | 0.496 | 0.412 | 0.395 | 0.428 | 0.448 | 0.457 | 0.491 | 0.435 | 0.415 |
| Ar_IX | 0.502 | 0.436 | 0.424 | 0.429 | 0.446 | 0.509 | 0.516 | 0.400 | 0.507 | 0.397 | 0.534 | 0.437 | 0.415 | 0.458 | 0.486 | 0.484 | 0.526 | 0.473 | 0.444 |
| Ar_RH | 0.463 | 0.428 | 0.383 | 0.338 | 0.348 | 0.437 | 0.393 | 0.347 | 0.396 | 0.330 | 0.490 | 0.337 | 0.352 | 0.382 | 0.387 | 0.390 | 0.449 | 0.403 | 0.383 |
| Ar_CO | 0.444 | 0.410 | 0.387 | 0.357 | 0.355 | 0.419 | 0.389 | 0.362 | 0.388 | 0.370 | 0.452 | 0.353 | 0.368 | 0.382 | 0.389 | 0.394 | 0.429 | 0.397 | 0.388 |
| Ar_LE | 0.474 | 0.437 | 0.413 | 0.368 | 0.411 | 0.491 | 0.443 | 0.376 | 0.398 | 0.348 | 0.509 | 0.363 | 0.369 | 0.410 | 0.430 | 0.394 | 0.481 | 0.427 | 0.410 |
| Ar_PH | 0.487 | 0.443 | 0.416 | 0.394 | 0.389 | 0.484 | 0.475 | 0.358 | 0.462 | 0.339 | 0.545 | 0.382 | 0.362 | 0.411 | 0.431 | 0.414 | 0.491 | 0.428 | 0.389 |
| Ar_ST | 0.356 | 0.301 | 0.191 | 0.239 | 0.313 | 0.444 | 0.458 | 0.263 | 0.367 | 0.095 | 0.440 | 0.216 | 0.208 | 0.295 | 0.294 | 0.274 | 0.387 | 0.304 | 0.270 |
| Ar_LY | 0.451 | 0.392 | 0.347 | 0.311 | 0.365 | 0.437 | 0.407 | 0.351 | 0.397 | 0.265 | 0.483 | 0.328 | 0.320 | 0.384 | 0.397 | 0.382 | 0.450 | 0.405 | 0.382 |
| Ar_CB | 0.406 | 0.361 | 0.321 | 0.282 | 0.355 | 0.425 | 0.395 | 0.344 | 0.366 | 0.239 | 0.436 | 0.318 | 0.315 | 0.363 | 0.377 | 0.355 | 0.432 | 0.389 | 0.367 |
| Ar_VE | 0.442 | 0.375 | 0.335 | 0.295 | 0.360 | 0.448 | 0.414 | 0.341 | 0.379 | 0.252 | 0.469 | 0.328 | 0.312 | 0.378 | 0.386 | 0.371 | 0.436 | 0.389 | 0.372 |
| Ar_RU | 0.451 | 0.417 | 0.383 | 0.381 | 0.389 | 0.463 | 0.442 | 0.352 | 0.421 | 0.333 | 0.456 | 0.343 | 0.336 | 0.381 | 0.380 | 0.376 | 0.417 | 0.388 | 0.362 |

**Table 2. (continued)**

| | Ag_CL | Ag_CT | Ag_CJ | Ag_RH | Ar_SE | Ar_PE | Ar_YO | Ar_IX | Ar_RH | Ar_CO | Ar_LE | Ar_PH | Ar_ST | Ar_LY | Ar_CB | Ar_VE |
|---|---|---|---|---|---|---|---|---|---|---|---|---|---|---|---|---|
| Ag_CT | 0.502 | | | | | | | | | | | | | | | |
| Ag_CJ | 0.520 | 0.340 | | | | | | | | | | | | | | |
| Ag_RH | 0.291 | 0.182 | 0.130 | | | | | | | | | | | | | |
| Ar_SE | 0.563 | 0.508 | 0.413 | 0.248 | | | | | | | | | | | | |
| Ar_PE | 0.720 | 0.667 | 0.561 | 0.399 | 0.549 | | | | | | | | | | | |
| Ar_YO | 0.617 | 0.521 | 0.407 | 0.307 | 0.494 | 0.570 | | | | | | | | | | |
| Ar_IX | 0.657 | 0.559 | 0.449 | 0.338 | 0.529 | 0.617 | 0.187 | | | | | | | | | |
| Ar_RH | 0.562 | 0.508 | 0.377 | 0.200 | 0.387 | 0.532 | 0.438 | 0.480 | | | | | | | | |
| Ar_CO | 0.492 | 0.456 | 0.381 | 0.209 | 0.383 | 0.440 | 0.418 | 0.447 | 0.089 | | | | | | | |
| Ar_LE | 0.560 | 0.531 | 0.407 | 0.294 | 0.410 | 0.428 | 0.454 | 0.470 | 0.292 | 0.317 | | | | | | |
| Ar_PH | 0.628 | 0.575 | 0.339 | 0.291 | 0.462 | 0.521 | 0.484 | 0.532 | 0.320 | 0.331 | 0.340 | | | | | |
| Ar_ST | 0.596 | 0.507 | 0.321 | 0.233 | 0.501 | 0.662 | 0.500 | 0.520 | 0.400 | 0.413 | 0.377 | 0.422 | | | | |
| Ar_LY | 0.573 | 0.495 | 0.403 | 0.267 | 0.418 | 0.570 | 0.455 | 0.463 | 0.295 | 0.327 | 0.253 | 0.384 | 0.245 | | | |
| Ar_CB | 0.509 | 0.467 | 0.391 | 0.280 | 0.423 | 0.535 | 0.446 | 0.441 | 0.314 | 0.347 | 0.253 | 0.360 | 0.178 | ***0.000*** | | |
| Ar_VE | 0.569 | 0.504 | 0.388 | 0.269 | 0.412 | 0.570 | 0.450 | 0.460 | 0.299 | 0.342 | 0.243 | 0.370 | 0.206 | ***0.023*** | 0.027 | |
| Ar_RU | 0.503 | 0.484 | 0.380 | 0.303 | 0.453 | 0.528 | 0.452 | 0.479 | 0.383 | 0.407 | 0.363 | 0.358 | 0.323 | 0.343 | 0.329 | 0.308 |

**Table 3. Analysis of molecular variance (AMOVA) results for microsatellite data analysis of aphids grouped by three cases:** (1) *gossypii* vs *rhamnicola*, (2) perennial vs non-perennial host groups in *A. gossypii*, (3) perennial vs non-perennial host groups in *A. rhamnicola*. $F_{st}$: among groups, $F_{sc}$: among populations within groups, $F_{ct}$: within populations, V: variance components. PV: percentage of variation.

| | Among groups | | | Among populations within groups | | | Within populations | | |
|---|---|---|---|---|---|---|---|---|---|
| Case | $V_a$ | PV | *P* | $V_b$ | PV | *P* | $V_c$ | PV | *P* |
| 1 | 0.50 | 14.59 | <0.0001 | 0.77 | 22.60 | <0.0001 | 2.14 | 62.81 | <0.0001 |
| 2 | -0.01 | -0.55 | <0.0001 | 0.48 | 18.54 | <0.0001 | 2.13 | 82.01 | 0.4458 |
| 3 | -2.59 | -0.09 | 0.0001 | 1.47 | 41.55 | <0.0001 | 2.16 | 61.04 | 0.8602 |

## Genetic similarity, structure, and assignment

A plot of PCoA between 36 HAPs based on codominant genotypic genetic distances showed that the two species, *A. gossypii* and *A. rhamnicola*, were completely separated in each of the left, upper–right, and lower–right sides on the plot (Fig 3). Plots of *A. gossypii* populations being closely aggregated along the line of factor 1 means that they are genetically close to each other, whereas the plots of *A. rhamnicola* being relatively largely scattered show genetic isolations between them. Among all *A. gossypii* populations, Ag_HI and Ag_RH were relatively located near to the HAPs of *A. rhamnicola*, Ar_ST and Ar_SE, respectively. Plots of Ar_YO and Ar_IX, which had been taxonomically considered to *A. gossypii*, were closely located to each other, but distant from the majority group of *A. gossypii*.

**Fig 3. A plot of the principal coordinate analysis based on the first two factors for 578 individuals of the four *gossypii* group species.** Each color corresponds to that shown in the results of STRUCTURE when $K = 3$ (Fig 3); white –23 HAPs of *A. gossypii*; blue – *Rhamnus* group, 7 HAPs of *A. rhamnicola*; green – *Rubia* group, 6 HAPs of *A. rhamnicola*. First and second coordinate axes account for (26.13 and 11.90) %, respectively.

The genetic structure of 36 HAPs of the two species (*A. gossypii* and *A. rhamnicola*) for 578 individuals was analyzed by STRUCTURE 2.3.3 [69]. In all STRUCTURE analyses from $K$ = (1 to 15), the most likely number of clusters was $K = 4$, using the $\Delta K$ calculation according to the method of Evanno, Regnaut (70). Here, we show the structure results from $K$ = (2 to 5), in order to observe the change of genetic structure and assignment pattern according to the $K$ value (Fig 4). When $K = 2$, the (first) white cluster dominantly appeared to *A. gossypii* populations, except for Ag_RH with a large green assignment, while the (second) green cluster was largely distributed among populations of *A. rhamnicola*. When $K = 3$, the (first) white cluster also was dominant in *A. gossypii* HAPs, except for Ag_RH with large blue assignment

and Ag_Hi with small green and blue ones, the (third) blue cluster as the '*Rhamnus* group' prevalent in Ar_SE, Ar_PE, Ar_YO, Ar_IX, Ar_RH, Ar_CO, Ar_LE, Ar_PH and Ar_ST, and the (second) green cluster in the rest as the '*Rubia* group'. When $K = 4$, the genetic structure was basically similar to that at $K = 3$, except that the (fourth) red cluster was dominant in Ag_IL, Ag_CU, Ag_CM, Ag_KA, Ag_SO, Ag_CA, and Ag_CP, and partially appeared in Ag_EL, Ag_HI, Ag_HR, Ag_EU, Ag_EJ, and Ag_CI. When $K = 5$, the genetic structure was basically similar to that at $K = 4$, except that both Ar_YO and Ar_IX showed the (fifth) dark-blue cluster.

**Fig 4. Genetic structure of 36 HAPs of the two *gossypii* complex species (*A. gossypii* and *A. rhamnicola*) for 578 individuals performed by STRUCTURE 2.3.3 [69].** Results are shown for $K$ = (2 to 5). Pop ID. (top) corresponds to Table 1, and the scientific plant name of each HAP is shown (bottom).

The Bayesian assignment tests using GENECLASS 2 (Piry et al. 2004) were carried out to identify the HAP (as population) membership of 578 individuals from all the 36 HAPs. The result of the assignment test (Table S2) indicated the average probability with which individuals were assigned to the corresponding reference HAP (as population). The self-assignment probability values (SA) averaged (0.482 ± 0.106) (mean ± s.d.) in overall HAPs, (0.515 ± 0.103) in *A. gossypii*, and (0.427 ± 0.08) in *A. rhamnicola*. In *A. gossypii*, the mean assignment probability from 391 *A. gossypii* individuals into Ag_RH had the highest value (0.446, SA = 0.381), which was followed by the assignment value into each reference HAP of Ag_HI (0.219, SA = 0.478) and Ag_PU (0.214, SA = 0.458) (Fig 5). In *A. rhamnicola*, the mean assignment probability from 145 *A. rhamnicola* individuals into Ar_RU had the highest value (0.137, SA = 0.489), which was similar to the assignment rate into each reference HAP of Ar_CB (0.131, SA = 0.463) and Ag_LY (0.129, SA = 0.309) (Fig 6).

**Fig 5. Mean assignment rate (blue bar, values on left) from 391 *Aphis gossypii*** individuals into each population (x column), and self-assignment rate (orange line, values on right) of individuals of each population using GENECLASS 2 [72].

**Fig 6. Mean assignment rate (blue bar, values on left) from 187 *Aphis rhamnicola*** individuals into each population (x column), and self-assignment rate (orange line, values on right) of individuals of each population using GENECLASS 2 [72].

## Inferring a ancestral primary host to test hypothetical scenarios by ABC analysis

To propose the most likely 'ancestral host evolution' scenario followed by the hypothesis that most of the *A. gossypii* populations originated from two possible ancestral HAPs (e.g. Ag_RH, Ag_HI), which had diverged from *A. rhamnicola*, the ABC test was conducted. We tested four scenarios to determine which HAP is the most ancestral among all the HAPs in *A. gossypii* (see "M&M"). The generated results are presented as a logistic regression using DIYABC software, estimating the PP of each tested evolutionary scenario of the hypothesis for the selected simulated data ($n_\delta$) (Cornuet et al. 2008), which ranged between (8 000 (or 6 000) and 80 000 (or 60 000)) $n_\delta$.

In the result of the first analysis (Fig S4), scenario A1 obtained the highest PP ranging (0.664 ($n_\delta$ = 8000) to 0.697 ($n_\delta$ = 80 000)), with a 95 % CI of (0.601–0.727) and (0.677–0.716). Scenario A2 showed a PP (Table 4). As a result, scenario A1 appeared as the most robust hypothesis with the highest PP among the four scenarios tested, which suggests that, compared to the other remaining hosts, *Rhamnus* is the most ancestral host for *A. gossypii* and *A. rhamnicola*, respectively.

In the result of the second analysis (Fig S5), the scenario B2 was estimated more highly

than the other four scenarios (Table 4). As a result, although the direct approach estimated a slightly higher PP for scenario B1 (0.520 and 0.480) than for B2 (0.460 and 0.448) (Fig S5), the scenario B2 appeared as the the highest PP in the logistic regression. It is well supported that *A. rhamnicola* is the origin of *A. gossypii* (B1, B2), but is not conclusive whether the RED group in *A. gossypii* is diverged from the WHITE group, or vice versa.

In the result of the third analysis (Fig S6), scenario C4 obtained the highest PP (Table 4). As a result, although the direct approach estimated a slightly higher PP for scenario C6 (0.400 and 0.326) and C5 (0.140 and 0.234) than for C4 (0.180 and 0.198) (Fig S6), the scenario C4 appeared as the highest PP among the four scenarios tested in the logistic regression. This suggests that, within *A. gossypii*, the WHITE group is more ancestral than the MRW and RED groups, and then RED is originated from MRW, which hypothesizes that *Hibiscus* is the secondarily primary host, and can be still a refuge for the RED group.

**Table 4. Probabilities (with 95% confidence intervals in brackets) of the logistic regression for the scenarios in three different analyses inferred from DIYABC [76].** For each comparison, the selected scenario (bold entry in shaded cell) was the one with the highest probability value

| | Posterior probability of each historical scenario | | | | | |
|---|---|---|---|---|---|---|
| | First analysis (Scenario A#) | | Second analysis (Scenario B#) | | Third analysis (Scenario C#) | |
| No. | $n_\delta$ = 8000 | $n_\delta$ = 80 000 | $n_\delta$ = 8000 | $n_\delta$ = 80 000 | $n_\delta$ = 8000 | $n_\delta$ = 80 000 |
| 1 | **0.6640 [0.6010,0.7269]** | **0.6965 [0.6767,0.7162]** | 0.4480 [0.3899,0.5061] | 0.4335 [0.4142,0.4529] | 0.0029 [0.0001,0.0056] | 0.0019 [0.0013,0.0024] |
| 2 | 0.0072 [0.0032,0.0112] | 0.0081 [0.0069,0.0094] | **0.5207 [0.4624,0.5790]** | **0.5169 [0.4974,0.5365]** | 0.0008 [0.0000,0.0017] | 0.0018 [0.0013,0.0024] |
| 3 | 0.3131 [0.2511,0.3751] | 0.2899 [0.2702,0.3095] | 0.0001 [0.0000,0.0002] | 0.0005 [0.0003,0.0006] | 0.2510 [0.1258,0.3762] | 0.2017 [0.1686,0.2349] |
| 4 | 0.0009 [0.0000,0.0023] | 0.0007 [0.0005,0.0009] | 0.0164 [0.0065,0.0263] | 0.0264 [0.0224,0.0303] | **0.3609 [0.2288,0.4929]** | **0.4917 [0.4460,0.5375]** |
| 5 | 0.0000 [0.0000,0.0001] | 0.0001 [0.0001,0.0001] | 0.0146 [0.0056,0.0236] | 0.0226 [0.0192,0.0260] | 0.1011 [0.0469,0.1554] | 0.0963 [0.0786,0.1139] |
| 6 | 0.0124 [0.0000,0.0259] | 0.0034 [0.0024,0.0044] | 0.0002 [0.0000,0.0007] | 0.0001 [0.0001,0.0001] | 0.2834 [0.1663,0.4004] | 0.2066 [0.1742,0.2389] |
| 7 | 0.0005 [0.0000,0.0012] | 0.0007 [0.0005,0.0009] | N/A | N/A | N/A | N/A |
| 8 | 0.0020 [0.0000,0.0042] | 0.0007 [0.0005,0.0008] | N/A | N/A | N/A | N/A |

# Discussion

## Complex evolution in *Aphis gossypii*

Our results identify the genetic structure between the various primary and secondary HAPs of the two species, *A. gossypii* and *A. rhamnicola*, encompassing the ~~most various~~ aphid ~~samples~~ ~~from~~ wild host plants. Our population genetic analyses reveal that *A. gossypii* and *A. rhamnicola* are mainly split into three (red, white, blue) and the other three (dark-blue, blue, green) biotypes, respectively, based on the STRUCTURE result (Fig 4, $K$ = 5). The evolutionary trend of these aphids cannot be defined in any particular direction, and they show complex and various speciation tendencies. Here, we highlight major cases in these species.

One of the notable results is that some secondary HAPs seem to use a specific primary host (Fig 4, $K$ = 4). In other words, *A. gossypii* and *A. rhamnicola* do not promiscuously use their primary and secondary host plants; instead, certain biotypes use only some secondary and specific primary hosts. For example, secondary HAPs having green biotype (e.g. *Capsella*, *Lysimachia*, *Stellaria*, and *Veronica*) seem to use only *Rubia* as the primary host in our dataset. On the other hand, *Rhamanus* serves as the primary host for the secondary HAPs having blue biotypes (e.g. *Commellina*, *Leonurus*, *Perilla*, *Phryma*, and *Sedum*). These cases indicate that a group that apparently uses several primary hosts is actually a complex of groups using a specific primary host.

In contrast to the previous cases, the white and red biotypes were found to share some primary and secondary hosts (Fig 4, $K$ = 4). In particular, the white biotype has been extensively found in the most diverse primary hosts, such as *Callicarpa*, *Catalpa*, *Celastrus*, *Citrus*, *Eunonymus*, *Hibiscus*, and *Punica*. The red biotype occurs in *Citrus*, *Eunonymus japonica*, *Hibiscus*, *Ilex*, and *Punica*. However, some primary hosts were exclusively occupied by the

white (e.g. *Catalpa* and *Celastrus*,) or red type (e.g. *Ilex*), suggesting that these biotypes are possibly in a state of diverging through specialization to specific primary hosts. Interestingly, similar to the first case (i.e. blue and green types), the white and red types also tended to use specific secondary host groups, respectively. Except for a few secondary hosts (e.g. *Cucurbita* and *Solanum*), most of them represented only one biotype. For example, *Cucumis sativus* and *Capsicum annuum* were completely occupied by the red biotype. This is similar to the tendency found in most polyphagous aphids that the primary host is shared, but the secondary host is completely different [32, 86-89].

In the STRUCTURE results, the dark-blue biotype (Fig 4, $K$ = 5) represents the third case. The dark-blue biotype was represented only by two secondary hosts, *Ixeris* and *Youngia*, and was not found in any primary host. Thus, we assume that this case seems to be an ecologically isolated host race through the loss of a primary host. Although we did not confirm the lifecycle of this biotype in this study, there is a reference to *A. gossypii* inhabiting some Asteraceae plants in the previous study, even though those HAPs are identified to *A. rhamnicola* based on our results (Figs 3 and 4). Blackman and Eastop [22] found that populations producing eggs on the roots of *Ixeris*, including some Asteraceae plants in China identified as *A. gossypii*, may be other closely-related species.With large genetic differences from the main group of both *A. gossypii* and *A. rhamnicola* (Fig 4), they were possibly isolated to the secondary host directly from the ancestral primary HAP in *Rhamnus* by the host alternation, supporting the possibility of differentiation from their ancestral host race according to the loss of primary host [32]. Thus, the dark-blue biotype is likely to be an ecologically incipient species of *A. rhamnicola*, which has recently been derived by secondary host isolation.

Our results show strong evidence of ecological specialization through a primary host shift in both *A. gossypii* and *A. rhamnicola*. ABC analyses yielded the biotypes of the two

species that were formed by shifting from the shared resource, *Rhamnus*, to different primary hosts, respectively (Figs S4 and S5). In particular, the series of primary host transitions identified in *A. gossypii* seem to have played an important role in the formation of their biotypes. For heteroecious aphids, a distinct choice of the primary host means not only utilizing different resources, but also genetic isolation between populations. This is because at one and the same time, the primary host is a resource, and a mating place. Accordingly, primary host selection in aphids is closely linked to genetic structure. Interestingly, in these species, primary host transitions occur more commonly than expected. As a traditional notion, primary hosts in aphids have been considered to be very fixed, and to not be able to easily escape, due to a highly adapted fundatrix morph [32, 33]. In particular, the white biotype that appears in many *A. gossypii* uses a wide variety of taxonomically unrelated primary hosts, which show a variable relationship between primary and fundatrix (Fig 4). But the results of our ABC analyses identified that *A. gossypii* was firstly derived from the biotype associated with *Rhamnus* to a white biotype, and then *Hibiscus* associated biotype was derived (Fig S4). Thus, having multiple primary hosts is possibly a transitional step to shifting to another primary host. In fact, *Hibiscus* is a plant closely related to *Gossypium* (i.e. cotton), a representative secondary host of *A. gossypii*. Unfortunately, although *Gossypium* associated population was not included in this study, it can be inferred that there is a possibility that the transition of primary host through secondary host may have occurred. However, similar to our results, Carletto, Lombaert (12) also suggested the possibility that *Hibiscus* was a shared ancestral host from which the agricultural divergence originated. in light of its HAP being genetically shared with the other HAPs in agricultural crops, such as *Gossypium*, cucurbits, and other secondary hosts.

Since the fundatrix specialization hypothesis [32, 33] has been proposed, the complex lifecycle of aphids has long been regarded as a by-product of aphid evolution. However, the identification of several heteroecious HAPs in *A. gossypii* and *A. rhamnicola* in our study is

largely in conflict with the expectations of this hypothesis. In our results, except for one case (i.e. the dark-blue biotype), the HAPs appear not to be genetically isolated completely but still to be linked together between some group of primary and secondary hosts, in contrast to the assumption that monoecy as a dead-end ~~is~~ evolutionarily favorable over heteroecy. Moran's hypothesis predicts that the dead-end of heteroecy always leads to specialization on the secondary host by loss of the primary host. Nevertheless, our results indicate that a new heteroecy race can commonly be derived from the heteroecy ancestors. In other words, our results show that lifecycle evolution is not a one-way process, but can be much more variable than we expected. These results are similar to the recent study on the genus *Brachycaudus* (Aphidinae: Macrosiphini), which provided strong evidence of the evolutionary lability of a complex lifecycle in *Brachycaudus* [89]. In addition, the use of several primary hosts found in some races (i.e. red and white biotypes) negates the core assumption of the fundatrix specialization hypothesis that the fundatrix is fully adapted to the ~~only~~ primary host, and is ~~inadequate~~ to other hosts. Using multiple primary hosts is possibly a strategy for their migration success. Indeed, a migration failure can lead to high risk. For example, aphids using only a single primary host, such as *Rhopalosiphum padi*, have only a 0.6 % migration success rate [90].

# Ancestral host association in *A. gossypii* complex

*Rhamnus* appears to be the most ancestral host plant for both *A. gossypii* and *A. rhamnicola*. Several species in the *gossypii* complex group have intimate relationships with *Rhamnus* (e.g. *A. frangulae*, *A. glycines*, *A. gossypii*, *A. nasturtii* and *A. rhamnicola*), which have been considered *Rhamnus* as an ancestral host for this aphid group (Blackman and Eastop, 2020; Kim et al., 2010). Our ABC result is consistent with this assumption (Figs S4 and S5).

As in the previous study, *Rhamnus* appears to serve as a shared primary host for both *A. gossypii* and *A. rhamnicola*. In the GENECLASS2 analyses (Figs 5 and 6) as well, the assignment of most of the *gossypii* HAPs to *Rhamnus* was very high, corroborating that it was differentiated from the ancestral host, *Rhamnus*.

Despite the differentiation of aphids into various species using the different hosts (mainly secondary hosts), host utilization of *Rhamnus* still remains in several species of the *gossypii* group. The phylogenetic studies of *Aphis* showed that heteroecious species using *Rhamnus* as the primary host were derived non-consecutively from monoecious species [44, 91]. In other words, even if a monoecious species has been derived by loss of heteroecy, it seems likely to not be ~~the dead-end of evolution~~ [33], as well as a complete disconnect from the ancestral primary host. For example, *A. rhamnicola* and *A. gossypii* are heteroecious species, which use *Rhamnus* as the primary host, and several monoecious species on various host plants appear to have been derived between them [46]. Our ABC analyses confirmed that *Rhamnus* was lost once when branching from blue type to green type, and was then regained in white type (Fig S4). Surprisingly, these ABC results, similarly supported by the GenClass2 results (Figs 5 and 6), almost coincided with our haplotype network results (Fig 2).

These results conflict the fundatrix specialization hypothesis [32, 33], which predicts that once aphids leave the ancestral primary host, they cannot regain it again. Recently, the phylogenetic study of *Brachycaudus* demonstrated that even if they lost their potential ancestral Rosaceae hosts, they can easily regain their hosts to be the primary host for heteroecy, or the sole host for monoecy [89]. The ancestral primary host does not seem to be an absolute being that cannot be changed due to the adaptation of the fundatrix, but seems to be a conserved resource within a specific aphid group. In fact, such a labile of aphid lifecycle related to the use of primary hosts may also occur within a species. Host alternation for some species is often

not obligatory but facultative, in which the migration to the secondary host can often be omitted [15, 92]. As an example, a facultative alternation lifecycle has been reported in populations of *Aphis fabae*, even although the vast majoriy of them migrate routinely between primary and secondary hosts [92]. Although there is little known about the facultative use of the primary host in *A. gossypii*, it may be related to the primary host range expansion and lifecycle lability.

## ~~The~~ evidence of hybridization between *A. gossypii* and *A. rhamnicola*

Our population genetic analyses based on microsatellite and *COI* gene show that there is a significant conflict between the two results. Regardless of the primary and secondary hosts, we found individuals that are difficult to identify in some host-associated populations. *A. gossypii* and *A. rhamnicola* appeared to share major haplotypes, H9 and H2, respectively, of their counterpart species with each other. Although the PCoA and STRUCTURE results (Figs 3 and 4) based on the microsatellites clearly showed identification of *A. gossypii*, the individuals corresponding to H2 (major haplotype of *A. rhamnicola*) were two individuals from Ag_Hi and one from Ag_KA, whereas the individuals corresponding to H9 (major haplotype of *A. gossypii*) were also identified as *A. rhamnicola*, but six from Ar_CB, one from Ar_PH ,and four from Ar_RU. Surprisingly, the cross-sharing haplotypes (H5, H9) between these two species unexpectedly contained several kinds of both primary and secondary hosts.

Comparing the host races between *A. gossypii* and *A. rhamnicola*, most of them were distributed in two haplotypes (H9 and H2), and they were clearly identified as distinct species, based on the microsatellite analysis (Fig 2). However, a number of intermediate haplotypes, H13, H11, H12, H10, and H18, were observed among the species (Fig 2). The H1 haplotype

shared by several wild plant populations, such as *Ixeris*, *Leonurus*, *Perilla*, *Phryma*, and *Youngia*, is closely related to the H9 (major haplotype of *A. gossypii*). However, according to our microsatellite data, these populations appear to be closer to *A. rhamnicola* (Figs 3 and 4). Similarly, collected from *Rubia akane*, individuals of H4, H6, H7, and H8 haplotypes apparently have alleles of *A. rhamnicola* in microsatellite data; it is most unusual that they have the haplotypes closely related to and derived from *A. gossypii*, rather than *A. rhamnicola*. In the case of H18, it is inferred to be the similar haplotype of true *A. gossypi* in Curcubitaceae, and H13 of Ar_SE, which was often cryptically recognized from *A. gossypii* as *A. rhamnicola* based on morphology and mtDNA [42, 93], was nested between the two species, *A. gossypii* and *A. rhamnicola*. Since it was reported that populations with a sexual phase on *Rubia cordifolia* in Japan appeared to be isolated from those on other primary hosts [25], it is suspected that most of the populations collected from *Rubia* in the past might actually be host races of *A. rhamnicola*, but misidentified.

Hybridization can have important evolutionary consequences, including speciation in association with novel host plants in insects [94]. In our study, as the two species, *A. gossypii* and *A. rhamnicola*, with distinct taxonomic and phylogenetic differences shared *COI* haplotypes of counterpart species with each other, there is a possibility of introgression by hybridization between them. These two species share overwintering (primary) host, such as *Rhamnus*, so mating and reproducing contemporarily in the same leaves or branches, because there are no physiological or ecological significant differences [43, 46]; there is therefore the possibility that hybridization occurs between them. In fact, interspecific cross mating between sexuparae of *A. glycines*, *A. gossypii*, and *A. rhamnicola* in *Rhamnus* spp. has often been observed (unpublished data). It is an interesting phenomenon that a hybrid zone mediated not by geography, but by a resource, can exist. Lozier et al. [87] first detected hybridization and introgression between plum and almond associated *Hyalopterus* spp. on these host plants,

which surprisingly were capable of feeding and developing on apricot from each species. For that possibility of hybridization, it was suggested that imperfections in any number of mechanisms associated with host plant choice [95, 96] could lead to strong selection against hybrids on parental host plants, but less so on apricot [87]. Although apricot was introduced later than other host species in the studied area, it remains a mystery why only it is able to attract all *Hyalopterus* groups and permit hybridization, whereas the other *Prunus* hosts are more restrictive [87]. Based on the phylogenetic results of *COI* or *Buchnera 16S* for *Hyalopterus* spp. [87], peach or apricot could be inferred to their ancestral host like the *Rhamnus* as a hybridization host utilized by the *gossypii* group. These results from the *gossypii* group reaffirm the hypothesis of Lozier et al. [87], which corroborates that such hybridization in the aphid group often occurs by co-existence in the primary host. However, further research is needed to determine whether a primary host (i.e. hosts that can be utilized by various host races, where they co-exist and overwinter together) are ancestral or derivational for those aphids.

# References


1. Chapman R. Foraging and food choice in phytophagous insects. In: Hardege JD, editor. Chemical ecology. Oxford: Eolss Publishers; 2009. p. 99-141.
2. Berlocher SH, Feder JL. Sympatric speciation in phytophagous insects: Moving beyond controversy? Annual Review of Entomology. 2002;47:773-815. PubMed PMID: ISI:000173421900025.
3. Jaenike J. Host Specialization in Phytophagous Insects. Annual Review of Ecology and Systematics. 1990;21(1):243-73. doi: 10.1146/annurev.es.21.110190.001331.
4. Mitter C, Farrell B, Wiegmann B. The phylogenetic study of adaptive zones: Has phytophagy promoted insect diversification? The American Naturalist. 1988;132(1):107-28.
5. Futuyma DJ, Agrawal AA. Macroevolution and the biological diversity of plants and herbivores. Proceedings of the National Academy of Sciences of the United States of America. 2009;106(43):18054-61. doi: DOI 10.1073/pnas.0904106106. PubMed PMID: ISI:000271222500006.
6. Mitter C, Farrell B, Futuyma D. Phylogenetic studies of insect-plant interactions: Insights into the genesis of diversity. Trends in Ecology and Evolution. 1991;6:290-3.
7. Ferrari J, Via S, Godfray HC. Population differentiation and genetic variation in performance on eight hosts in the pea aphid complex. Evolution. 2008;62(10):2508-24. doi: 10.1111/j.1558-5646.2008.00468.x. PubMed PMID: 18647340.
8. Lopez-Vaamonde C, Rasplus JY, Weiblen GD, Cook JM. Molecular phylogenies of fig wasps: Partial cocladogenesis of pollinators and parasites. Molecular Phylogenetics and Evolution. 2001;21(1):55-71. doi: DOI 10.1006/mpev.2001.0993. PubMed PMID: ISI:000171486700006.
9. Walsh BD. On phytophagic varieties and phytophagic species. Proc Entomol Soc Phila. 1864;3:403-30.
10. Matsubayashi KW, Ohshima I, Nosil P. Ecological speciation in phytophagous insects. Entomologia Experimentalis et Applicata. 2010;134(1):1-27. doi: DOI 10.1111/j.1570-7458.2009.00916.x. PubMed PMID: ISI:000272308300001.
11. Peccoud J, Ollivier A, Plantegenest M, Simon JC. A continuum of genetic divergence from sympatric host races to species in the pea aphid complex. Proceedings of the National Academy of Sciences of the United States of America. 2009;106(18):7495-500. PubMed PMID: ISI:000265783600043.
12. Carletto J, Lombaert E, Chavigny P, Brevault T, Lapchin L, Vanlerberghe-Masutti F. Ecological specialization of the aphid *Aphis gossypii* Glover on cultivated host plants. Molecular Ecology. 2009;18(10):2198-212. doi: DOI 10.1111/j.1365-294X.2009.04190.x. PubMed PMID: WOS:000265774300013.
13. Margaritopoulos JT, Malarky G, Tsitsipis JA, Blackman RL. Microsatellite DNA and behavioural studies provide evidence of host-mediated speciation in *Myzus persicae* (Hemiptera : Aphididae). Biological Journal of the Linnean Society. 2007;91(4):687-702. doi: DOI 10.1111/j.1095-8312.2007.00828.x. PubMed PMID: WOS:000248599900011.
14. Simon JC, Carre S, Boutin M, Prunier-Leterme N, Sabater-Muñoz, Latorre A, et al. Host-based divergence in populations of the pea aphid: insights from nuclear markers and the prevalence of facultative symbionts. Proceedings of the Royal Entomological Society of London (B). 2003;270:1703-12.
15. Peccoud J, Simon JC, von Dohlen C, Coeur d'acier A, Plantegenest M, Vanlerberghe-Masutti F, et al. Evolutionary history of aphid-plant associations and their role in aphid diversification. C R Biol. 2010;333(6-7):474-87. doi: 10.1016/j.crvi.2010.03.004. PubMed PMID: 20541159.
16. Kergoat GJ, Meseguer AS, Jousselin E. Chapter Two - Evolution of Plant–Insect Interactions: Insights From Macroevolutionary Approaches in Plants and Herbivorous Insects. In: Sauvion N, Thiéry D, Calatayud P-A, editors. Advances in Botanical Research. 81: Academic Press; 2017. p. 25-53.
17. Caillaud MC, Via S. Specialized feeding behavior influences both ecological specialization and assortative mating in sympatric host races of pea aphids. The American Naturalist. 2000;156(6):606-21. PubMed PMID: ISI:000165870000004.
18. Via S, Bouck AC, Skillman S. Reproductive isolation between divergent races of pea aphids on two hosts. II. Selection against migrants and hybrids in the parental environments. Evolution. 2000;54(5):1626-37. PubMed PMID: ISI:000165471000015.
19. Kanbe T, Akimoto SI. Allelic and genotypic diversity in long-term asexual populations of the pea aphid, *Acyrthosiphon pisum* in comparison with sexual populations. Molecular Ecology. 2009;18(5):801-16. doi: DOI 10.1111/j.1365-294X.2008.04077.x. PubMed PMID: WOS:000263521800006.
20. Moran NA, Kaplan ME, Gelsey MJ, Murphy TG, Scholes EA. Phylogenetics and evolution of the aphid genus *Uroleucon* based on mitochondrial and nuclear DNA sequences. Systematic Entomology. 1999;24(1):85-93. PubMed PMID: ISI:000078781800006.
21. Favret C, Voegtlin DJ. Speciation by host-switching in pinyon Cinara (Insecta: Hemiptera: Aphididae). Mol Phylogenet Evol. 2004;32(1):139-51. doi: 10.1016/j.ympev.2003.12.005. PubMed PMID: 15186803.

22. Blackman RL, Eastop VF. Aphids on the World's Plants. Available from: http://www.aphidsonworldsplant.info. 2019;(retrieval date 08/05/2019).
23. Inaizumi M. Studies on the life-cycle and polymorphism of *Aphis gossypii* Glover (Homoptera, Aphididae).) Special Bulletin of the College of Agriculture, Utsunomiya University. 1980;37(1-132).
24. Lee S, Kim H. Economic Insects of Korea 28 (Insecta Koreana Suppl. 35), Aphididae: Aphidini (Hemiptera: Sternorrhyncha). Suwon, Rep. of Korea: National Institue of Agricultural Science and Technology; 2006.
25. Inaizumi M. Life cycle of *Aphis gossypii* Glover (Homoptera, Aphididae) with special reference to biotype differentiation on various host plants. Kontyû. 1981;49:219-40.
26. Heie OE. The Aphidoidea (Hemiptera) of Fennoscandia and Denmark. III. Family Aphididae: subfamily Pterocommatinae & tribe Aphidini of subfamily Aphidinae. Klampenborg, Denmark: Scandinavian Science Press Ltd.; 1986.
27. Kring JB. The life cycle of the melon aphid, *Aphis gossypii* Glover, an example of facultative migration. Annals of the Entomological Society of America. 1959;52:284-6.
28. Blackman RL, Eastop VF. Aphids on the World's Herbaceous Plants and Shrubs, Vol. 2, The Aphids. Chichester: John Wiley & Sons Ltd.; 2006. 1025-439 p.
29. Eastop VF, Hille Ris Lambers D. Survey of the World's Aphids: Dr. W. Junk, The Hague (The Netherlands); 1976.
30. Favret C. Aphid Species File. Version 1.0/4.0. Available at: http://Aphid.SpeciesFile.org. Accessed 2019 Aug 6. 2019.
31. Moran NA. Adaptation and Constraint in the Complex Life-Cycles of Animals. Annual Review of Ecology and Systematics. 1994;25:573-600. doi: DOI 10.1146/annurev.ecolsys.25.1.573. PubMed PMID: WOS:A1994PU88300023.
32. Moran NA. The Evolution of Aphid Life-Cycles. Annual Review of Entomology. 1992;37:321-48. doi: DOI 10.1146/annurev.ento.37.1.321. PubMed PMID: WOS:A1992GY50200014.
33. Moran NA. The Evolution of Host-Plant Alternation in Aphids - Evidence for Specialization as a Dead End. Am Nat. 1988;132(5):681-706. doi: Doi 10.1086/284882. PubMed PMID: WOS:A1988R012000006.
34. von Dohlen CD, Rowe CA, Heie OE. A test of morphological hypotheses for tribal and subtribal relationships of Aphidinae (Insecta : Hemiptera : Aphididae) using DNA sequences. Molecular Phylogenetics and Evolution. 2006;38(2):316-29. doi: DOI 10.1016/j.ympev.2005.04.035. PubMed PMID: WOS:000235121800003.
35. Blackman RL, Eastop VF. Aphids on the World's Trees: An Identification and Information Guide. Wallingford: CAB International; 1994. 987 p.
36. Dixon AFG. The way of life of aphids: host specificity, speciation and distribution. In: Minks AK, Harrewijn P, editors. Aphids their biology, natural enemies and control, Vol A. A. Amsterdam: Elsevier; 1987. p. 197-207.
37. Ward SA. Reproduction and host selection by aphids: the importance of "rendezvous" hosts. In: Bailey WJ, Ridshill-Smith J, editors. Reproductve Behaviour of Insects. New york: Chapman and Hall; 1991. p. 202-26.
38. Blackman RL, Eastop VF. Aphids on the World's Crops: An Identification and Information Guide. 2nd ed. Chichester: John Wiley & Sons Ltd.; 2000. 466 p.
39. Hardy NB, Peterson DA, von Dohlen CD. The evolution of life cycle complexity in aphids: Ecological optimization or historical constraint? Evolution. 2015;69(6):1423-32. doi: 10.1111/evo.12643.
40. Moran NA. Aphid Life-Cycles - 2 Evolutionary Steps. Am Nat. 1990;136(1):135-8. doi: Doi 10.1086/285087. PubMed PMID: WOS:A1990DY11500009.
41. Charaabi K, Carletto J, Chavigny P, Marrakchi M, Makni M, Vanlerberghe-Masutti F. Genotypic diversity of the cotton-melon aphid *Aphis gossypii* (Glover) in Tunisia is structured by host plants. Bulletin of Entomological Research. 2008;98(4):333-41. doi: Doi 10.1017/S0007485307005585. PubMed PMID: WOS:000259233700002.
42. Cocuzza G, Cavalieri V, Barbagallos S. Preliminary results in the taxonomy of the cryptic group *Aphis frangulae*/*gossypii* obtained from mitochondrial DNA sequences. Bulletin of Insectology. 2008;61(1):125-6.
43. Kim H, Hoelmer KA, Lee W, Kwon YD, Lee S. Molecular and morphological identification of the soybean aphid and other *Aphis* species on the primary host *Rhamnus davurica* in Asia. Annals of the Entomological Society of America. 2010;103(4):532-43. doi: Doi 10.1603/An09166. PubMed PMID: ISI:000279548300008.
44. Kim H, Lee S, Jang Y. Macroevolutionary patterns in the Aphidini aphids (Hemiptera: Aphididae): diversification, host association, and biogeographic origins. PLoS One. 2011;6(9):e24749. doi: 10.1371/journal.pone.0024749. PubMed PMID: 21935453; PubMed Central PMCID: PMCPMC3174202.
45. Richardson JE, Chatrou LW, Mols JB, Erkens RHJ, Pirie MD. Historical biogeography of two cosmopolitan families of flowering plants: Annonaceae and Rhamnaceae. Philos T R Soc B. 2004;359(1450):1495-508. doi: DOI 10.1098/rstb.2004.1537. PubMed PMID: ISI:000224918700005.
46. Lee Y, Lee W, Lee S, Kim H. A cryptic species of *Aphis gossypii* (Hemiptera: Aphididae) complex revealed by genetic divergence and different host plant association. Bulletin of Entomological Research. 2015;105(1):40-51. Epub 11/21. doi: 10.1017/S0007485314000704.

47. Carletto J, Blin A, Vanlerberghe-Masutti F. DNA-based discrimination between the sibling species *Aphis gossypii* Glover and *Aphis frangulae* Kaltenbach. Systematic Entomology. 2009;34(2):307-14. PubMed PMID: ISI:000264374300008.
48. Foottit RG, Maw HEL, Havill NP, Ahern RG, Montgomery ME. DNA barcodes to identify species and explore diversity in the Adelgidae (Insecta: Hemiptera: Aphidoidea). Molecular Ecology Resources. 2009;9:188-95. PubMed PMID: ISI:000265227700019.
49. Hebert PDN, Cywinska A, Ball SL, DeWaard JR. Biological identifications through DNA barcodes. Proceedings of the Royal Society B-Biological Sciences. 2003;270(1512):313-21. doi: DOI 10.1098/rspb.2002.2218. PubMed PMID: WOS:000181064200013.
50. Lee W, Kim H, Lim J, Choi HR, Kim Y, Kim YS, et al. Barcoding aphids (Hemiptera: Aphididae) of the Korean Peninsula: updating the global data set. Mol Ecol Resour. 2011;11(1):32-7. doi: 10.1111/j.1755-0998.2010.02877.x. PubMed PMID: 21429098.
51. Katoh K, Rozewicki J, Yamada KD. MAFFT online service: multiple sequence alignment, interactive sequence choice and visualization. Briefings in Bioinformatics. 2017;20(4):1160-6. doi: 10.1093/bib/bbx108.
52. Rozas J, Ferrer-Mata A, Sánchez-DelBarrio JC, Guirao-Rico S, Librado P, Ramos-Onsins SE, et al. DnaSP 6: DNA Sequence Polymorphism Analysis of Large Data Sets. Molecular Biology and Evolution. 2017;34(12):3299-302. doi: 10.1093/molbev/msx248.
53. Bandelt H-J, Forster P, Röhl A. Median-joining networks for inferring intraspecific phylogenies. Molecular biology and evolution. 1999;16:37-48.
54. Andree K, Axtner J, Bagley MJ, Barlow EJ, Beebee TJC, Bennetzen JL, et al. Permanent Genetic Resources added to Molecular Ecology Resources Database 1 April 2010-31 May 2010. Molecular Ecology Resources. 2010;10(6):1098-105. doi: DOI 10.1111/j.1755-0998.2010.02898.x. PubMed PMID: ISI:000282876300023.
55. Vanlerberghe-Masutti F, Chavigny P, Fuller SJ. Characterization of microsatellite loci in the aphid species *Aphis gossypii* Glover. 1999.
56. Wilson ACC, Massonnet B, Simon JC, Prunier-Leterme N, Dolatti L, Llewellyn KS, et al. Cross-species amplification of microsatellite loci in aphids: assessment and application. Molecular Ecology Notes. 2004;4(1):104-9. doi: DOI 10.1046/j.1471-8286.2003.00584.x. PubMed PMID: WOS:000189159500032.
57. Michel AP, Zhang W, Jung JK, Kang ST, Mian MAR. Cross-Species Amplification and Polymorphism of Microsatellite Loci in the Soybean Aphid, Aphis glycines. J Econ Entomol. 2009;102(3):1389-92. PubMed PMID: ISI:000266641700068.
58. Peakall R, Smouse PE. GenAlEx 6.5: genetic analysis in Excel. Population genetic software for teaching and research--an update. Bioinformatics. 2012;28(19):2537-9. doi: 10.1093/bioinformatics/bts460. PubMed PMID: 22820204; PubMed Central PMCID: PMCPMC3463245.
59. Goudet J. FSTAT, A Program to Estimate and Test Gene Diversities and Fixation Indices (version 2.9.3.2). Institute of Ecology, University of Lausanne, Switzerland. Available from http//www.unil.ch/izea/softwares/fstat.html. 2002.
60. Raymond M, Rousset F. Genepop (version-1.2) - population genetics software for exact tests and ecumenicism. J Hered. 1995;86(3):248-9. PubMed PMID: ISI:A1995RB30200017.
61. Rice WR. Analyzing tables of statistical tests. Evolution. 1989;43(1):223-5. doi: Doi 10.2307/2409177. PubMed PMID: WOS:A1989R828900018.
62. Sunnucks P, DeBarro PJ, Lushai G, Maclean N, Hales D. Genetic structure of an aphid studied using microsatellites: Cyclic parthenogenesis, differentiated lineages and host specialization. Molecular Ecology. 1997;6(11):1059-73. doi: DOI 10.1046/j.1365-294X.1997.00280.x. PubMed PMID: WOS:A1997YJ88300006.
63. Oosterhout Cv, Hutchinson WF, Wills DPM, Shipley P. micro-checker: software for identifying and correcting genotyping errors in microsatellite data. Molecular Ecology Notes. 2004;4(3):535-8.
64. Brookfield JFY. A simple new method for estimating null allele frequency from heterozygote deficiency. Molecular Ecology. 1996;5(3):453-5. doi: 10.1046/j.1365-294X.1996.00098.x.
65. Excoffier L, Lischer HE. Arlequin suite ver 3.5: a new series of programs to perform population genetics analyses under Linux and Windows. Mol Ecol Resour. 2010;10(3):564-7. doi: 10.1111/j.1755-0998.2010.02847.x. PubMed PMID: 21565059.
66. Weir BS, Cockerham CC. Estimating F-statistics for the analysis of population structure. Evolution. 1984;38(6):1358-70. doi: Doi 10.2307/2408641. PubMed PMID: WOS:A1984TY40400017.
67. Excoffier L, Laval G, Schneider S. Arlequin (version 3.0): An integrated software package for population genetics data analysis. Evol Bioinform. 2005;1:47-50. PubMed PMID: ISI:000207065900004.
68. Excoffier L, Smouse PE, Quattro JM. Analysis of molecular variance inferred from metric distances among DNA haplotypes - application to human mitochondrial DNA restriction data. Genetics. 1992;131(2):479-91. PubMed PMID: WOS:A1992HW75900021.
69. Pritchard JK, Stephens M, Donnelly P. Inference of population structure using multilocus genotype data. Genetics. 2000;155(2):945-59. PubMed PMID: WOS:000087475100039.

70. Evanno G, Regnaut S, Goudet J. Detecting the number of clusters of individuals using the software STRUCTURE: A simulation study. Mol Ecol. 2005;14(8):2611-20. doi: DOI 10.1111/j.1365-294X.2005.02553.x. PubMed PMID: WOS:000229961500029.
71. Rosenberg NA. distruct: a program for the graphical display of population structure. Molecular Ecology Notes. 2004;4(1):137-8. doi: 10.1046/j.1471-8286.2003.00566.x.
72. Piry S, Alapetite A, Cornuet JM, Paetkau D, Baudouin L, Estoup A. GENECLASS2: A software for genetic assignment and first-generation migrant detection. J Hered. 2004;95(6):536-9. doi: DOI 10.1093/jhered/esh074. PubMed PMID: ISI:000224482500012.
73. Rannala B, Mountain JL. Detecting immigration by using multilocus genotypes. Proceedings of the National Academy of Sciences of the United States of America. 1997;94(17):9197-201. doi: DOI 10.1073/pnas.94.17.9197. PubMed PMID: WOS:A1997XR76500053.
74. Cornuet JM, Santos F, Beaumont MA, Robert CP, Marin JM, Balding DJ, et al. Inferring population history with DIY ABC: a user-friendly approach to approximate Bayesian computation. Bioinformatics. 2008;24(23):2713-9. doi: DOI 10.1093/bioinformatics/btn514. PubMed PMID: WOS:000261168600008.
75. Estoup A, Guillemaud T. Reconstructing routes of invasion using genetic data: why, how and so what? Mol Ecol. 2010;19(19):4113-30. doi: DOI 10.1111/j.1365-294X.2010.04773.x. PubMed PMID: WOS:000282180500005.
76. Cornuet JM, Ravigne V, Estoup A. Inference on population history and model checking using DNA sequence and microsatellite data with the software DIYABC (v1.0). Bmc Bioinformatics. 2010;11. doi: 10.1186/1471-2105-11-401. PubMed PMID: WOS:000281442400001.
77. Portnoy DS, Hollenbeck CM, Belcher CN, Driggers Iii WB, Frazier BS, Gelsleichter J, et al. Contemporary population structure and post-glacial genetic demography in a migratory marine species, the blacknose shark, Carcharhinus acronotus. Molecular Ecology. 2014;23(22):5480-95. doi: 10.1111/mec.12954.
78. Cabrera AA, Palsbøll PJ. Inferring past demographic changes from contemporary genetic data: A simulation-based evaluation of the ABC methods implemented in diyabc. Molecular Ecology Resources. 2017;17(6):e94-e110. doi: 10.1111/1755-0998.12696.
79. Techer MA, Clémencet J, Turpin P, Volbert N, Reynaud B, Delatte H. Genetic characterization of the honeybee (Apis mellifera) population of Rodrigues Island, based on microsatellite and mitochondrial DNA. Apidologie. 2015;46(4):445-54. doi: 10.1007/s13592-014-0335-9.
80. Bandelt HJ, Forster P, Röhl A. Median-joining networks for inferring intraspecific phylogenies. Molecular Biology and Evolution. 1999;16(1):37-48. doi: 10.1093/oxfordjournals.molbev.a026036.
81. Delmotte F, Sabater-Munoz B, Prunier-Leterme N, Latorre A, Sunnucks P, Rispe C, et al. Phylogenetic evidence for hybrid origins of asexual lineages in an aphid species. Evolution. 2003;57(6):1291-303. doi: Doi 10.1554/02-557. PubMed PMID: WOS:000183997400007.
82. Simon JC, Baumann S, Sunnucks P, Hebert PDN, Pierre JS, Le Gallic JF, et al. Reproductive mode and population genetic structure of the cereal aphid Sitobion avenae studied using phenotypic and microsatellite markers. Molecular Ecology. 1999;8(4):531-45. doi: DOI 10.1046/j.1365-294x.1999.00583.x. PubMed PMID: WOS:000080177700003.
83. Delmotte F, Leterme N, Gauthier JP, Rispe C, Simon JC. Genetic architecture of sexual and asexual populations of the aphid *Rhopalosiphum padi* based on allozyme and microsatellite markers. Molecular Ecology. 2002;11(4):711-23. doi: DOI 10.1046/j.1365-294X.2002.01478.x. PubMed PMID: WOS:000175250300007.
84. Wilson ACC, Sunnucks P, Blackman RL, Hales DF. Microsatellite variation in cyclically parthenogenetic populations of *Myzus persicae* in south-eastern Australia. Heredity. 2002;88(4):258-66. doi: 10.1038/sj.hdy.6800037.
85. Reichel K, Masson J-P, Malrieu F, Arnaud-Haond S, Stoeckel S. Rare sex or out of reach equilibrium? The dynamics of $F_{IS}$ in partially clonal organisms. BMC Genet. 2016;17(1):76-. doi: 10.1186/s12863-016-0388-z. PubMed PMID: 27286682.
86. Guldemond JA. Host Plant Relationships and Life-Cycles of the Aphid Genus *Cryptomyzus*. Entomologia Experimentalis Et Applicata. 1991;58(1):21-30. PubMed PMID: WOS:A1991EZ54000003.
87. Lozier JD, Roderick GK, Mills NJ. Genetic evidence from mitochondrial, nuclear, and endosymbiont markers for the evolution of host plant associated species in the aphid genus *Hyalopterus* (Hemiptera : Aphididae). Evolution. 2007;61(6):1353-67. doi: DOI 10.1111/j.1558-5646.2007.00110.x. PubMed PMID: WOS:000247125100009.
88. Margaritopoulos JT, Tsitsipis JA, Goudoudaki S, Blackman RL. Life cycle variation of *Myzus persicae* (Hemiptera: Aphididae) in Greece. Bulletin of Entomological Research. 2002;92(4):309-19. Epub 2002/08/23. doi: 10.1079/BER2002167
89. Jousselin E, Genson G, Coeur d'Acier A. Evolutionary lability of a complex life cycle in the aphid genus *Brachycaudus*. BMC evolutionary biology. 2010;10(1):295. doi: 10.1186/1471-2148-10-295. PubMed PMID: 20920188; PubMed Central PMCID: PMCPMC2958166.

90. Ward SA, Leather SR, Pickup J, Harrington R. Mortality during dispersal and the cost of host-specificity in parasites:how many aphids find hosts? Journal of Animal Ecology. 1998;67:763-73.
91. Coeur d'Acier A, Jousselin E, Martin JF, Rasplus JY. Phylogeny of the genus *Aphis* Linnaeus, 1758 (Homoptera : Aphididae) inferred from mitochondrial DNA sequences. Molecular Phylogenetics and Evolution. 2007;42(3):598-611. doi: DOI 10.1016/j.ympev.2006.10.006. PubMed PMID: WOS:000245717300003.
92. Mackenzie A. A trade-off for host plant utilization in the black bean aphid, *Aphis fabae*. Evolution. 1996;50(1):155-62. doi: Doi 10.2307/2410790. PubMed PMID: WOS:A1996TX89100016.
93. Lagos-Kutz DM, Favret C, Giordano R, Voegtlin D. Molecular and morphological differentiation between *Aphis gossypii* Glover (Hemiptera, Aphididae) and related species, with particular reference to the North American Midwest. ZooKeys. 2014;459:49-72.
94. Taylor SA, Larson EL. Insights from genomes into the evolutionary importance and prevalence of hybridization in nature. Nature Ecology & Evolution. 2019;3(2):170-7. doi: 10.1038/s41559-018-0777-y.
95. Dixon AFG. Aphid Ecology–An optimization approach. Norwell: Kluwer Academic Pub; 1998.
96. Powell G, Tosh CR, Hardie J. Host plant selection by aphids: behavioral, evolutionary, and applied perspectives. Annu Rev Entomol. 2006;51:309-30. doi: 10.1146/annurev.ento.51.110104.151107. PubMed PMID: 16332214.

# Supporting Information

**S1 Fig. The first eight scenarios (A1–A8) for the DIYABC analyses to infer the host evolution of the two *Aphis* species**, using a dataset that includes 578 individuals from four population groups, which consisted of 75 individuals from the 'BLUE' group (Ar_SE, Ar_PE, An_IX, An_YO, Ar_CO, Ar_PH, Ar_RH, Ar_LE); 90 from the 'GREEN' group (Ar_ST, Ar_VE, Ar_LY, Ar_CB, Ar_RU); 30 from the 'MIXBW (BLUE+WHITE)' group (Ag_RH, Ag_CJ); and 361 from the 'WHITE' group (Ag-IL, Ag_CE, Ag_EU, Ag_EJ, Ag_PU, Ag_CU, Ag_CM, Ag_KA, Ag_EL, Ag_HI, Ag_HR, Ag_FO, Ag_CI, Ag_ER, Ag_SN, Ag_CO, Ag_SO, Ag_CA, Ag_CP, Ag_CL, Ag_CT).

**S2 Fig. The second six scenarios (B1–B6) for the DIYABC analyses to infer the host evolution of the two *Aphis* species**, using a dataset that includes 311 individuals from four population groups, which consisted of 75 individuals from the 'BLUE' group (Ar_CO, Ar_PH, Ar_RH, Ar_SE, Ar_PE, Ar_LE); 90 from the 'GREEN' group (Ar_ST, Ar_VE, Ar_LY, Ar_CB, Ar_RU); 60 from the 'RED' group (Ag_IL, Ag_CU, Ag_CA, Ag_CP); and 86 from the 'WHITE' group (Ag_CE, Ag_FO, Ag_ER, Ag_SN, Ag_CO, Ag_CL, Ag_CT).

**S3 Fig. The third six scenarios (C1–C6) for the DIYABC analyses to infer the host evolution of *Aphis gossypii***, using a dataset that includes 391 individuals from four population groups except for BLUE and GREEN groups in the first and second analysis, which consisted of 30 individuals from the 'MBW (BLUE+WHITE)' group (Ag_RH, Ag_CJ); 207 from the 'MRW (RED+WHITE)' group (Ag_EU, Ag_EJ, Ag_PU, Ag_SO, Ag_CM, Ag_EL, Ag_HI, Ag_HR, Ag_CI); 68 from the 'RED' group (Ag-IL, Ag_CU, Ag_KA, Ag_CA, Ag_CP); and 86 from the 'WHITE' group (Ag_CE, Ag_FO, Ag_ER, Ag_SN, Ag_CO, Ag_CL, Ag_CT).

**S4 Fig. Plots output by DIYABC showing the PP (y-axis) of the first eight scenarios (A1–A8) through the direct estimate (left), and the logistic regression (right) approaches, as output by DIYABC.** The x-axis corresponds to the different $n_\delta$ values used in the computations. The results have been obtained by performing the first analysis with four scenarios.

**S5 Fig. Plots output by DIYABC showing the PP (y-axis) of the second six scenarios (B1–B6) through the direct estimate (left), and the logistic regression (right) approaches, as output by DIYABC.** The x-axis corresponds to the different $n_\delta$ values used in the computations. The results have been obtained by performing the first analysis with four scenarios.

**S6 Fig. Plots output by DIYABC showing the PP (y-axis) of the third six scenarios (C1–C6) through the direct estimate (left), and the logistic regression (right) approaches, as output by DIYABC.** The x-axis corresponds to the different $n_\delta$ values used in the computations. The results have been obtained by performing the first analysis with four scenarios.

**S1 Table. Collection data for 578 aphids analyzed in this study.** [†] possibly *A. rhamnicola* or other cryptic species [††] possibly other cryptic species

**S2 Table. Mean assignment rate of individuals into (rows) and from (columns) each population using GeneClass 2 (Piry et al. 2004).** Values in bold indicate the proportions of individuals assigned to the source population (self-assignment). Values less than 0.001 were excluded from the table

**S3 Table. Probabilities (with 95% confidence intervals in brackets) of the logistic regression for the scenarios in three different analyses inferred from DIYABC**. For each comparison, the selected scenario (bold entry in shaded cell) was the one with the highest probability value.

Figure 1

Click here to access/download;Figure;Fig1.eps

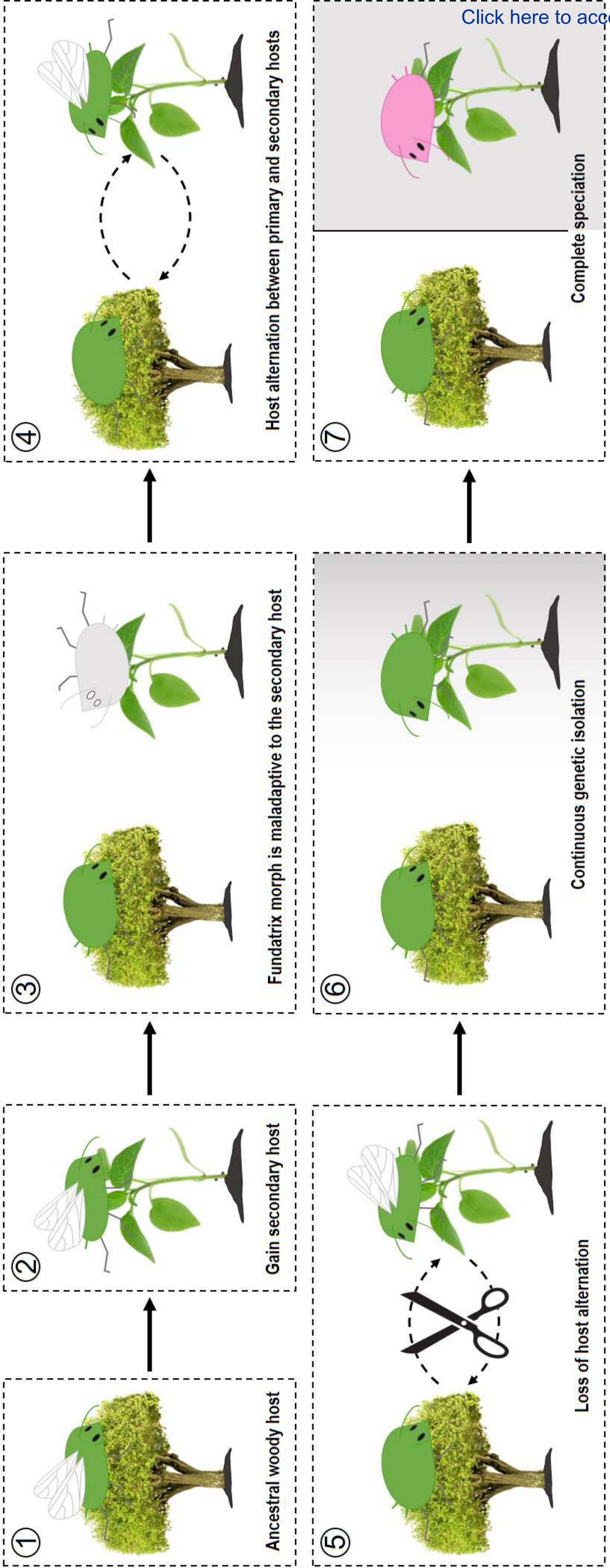

Figure 2

Click here to access/download;Figure;Fig2. revised.eps

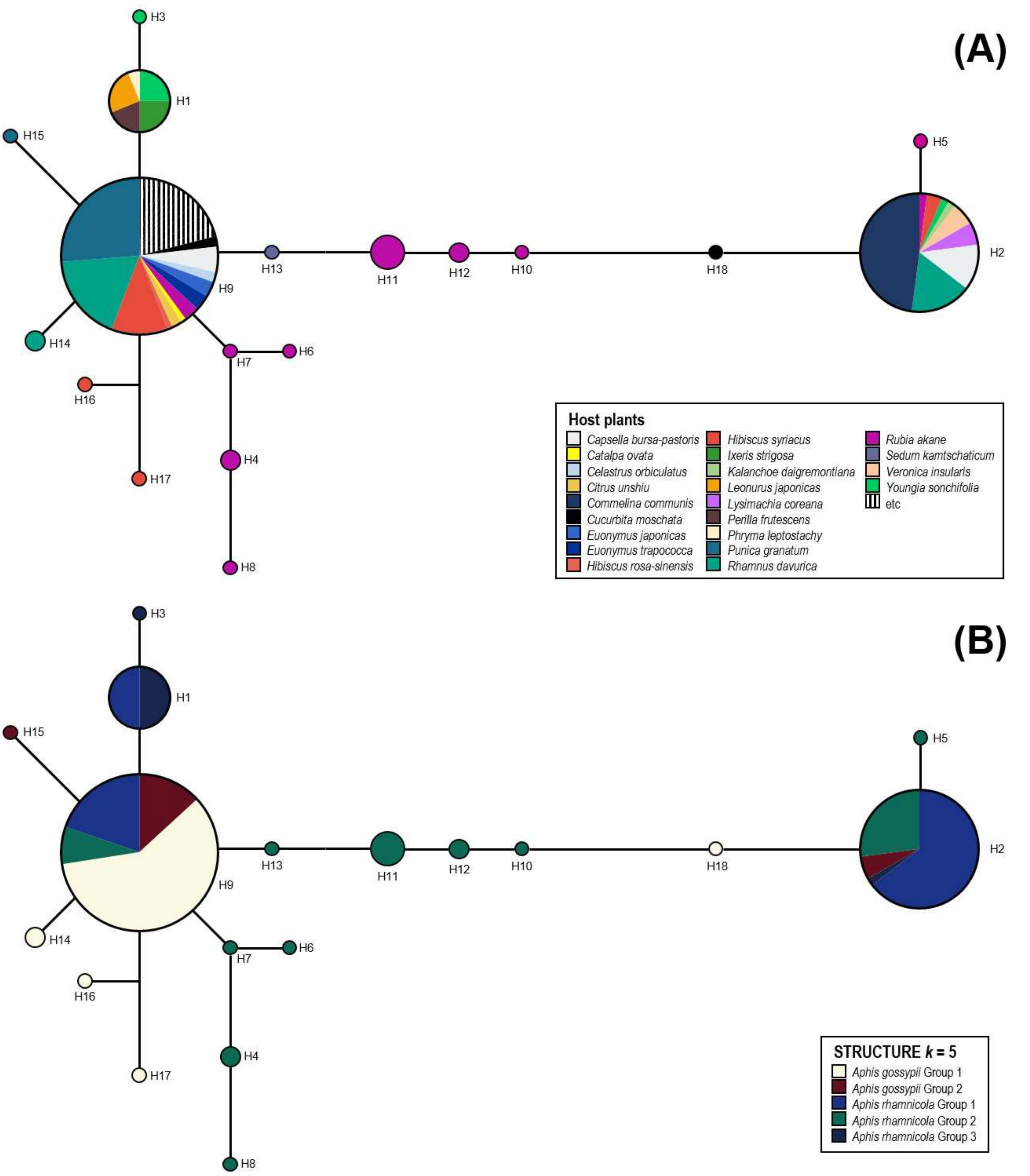

Figure 3

Click here to access/download;Figure;Fig3.tif

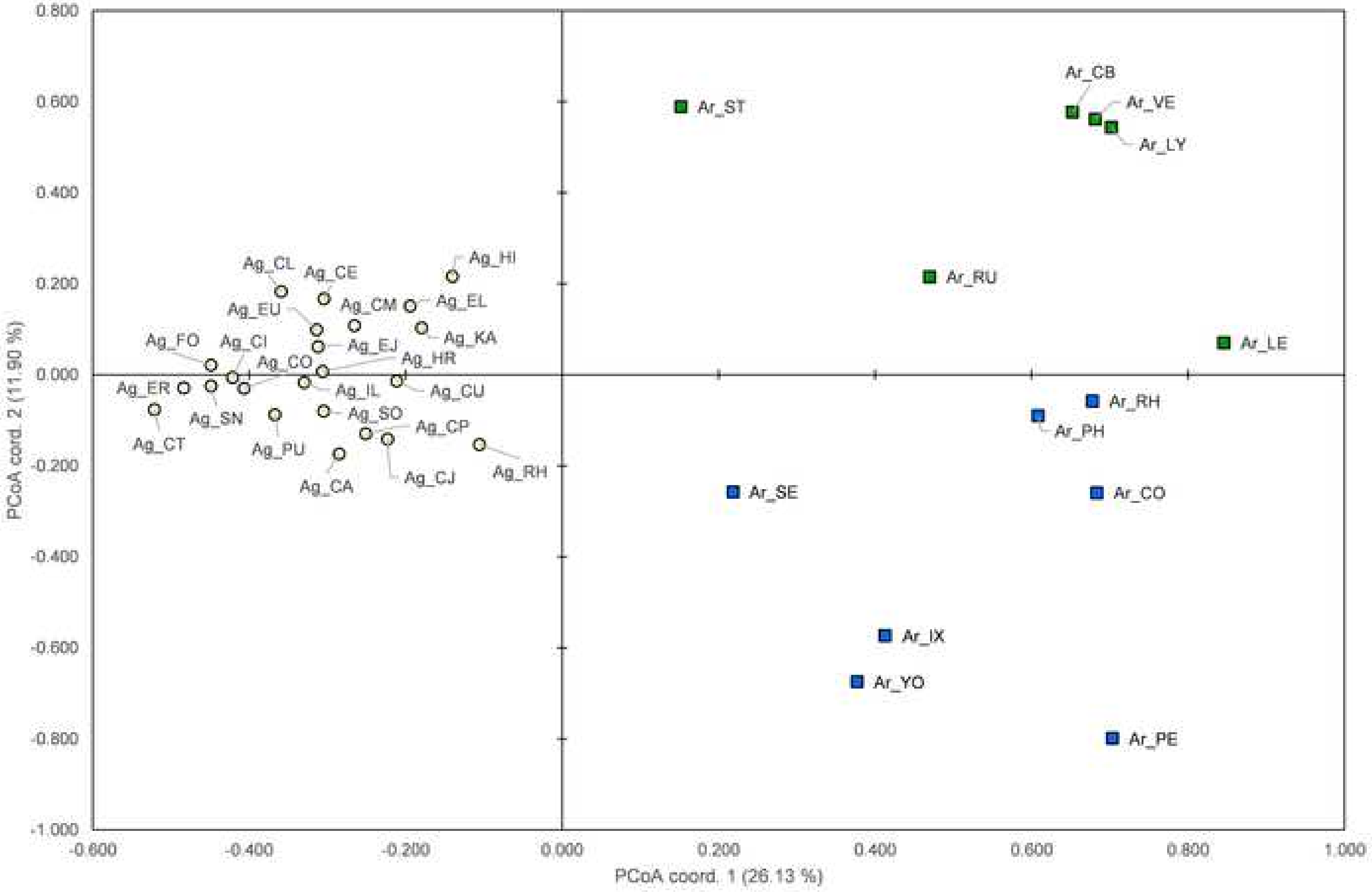

Click here to access/download;Figure;Fig4. revised.tif

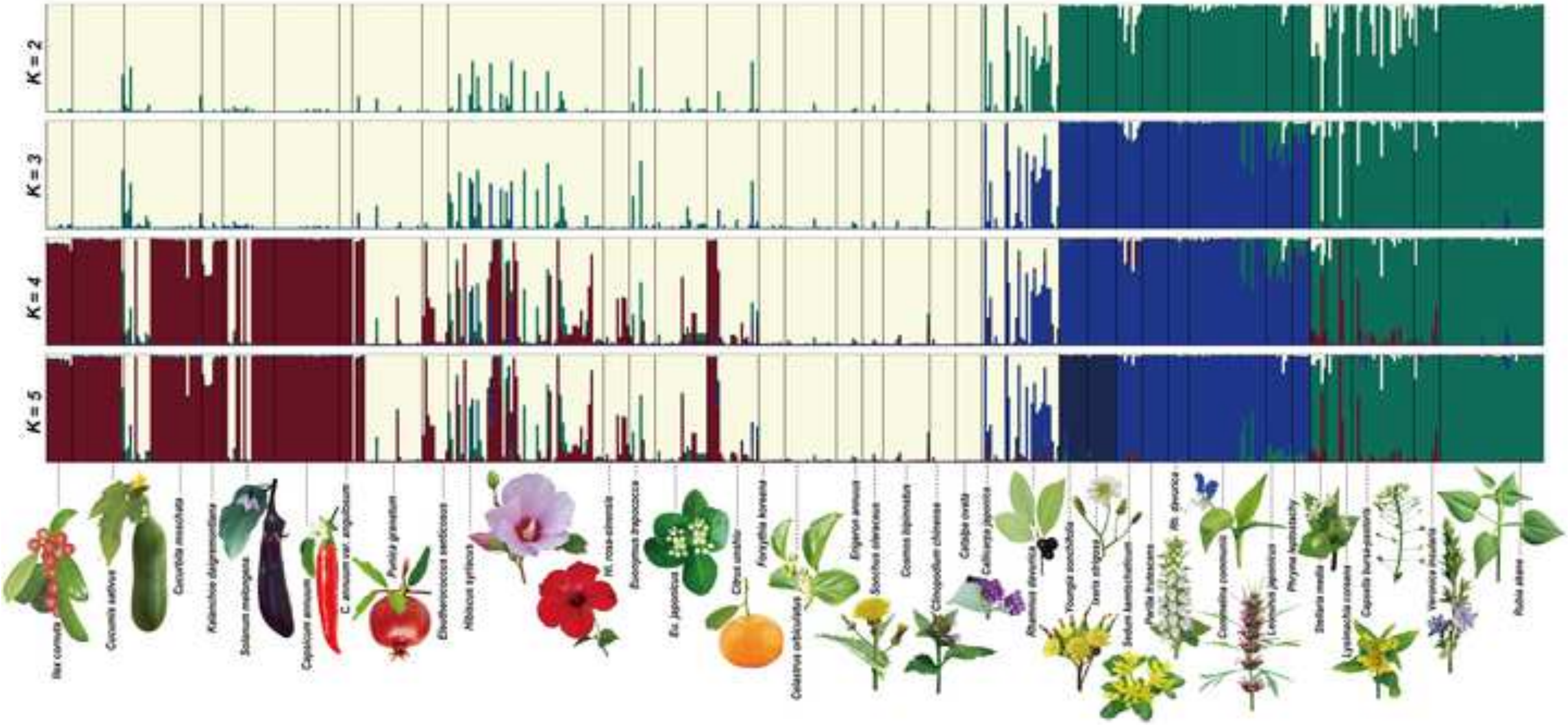

Figure 5

Click here to access/download;Figure;Fig5.tif

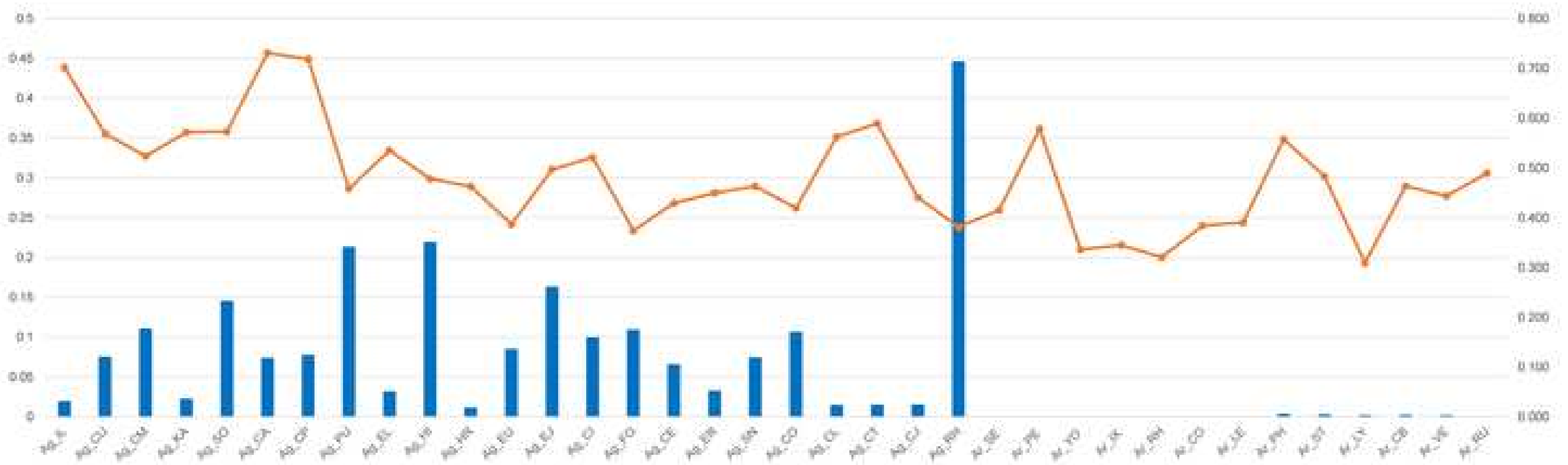

Figure 6

Click here to access/download;Figure;Fig6.tif

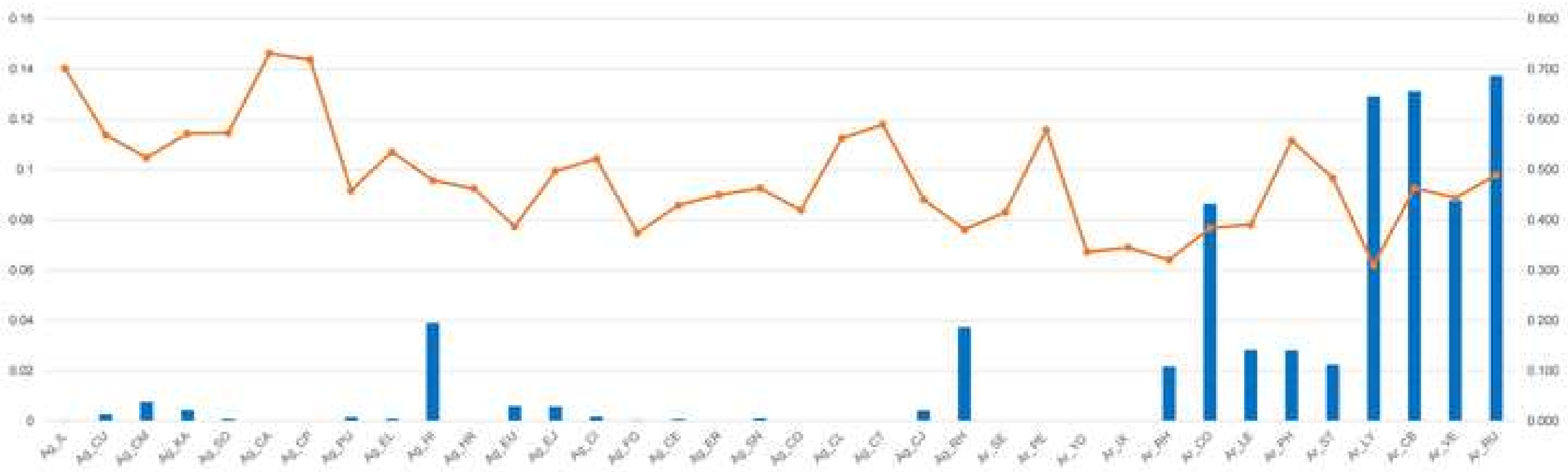

S1_Fig

Click here to access/download
**Supporting Information**
S1_Fig.tif

S2_Fig

Click here to access/download
**Supporting Information**
S2_Fig.tif

S3_Fig

Click here to access/download
**Supporting Information**
S3_Fig.tif

S4_Fig

Click here to access/download
**Supporting Information**
S4_Fig.tif

S5_Fig

Click here to access/download
**Supporting Information**
S5_Fig.tif

S6_Fig

Click here to access/download
**Supporting Information**
S6_Fig.tif

S1_Table

Click here to access/download
**Supporting Information**
S1_Table.docx

S2_Table

Click here to access/download
**Supporting Information**
S2_Table.docx

S1_File

Click here to access/download
**Supporting Information - Compressed/ZIP File Archive**
S1_File.zip

Click here to access/download
**Other**
E_200428_02_certificate_of_editing.pdf

# Complex evolution in *Aphis gossypii* group (Hemiptera: Aphididae), evidence of primary host shift and hybridization between sympatric species

**Yerim Lee[1], Thomas Thieme[2], Hyojoong Kim-[1]***

[1] Animal Systematics Lab.oratory, Department of Biology, Kunsan National University, Gunsan 54150, Republic of Korea

[2] BTL Bio-Test Labor GmbH Sagerheide, RG Phyto-Entomology, D-18190 Gross Lüsewitz, Germany

* Corresponding author

E-mail: hkim@kunsan.ac.kr **(HK)**

# Abstract

Aphids provide a good model system to understand the ecological speciation concept, since the majority of the species are host-specific, and they spend their entire lifecycle on certain groups of host plants. Aphid species that apparently have wide host plant ranges have often turned out to be complexes of host-specialized biotypes. Here we investigated the various host-associated populations of the two recently diverged species, *Aphis gossypii* and *A. rhamnicola*, having multiple primary hosts, to understand the complex evolution with host-associated speciation. Using mitochondrial DNA marker and nine microsatellite loci, we reconstructed the haplotype network, and analyzed the genetic structure and relationships. Approximate Bayesian computation was also used to infer~~used to infer~~ the ancestral primary host and host-associated divergence, which resulted in *Rhamnus* being the most ~~primitive~~ancestral host for *A. gossypii* and *A. rhamnicola*. As a result, *Aphis gossypii* and *A. rhamnicola* do not randomly use their primary and secondary host plants; rather, certain biotypes use only some secondary and specific primary hosts. Some biotypes are possibly in a diverging state through specialization to specific primary hosts. Our results also indicate that a new heteroecious ~~heteroecious~~ race can commonly be derived from the ~~heteroecy~~ heteroecious ancestor~~s~~, showing strong evidence of ecological specialization through a primary host shift in both *A. gossypii* and *A. rhamnicola*. Interestingly, *A. gossypii* and *A. rhamnicola* shared *COI* haplotypes with each other, thus there is a possibility of introgression by hybridization between them by cross-sharing same primary hosts. Our results contribute to a new perspective in the study of aphid evolution by identifying complex evolutionary trends in the *gossypii* sensu lato complex.

**KEY WORDS: host alternation, loss of primary host, molecular ecology, population genetics, speciation**

**Formatted:** Font color: Red

**Formatted:** Font color: Red

**Formatted:** Font color: Red

# Introduction

Formatted: Font: 18 pt

Phytophagous insects are a group of tremendous diversity that covers a quarter of all known terrestrial biodiversity [1, 2]. It has long been a concern to identify the evolutionary force of their remarkable diversity [3]. In most cases, phytophagous lineages have a much higher diversity than their closely related non-phytophagous lineages [3, 4]. They have an intimate relationship with certain and non-random host plant groups [5]. These findings often lead to the assumption that the host plant relationship holds the key to diversification in phytophagous insects [5, 6]. In particular, many observations of host-specific races have provided crucial evidence to support these assumptions [2, 7, 8]. Walsh [9] first proposed an ecological speciation scenario to explain the formation of sympatric host-associated populations (HAPs). The basic scenario of ecological speciation is that the transition to new host plants provides opportunities to have novel ecological niches for phytophagous insects, and contribute to different host preferences and genetic isolation; and this subsequently resulted in speciation [10].

The ecological speciation concept has also been extensively applied to explain the process of aphid speciation [11-14]. Aphids provide a good model system, since the majority of species are host-specific, and they spend their entire lifecycles on certain groups of host plants [15, 16]. Over the past decades, numerous studies have focused on the host relationship as a major factor in their speciation [15]. As one of the decisive examples, aphid species that apparently have wide host plant ranges (i.e. polyphagous) have often turned out to be complexes of host-specialized biotypes [11, 12]. It is well known that the pea aphid, *Acyrthosiphon pisum* (Harris), the most well-studied aphid species, is a set of genetically well-distinguished biotypes linked with different legume species, which can be described as an

example of sympatric speciation [11, 17-19]. A similar pattern is also found in *Uroleucon* spp., which live on certain species or some closely related plants within Asteraceae [20]. These groups of aphids show typical diversification patterns on a narrow range of related host plants (within the same family) through a trade-off in host use, gradual reduction of gene flow, and genetic drift [11, 19]. In addition, these examples are only possible if there is a mass of communities between closely related plants, which are mainly reported when many species of host plants live in similar conditions, such as asters and legumes [11, 20, 21].

In contrast to these classic examples, several species exhibit extreme polyphagous behavior, which occurs on a wide variety of unrelated plant families [15, 22]. As one of the most representative polyphagous aphids, the cotton-melon aphid, *Aphis gossypii* Glover, is associated with about 900 plants belonging to 116 plant families, including more than 100 important crops worldwide [22, 23]. The lifecycle of *A. gossypii* is as highly variable as its wide distribution range [22, 24, 25]. It has long been described as permanently anholocyclic [26], which is why studies on host change and primary hosts have rarely been conducted. First, Kring (27) reported that this aphid can perform a holocyclye in North America. Today we have to assume that *gossypii* occurs in North America, East Asia, and Europe in numerous lines, some of which have a permanent anholocyclic reproduction, while others also have a holocyclic generation cycle [28]. Those points aside, the most unusual feature of this species is that they use primary hosts belonging to various unrelated plant families (e.g. Malvaceae, Punicaceae, Rhamnaceae, Rubiaceae, and Rutaceae) [22, 26]. This is particularly interesting in evolutionary terms, and makes *A. gossypii* a good model for understanding the evolutionary process associated with the primary hosts of heteroecious aphids.

Approximately 10 % of 5,000 aphid species exhibit the seasonal host alternation (i.e. heteroecy) between primary and secondary hosts, which mysteriously are comprised with a set

Formatted: Font color: Red

of phylogenetically unrelated host plants [22, 29, 30]. In addition, ~~, of which~~ among all phytophagous insects, the complex life cycle completed by multiple generations is known to be limited to the aphids (Aphidoidea) [31, 32]. In particular, the success of species diversity and remarkable host plant relationship are believed to be attributed to multiple acquisition and the loss of heteroecy [33, 34]. Lifecycles of heteroecious aphids ~~always~~ usually comprise migration from the primary host to the secondary host [35]. For one cycle, sexual and asexual reproductions occur accompanied by several morphological changes as the generation passes [35]. Most heteroecious aphids use a much narrower range of primary host (within the same family or genus), even if they have a wide range of secondary hosts [36]. These patterns are even observed to extend to the closely related aphid species. For example, eight *Hyperomyzus* species are known to have host alternation with the primary hosts within the genus *Ribes*, even though these species have wider relationships with various secondary hosts, such as Asteraceae and Scrophulariaceae [22].

There are different views about these contrasting host ranges of primary and secondary hosts. The first view suggested that primary host specialization is in evolutionary terms more favored, because a primary host not only provides nutrients, but also a mating place [37]. For heteroecious aphids, a primary host is a place where sexual reproduction takes place, as well as the overwintering eggs hatch, and the following fundatrix stage [35, 38]. However, on the secondary host, only asexual reproduction occurs~~,~~ before the migration for overwintering [35, 38]. Thus, having different primary hosts, rather than those with different secondary hosts, may have a greater significance in reproduction. In other words, a constraint of primary host is possibly linked to the mating success of a species [15, 37]. The second view hypothesized that host alternation is only a by-product of an evolutionary process that occurs due to the phylogenetic constraints of fundatrix to the primary host [32, 33]. This is the so-called fundatrix

**Formatted:** Font color: Red

specialization hypothesis (~~Fig.~~ Fig 1), which is based on the following assumptions: i) monoecy is evolutionarily more favored than heteroecy, ii) primary hosts are more ~~primitive~~ ancestral than secondary hosts, iii) the fundatrix is highly adaptive to the primary host, but maladaptive to the secondary host, and iv) secondary hosts are more labile, and more recently obtained than primary hosts [31, 32, 39]. Under this hypothesis, the loss of a primary host was described as escape, and specialization to a specific secondary host was believed to be the only evolutionary way [31-33, 39, 40].

**Fig 1. A hypothetical scenario of the fundatrix specialization.** Concept of speciation by loss of primary host from Moran [33]~~(1988)~~.

Having multiple primary hosts in *A. gossypii* [22, 24] suggests the possibility of host-associated speciation. Indeed, previous population genetic studies have shown that *A. gossypii* is a complex of several genetically distinct biotypes associated with some plant families (e.g. Cucurbitaceae, Malvaceae, and Solanaceae) [12, 41]. However, these studies only targeted anholocyclic lineages of *A. gossypii* collected from certain crops, and only a few primary hosts (e.g. *Hibiscus syriacus* and *Punica granatum*) [12, 41]. They live on a much broader range of wild plants, and the primary hosts are also very diverse. Nevertheless, we know surprisingly little about the primary host-associated genetic structure in this species. In particular, there is no study on the genetic structure between primary and secondary HAPs of *A. gossypii*. Therefore, to better understand the evolutionary trends in *A. gossypii*, further genetic analyses encompassing wild HAPs are needed.

In addition, confirming the ~~primitive~~ ancestral host plant is crucial to understanding the evolutionary process of aphids. Among several host plants used as the primary host of *A. gossypii*, the genus *Rhamnus* (incl. *Frangula*) in Rhamnaceae is the most strongly presumed to

**Formatted:** Font color: Red

**Formatted:** Font color: Red

be a ~~primitive~~ancestral host of the *gossypii* sensu lato complex group [42, 43]. There are several reasons why *Rhamnus* is regarded as a ~~primitive~~ancestral host. The first reason is that most species belonging to the *gossypii* group show congruent use of primary hosts in *Rhamnus* [44], while the second reason is the possibility that the *gossypii* complex group and *Rhamnus* chronologically co-evolved based on molecular dating and fossil record [44, 45]. Because of this, *Rhamnus* has been believed to be at the center of the host-associated evolution in the *gossypii* complex group. Nevertheless, the relationships between *Rhamnus* and other secondary HAPs have not yet been investigated.

This study aims to investigate the evolutionary trends of the two closely-related host-alternating species, *A. gossypii* and *A. rhamnicola*, based on population genetic analyses of various primary and secondary HAPs. *Aphis gossypii* shows a typical heteroecious holocyclic lifecycle in Korea, for which various perennials and woody plants are known to be used as primary hosts [22], even though several anholocyclic isolates have been found in the secondary hosts [12]. *Aphis rhamnicola* is a recently described cryptic species of *A. gossypii* that shares *Rhamnus* spp. as ~~a p~~primary hosts, but has a somewhat different range of secondary hosts [46]. We conducted population genetic analyses of the two species in a comprehensive set of populations from primary and secondary host plants that were mostly collected from South Korea (except for one population from the UK). We used two molecular approaches in this study. First, reconstructing the haplotype network based on *COI* barcode, we confirmed their speciation pattern and genetic relationships of the aphid HAPs specialized on various host plants. Second, using nine microsatellite loci, we analyzed the genetic structure to identify the relationships between the HAPs of the two species, and to clarify the host shifting or switching process between the primary and secondary hosts. We also inferred the most likely ancestral host for the primary HAPs, which were strongly suggested through the results of this study, by

**Formatted:** Font color: Red

**Formatted:** Font color: Red

using approximate Bayesian computation methods.

# Materials and Methods

## Taxon Sampling and DNA extraction

As all collections have not been carried out in restricted areas, national parks, etc. where permits are required, it is clearly stated that there is no content regarding collection permits.

To examine the genetic structure, diversity and host-associated evolution between primary and secondary HAPs, we used 578 individual aphid samples of 36 HAPs, selectively pooled from 116 different collections, within the two species, *A. gossypii* and *A. rhamnicola*, which were collected from 36 different host plants - perennial, annual, and biannual; woody and herbaceous - in 16 plant families (Table 1). For forthcoming analyses, primary hosts and secondary hosts were defined based on the following criteria: i) The obvious primary hosts are plants that have collected sexuparae and fundatrix morphs. In addition to the obvious primary host, we also considered plants meeting the following two conditions as primary hosts. ii) Plants previously recorded as primary hosts of *A. gossypii* with reference to Inaizumi [23, 25] ~~(1981)~~ and Blackman and Eastop [28] ~~(2006)~~ or iii) the case when the collecting time is early spring (April-May) or late autumn (October-November) based on the lifecycle of *A. gossypii* on the Korean Peninsula; However, even if these two conditions were met, annual or biennial plants were excluded from the primary host. It was also not considered as the primary host if aphid collected in a greenhouse. As a consequence, all remaining plants not falling under the above conditions were considered as the secondary hosts. In our study, the host-associated population (HAP) means a collective population pooled from several temporally and/or

**Formatted:** Font: 18 pt

**Formatted:** Font: 16 pt, Bold

**Formatted:** Font: 16 pt, Not Italic

**Formatted:** Font color: Red

**Formatted:** Font color: Red

**Formatted:** Font color: Red

**Commented [오전1]:** 이 기준을 맞추다 보니 clinopodium, Sedum, Phryma, Stellaria, Capsella도 1차기주 범주에 넣게되었어요~

**Formatted:** Font color: Red

**Formatted:** Font color: Red

geographically different collections in the same plant species (Table S1). In *A. gossypii* with a large spectrum of host utilization, 25 HAPs were collected from its various primary and secondary hosts (Table S1). As *A. rhamnicola* was recently recorded found in *Rhamnus* spp., sharing and co-existing with *A. gossypii* in *Rhanmnus* as a primary host [46], nine HAPs of *A. rhamnicola* were also collected from its various primary and secondary hosts (Table S1).

These collections were acquired from South Korea, except for those of *A. gossypii* from *Catalpa ovata* in the UK (Table S1). To avoid the chance of sampling individuals from the same parthenogenetic colony, each aphid was collected from a different host plant, or a different isolated colony. All of the fresh aphid specimens used for molecular analyses were collected and preserved in (95 or 99) % ethanol, and stored at -70 °C. Total genomic DNA was extracted from single individuals using a DNeasy® Blood & Tissue Kit (QIAGEN, Inc., Dusseldorf). To preserve voucher specimens from the DNA extracted samples, we used a non-destructive DNA extraction protocol [43]. The entire body of the aphid was left in the lysis buffer with protease K solution at 55 °C for 24 h, and the cleared cuticle dehydrated.

**Formatted:** Indent: First line: 0.56", Space After: 0 pt, Line spacing: Double, No widow/orphan control, Don't adjust space between Latin and Asian text, Don't adjust space between Asian text and numbers

**Table 1. Summary statistics for microsatellite data from all aphid populations.** Number of multilocus genotypes (MLGs); genotypic diversity (GD); observed heterozygosity (Ho); expected heterozygosity (He); Hardy-Weinberg Equilibrium (HWE); gene diversity (HS); mean number of alleles (NA); allelic richness (RS). ns: non-significance in HWE (P > 0.05). **P* values for heterozygote deficit or heterozygote excess. (*P* < 0.001) [a]possibly other cryptic species A. [b]possibly other cryptic species B. [c]known as primary host ~~(Blackman and Eastop, 2006~~[28]~~)~~[25]. [d]Host type, P: perennial, A: annual, B: biennial or annual, W: woody, H: herbaceous. [e] HWE estimated ~~only including MLGs~~excluding the clonal copies of MLGs [f]*FIS* multiple loci.

| Pop. ID | No. | ~~Species~~Sorted lineage | Host plant | Host type[d] | MLGs | $N_A$ | $H_S$ | $R_S$ | *Ho* (±s.e.) | *He* (±s.e.) | HWE[e] | *FIS*[edf] |
|---|---|---|---|---|---|---|---|---|---|---|---|---|
| Ag_IL | 10 | *Aphis gossypii* Group 2 | *Ilex cornuta* | P, W | 5 | 2.33 | 0.36 | 2.12 | 0.49 (0.15) | 0.37 (0.10) | ns | -0.35 |
| Ag_CU | 20 | *Aphis gossypii* Group 2 | *Cucumis sativus* | A, H | 16 | 3.11 | 0.43 | 2.43 | 0.49 (0.11) | 0.43 (0.08) | ns | -0.16 |
| Ag_CM | 30 | *Aphis gossypii* Group 2 | *Cucurbita moschata* | A, H | 22 | 4.56 | 0.46 | 2.80 | 0.46 (0.11) | 0.46 (0.08) | ns | 0.00 |
| Ag_KA | 8 | *Aphis gossypii* Group 2 | *Kalanchoe daigremontiana* | P, W | 6 | 2.56 | 0.50 | 2.48 | 0.68 (0.14) | 0.51 (0.08) | ns | -0.35 |
| Ag_SO | 20 | *Aphis gossypii* Group 2 | *Solanum melongena* | P, W | 10 | 4.56 | 0.50 | 3.04 | 0.53 (0.13) | 0.50 (0.11) | ns | -0.07 |
| Ag_CA | 25 | *Aphis gossypii* Group 2 | *Capsicum annuum* | P, W | 6 | 2.44 | 0.39 | 2.12 | 0.64 (0.16) | 0.40 (0.10) | *excess | -0.63 |
| Ag_CP | 5 | *Aphis gossypii* Group 2 | *Capsicum annuum* var. *angulosum* | P, W | 4 | 1.89 | 0.36 | 1.89 | 0.64 (0.16) | 0.39 (0.10) | *excess | -0.79 |
| Ag_PU | 27 | *Aphis gossypii* Group 1 | *Punica granatum*[c] | P, W | 25 | 5.56 | 0.56 | 3.41 | 0.57 (0.08) | 0.56 (0.08) | ns | -0.01 |
| Ag_EL | 10 | *Aphis gossypii* Group 1 | *Eleutherococcus senticosus* | P, W | 9 | 2.67 | 0.43 | 2.40 | 0.42 (0.11) | 0.43 (0.09) | ns | 0.03 |
| Ag_HI | 60 | *Aphis gossypii* Group 1 | *Hibiscus syriacus*[c] | P, W | 59 | 7.33 | 0.50 | 3.25 | 0.49 (0.09) | 0.50 (0.08) | ns | 0.03 |
| Ag_HR | 10 | *Aphis gossypii* Group 1 | *Hibiscus rosa-sinensis*[c] | P, W | 8 | 2.22 | 0.31 | 2.02 | 0.38 (0.12) | 0.31 (0.09) | ns | -0.23 |
| Ag_EU | 10 | *Aphis gossypii* Group 1 | *Euonymus trapococca* | P, W | 9 | 3.56 | 0.52 | 3.04 | 0.56 (0.11) | 0.52 (0.10) | ns | -0.07 |
| Ag_EJ | 20 | *Aphis gossypii* Group 1 | *Euonymus japonicus* | P, W | 17 | 4.56 | 0.52 | 3.20 | 0.58 (0.10) | 0.52 (0.09) | ns | -0.12 |
| Ag_CI | 20 | *Aphis gossypii* Group 1 | *Citrus unshiu* | P, W | 16 | 4.89 | 0.51 | 3.17 | 0.57 (0.10) | 0.52 (0.06) | ns | -0.11 |
| Ag_FO | 10 | *Aphis gossypii* Group 1 | *Forsythia koreana* | P, W | 10 | 3.44 | 0.47 | 2.86 | 0.50 (0.11) | 0.47 (0.10) | ns | -0.07 |
| Ag_CE | 20 | *Aphis gossypii* Group 1 | *Celastrus orbiculatus*[c] | P, W | 19 | 4.44 | 0.46 | 2.90 | 0.52 (0.11) | 0.47 (0.09) | ns | -0.13 |
| Ag_ER | 10 | *Aphis gossypii* Group 1 | *Erigeron annuus* | A, H | 8 | 2.78 | 0.37 | 2.31 | 0.46 (0.12) | 0.37 (0.09) | ns | -0.23 |
| Ag_SN | 8 | *Aphis gossypii* Group 1 | *Sonchus oleraceus* | B, H | 7 | 3.00 | 0.45 | 2.59 | 0.50 (0.11) | 0.45 (0.08) | ns | -0.12 |
| Ag_CO | 18 | *Aphis gossypii* Group 1 | *Cosmos bipinnatus* | A, H | 16 | 3.56 | 0.51 | 2.82 | 0.48 (0.10) | 0.50 (0.08) | ns | 0.05 |
| Ag_CL | 10 | *Aphis gossypii* Group 1 | *Clinopodium chinense* var. *parviflorum* | P, H | 4 | 1.67 | 0.20 | 1.55 | 0.28 (0.11) | 0.21 (0.08) | ns | -0.37 |
| Ag_CT | 10 | *Aphis gossypii* Group 1 | *Catalpa ovata*[c] | P, W | 8 | 1.78 | 0.30 | 1.70 | 0.38 (0.11) | 0.30 (0.08) | ns | -0.26 |
| Ag_CJ | 10 | *Aphis gossypii* Group 1 | *Callicarpa japonica* | P, W | 8 | 3.11 | 0.48 | 2.64 | 0.30 (0.10) | 0.47 (0.08) | *deficit | 0.38 |
| Ag_RH | 20 | *Aphis gossypii* Group 1 | *Rhamnus davurica*[c] | P, W | 20 | 8.56 | 0.70 | 4.62 | 0.79 (0.07) | 0.70 (0.07) | ns | -0.12 |
| Ar_SE | 10 | *Aphis rhamnicola* Group 1[a] | *Sedum kamtschaticum* | P, H | 8 | 2.67 | 0.43 | 2.38 | 0.43 (0.10) | 0.43 (0.07) | ns | -0.01 |
| Ar_PE | 10 | *Aphis rhamnicola* Group 1[a] | *Perilla frutescens* var. *frutescens* | A, H | 6 | 1.89 | 0.27 | 1.77 | 0.42 (0.15) | 0.28 (0.09) | *excess | -0.54 |
| Ar_YO | 11 | *Aphis rhamnicola* Group 3[b] | *Youngia sonchifolia* | B, H | 10 | 3.67 | 0.47 | 2.90 | 0.23 (0.07) | 0.46 (0.10) | *deficit | 0.51 |
| Ar_IX | 11 | *Aphis rhamnicola* Group 3[b] | *Ixeris strigosa* | P, H | 9 | 2.89 | 0.39 | 2.39 | 0.27 (0.08) | 0.38 (0.09) | ns | 0.30 |
| Ar_RH | 8 | *Aphis rhamnicola* Group 1 | *Rhamnus davurica*[c] | P, W | 8 | 3.44 | 0.55 | 3.02 | 0.56 (0.07) | 0.55 (0.04) | ns | -0.01 |
| Ar_CO | 30 | *Aphis rhamnicola* Group 1 | *Commelina communis* | A, H | 30 | 6.67 | 0.59 | 3.58 | 0.55 (0.09) | 0.59 (0.08) | ns | 0.07 |
| Ar_LE | 10 | *Aphis rhamnicola* Group 1 | *Leonurus japonicus* | B, H | 10 | 4.11 | 0.53 | 3.07 | 0.61 (0.10) | 0.53 (0.07) | ns | -0.16 |
| Ar_PH | 7 | *Aphis rhamnicola* Group 1 | *Phryma leptostachy* | P, H | 5 | 2.33 | 0.43 | 2.23 | 0.19 (0.08) | 0.41 (0.06) | *deficit | 0.55 |
| Ar_ST | 6 | *Aphis rhamnicola* Group 2 | *Stellaria media* | B, H | 5 | 1.89 | 0.29 | 1.83 | 0.39 (0.14) | 0.30 (0.09) | ns | -0.36 |
| Ar_LY | 10 | *Aphis rhamnicola* Group 2 | *Lysimachia coreana* | P, H | 10 | 3.56 | 0.49 | 2.92 | 0.49 (0.12) | 0.49 (0.10) | ns | 0.01 |
| Ar_CB | 24 | *Aphis rhamnicola* Group 2 | *Capsella bursa-pastoris* | B, H | 24 | 4.11 | 0.50 | 2.83 | 0.55 (0.13) | 0.50 (0.10) | ns | -0.10 |
| Ar_VE | 10 | *Aphis rhamnicola* Group 2 | *Veronica insularis* | P, H | 10 | 3.22 | 0.49 | 2.79 | 0.53 (0.14) | 0.49 (0.10) | ns | -0.10 |

| | | | | | | | | | | | |
|---|---|---|---|---|---|---|---|---|---|---|---|
| Ar_RU | 40 | *Aphis rhamnicola* Group 2 | *Rubia akane* | P, W | 32 | 5.56 | 0.49 | 2.87 | 0.36 (0.08) | 0.49 (0.07) | *deficit | 0.27 |

**Formatted:** Font color: Red

**Formatted:** Font color: Red

**Formatted:** Font color: Red

**Formatted:** Centered

## Species ~~identification~~lineage sorting

In some aphid groups, morphological identification can be ambiguous, due to the lack of conclusive morphological evidence. The *Aphis* group is one of the most typical groups with the above problem. As a complementary way to avoid misidentification, host plant relationships, morphologies, and molecular tools are widely used to identify aphids [43, 46-48]. Because our study aims to demonstrate intra-specific genetic relationships based on host plant associations, species ~~accurate species identification~~lineage sorting is significant to prevent biases of the results. The two *Aphis* species, *A. gossypii* and *A. rhamnicola*, we study here are not only very similar in morphology, but also share several host plants ~~among these the two~~due to the polyphagy~~ous species~~, ~~(*A. gossypii* and *A. rhamnicola*)~~. ~~Therefore,~~Although we performed species identification through morphology and host plant relationships as a first step~~,~~ and ~~then~~ also tested DNA barcoding for all individuals collected on their shared host plants (e.g. *Capsella*, *Rhamnus*, and *Rubia*)~~.~~, we found that ~~We also performed DNA barcoding with random selection for the individuals collected from the unshared host plants.~~ However, there ~~are~~were a lot of the haplotypes cross-shared between *A. gossypii* and *A. rhamnicola* (s~~S~~ee Results). ~~In this case, exceptionally~~Therefore, instead of identifying the species ~~from~~with 36 HAPs ~~identiciation,~~ we applied the dominant assignment (white ~~or~~, green, blue, red, dark blue) of the genetic structure (*K* =3, 4, ~~2~~5) by STRUCTURE ~~and~~as well as the PCoA results (see Results) ~~for the species identification~~to sort their lineags into five groups as *Aphis gossypii* Group 1, *A. g.* Group 2~~, (see Results).~~ *A. rhmanicola* Group 1, *A. r.* Group 2 and *A. r.* Group 3 (Table 1). Accordingly, '*Aphis gossypii*' and '*A. rhamincola*', which are mentioned later, are meant to ~~have~~include all group lineages containing the HAPs assigned by the results. Table S1~~3~~ shows detailed information for lineage sorted samples used in DNA ~~barcoding~~analyses.

**Formatted:** Font: 16 pt, Not Italic
**Formatted:** Font: 16 pt, Not Italic
**Formatted:** Font color: Red
**Formatted:** Font color: Red
**Formatted:** Font color: Red
**Formatted:** Font color: Red
**Commented [오전2]:** remove
**Formatted:** Default Paragraph Font, Font: (Default) Times New Roman, 12 pt, Font color: Red
**Formatted:** Font color: Red
**Formatted:** Font color: Red
**Formatted:** Font color: Red
**Formatted:** Font color: Red
**Formatted:** Font color: Red
**Formatted:** Font color: Red
**Formatted:** Font color: Red
**Formatted:** Font color: Red
**Formatted:** Font color: Red
**Formatted:** Font color: Red
**Formatted:** Font color: Red
**Formatted:** Font: Italic
**Formatted:** Font: Italic
**Formatted:** Font color: Red
**Formatted:** Font: Italic
**Formatted:** Font: Italic
**Formatted:** Font: Italic
**Formatted:** Font: Italic
**Formatted:** Font color: Red
**Formatted:** Font color: Red
**Formatted:** Font color: Red

## Haplotype analysis

**Formatted:** Font color: Red

A 658 bp of the partial 5' region of the *cytochrome c oxidase subunit I* gene (*COI*), namely *COI* DNA barcode [49], was amplified using the universal primer sets: LEP-F1 5'-ATTCAACCAATCATAAAGATAT-3' and LEP-R1, 5'-TAAACTTCTGGATGTCCAAAAA-3'. A polymerase chain reaction (PCR) was performed with AccuPower® PCR Premix (Bioneer, Daejeon, Rep. of Korea) in 20 mL reaction mixtures under the following conditions: initial denaturation at 95 °C for 5 min; followed by 35 cycles at 94 °C for 30 s, an annealing temperature of 45.2 °C for 40 s, an extension at 72 °C for 45 s, and the final extension at 72 °C for 5 min. All PCR products were assessed using a 1.5 % agarose gel electrophoresis. Successfully amplified samples were purified using a QIAquick PCR purification kit (Qiagen, Inc.), and then immediately sequenced using an automated sequencer (ABI Prism 3730XL DNA Analyzer) at Bionics Inc. (Seoul, Korea). Both morphological identification, based on voucher specimens in the insect museum in Kunsan National University with descriptions of Blackman and Eastop (22), Lee and Kim (24), Heie (26), and molecular identification method using the *COI* DNA barcode region for comparison with the previous *COI* DNA barcode database, were used [43, 46, 50].

**Formatted:** Indent: First line: 0.56"

~~Haplotype analysis~~

**Formatted:** Font: 16 pt, Not Italic

All sequences that were obtained for DNA barcoding were initially examined and assembled using CHROMAS 2.4.4 (Technelysium Pty Ltd., Tewantin, Qld, AU) and SEQMAN PRO ver. 7.1.0 (DNA Star, Inc., Madison, Wisconsin, USA). In this step, poor-

quality sequences were discarded to avoid biases. The final dataset containing 187 sequences ~~(Table S3)~~ was aligned using MAFFT ver. 7 [51], an online utility. Some ambiguous front and back sequences were removed at this stage, resulting in sequences of 583 bp that were finally used for haplotype analysis. All sequences were deposited in GenBank (accession no. MT461429-MT461602). The *COI* haplotypes of *A. gossypii* complex were analyzed using DNASP ver. 6.12.03 [52]. A median-joining network (MJ) was built using NETWORK ver. 5.0.1.1 [53]. The MJ result was annotated with host plants or species, and then visually summarized in Fig~~s.~~ 2~~A and B~~.

## Microsatellite genotyping

**Formatted:** Font: 16 pt, Not Italic

In this study, all 578 individuals of four species were successfully genotyped using nine microsatellite loci (AGL1-2, AGL1-10, AGL1-11, AGL1-15, AGL1-16, AGL1-20, AGL1-21, AGL1-22, and AGL2-3b) previously isolated from the soybean aphid [54]. In the preliminary study, we had already checked the cross-species amplification test of these loci on *A. fabae*, *Hyalopterus pruni*, *Rhopalosiphum padi*, and *Schizaphis graminum*, as well as *A. gossypii* in the tribe Aphidini (data not shown). There were the previously developed loci from *A. gossypii* [55], but we used the nine loci developed from *A. glycines*, because we noticed that the polymorphism of the latter was higher than that of the former, which was advantageous to amplify the loci between different species. In the aphid group, several studies showed that microsatellite loci were available between related species within the aphid family as a utility of cross-species amplification [56, 57].

Microsatellite amplifications were performed using GeneAll® *Taq* DNA Polymerase Premix (GeneAll, Seoul, Korea) in 20 μL reaction mixtures containing 0.5 μM forward labeled

with a fluorescent dye (6-FAM, HEX, or NED) & reverse primers, and 0.05 µg of DNA template. PCR was performed using a GS482 thermo-cycler (Gene Technologies, Essex), according to the following procedure: initial denaturation at 95 °C for 5 min, followed by 34 cycles of 95 °C for 30 s; annealing at 56 °C for 40 s; extension at 72 °C for 45 s, and a final extension at 72 °C for 5 min. PCR products were visualized by electrophoresis on a 1.5 % agarose gel with a low-range DNA ladder to check for positive amplifications. Automated fluorescent fragment analyses were performed on the ABI PRISM 377 Genetic Analyzer (Applied Biosystems), and allele sizes of PCR products were calibrated using the molecular size marker, ROX labeled-size standard (GenScan™ ROX 500, Applied Biosystems). Raw data on each fluorescent DNA product was analyzed using GeneMapper® version 4.0 (Applied Biosystems).

## Microsatellite data analysis

Formatted: Font: 16 pt, Not Italic

Formatted: Space After: 8 pt, Widow/Orphan control, Adjust space between Latin and Asian text, Adjust space between Asian text and numbers

We used GENALEX 6.503 [58] to identify multilocus genotypes (MLGs) among populations. The program FSTAT 2.9.3.2 [59] was used to estimate the mean number of alleles ($N_A$), gene diversity ($H_S$), and allelic richness ($R_S$). Observed ($H_O$) and expected heterozygosity ($H_E$) values among loci were estimated using GENEPOP 4.0.7 [60] among the population data (HAPs) sets. Levels of significance for Hardy–Weinberg equilibrium (HWE) and linkage disequilibrium tests were adjusted using the sequential Bonferroni correction for all tests involving multiple comparisons [61]. Deviations from HWE were tested for heterozygote deficiency or excess. Because the clonal copies of MLGs due to the parthenogenetic life cycle of aphids could affect and distort the estimation of HWE [62], we used a reduced data set containing only one copy of each MLG when estimating HWE. Several assumptions of HWE

Formatted: Font: 12 pt, Font color: Red

Formatted: Font color: Red

Formatted: Font: 12 pt, Font color: Red

Formatted: Font color: Red

still can be violated, thereby these estimates are used only for descriptive purposes even although the clonal MLG copies were removed from data analysis [62]. MICRO-CHECKER [Van 63] was used to test for null alleles [64] and identify possible scoring errors, because of the large-allele dropout and stuttering.

We used ARLEQUIN 3.5.1.2 [65] for calculations of pairwise genetic differentiation ($F_{ST}$) values [66], in which populations were assigned by 36 HAPs of the two species. The statistical significance of each value was assessed by computing the pairwise comparison of the observed value in 100,000 permutations. Groupings based on ~~geographical isolations and host associations~~three different cases, (1) *gossypii* vs *rhamnicola*, (2) perennial vs non-perennial host groups in *A. gossypii*, (3) perennial vs non-perennial host groups in *A. rhamnicola*~~-~~, were tested independently with analysis of molecular variance [AMOVA; 67] in ARLEQUIN, with significance determined using the nonparametric permutation approach described by Excoffier et al. [68].

To examine the genetic relationships between 578 individual samples of four species, principal coordinate analysis (PCoA), also in GENALEX [58], further explored population relationships using the microsatellite loci, making no *a priori* assumptions about population groupings. Codominant genotypic genetic distance was calculated to make tri-matrix of pairwise populations, and then each population plot was created with coordinates based on the first two axes.

The program STRUCTURE 2.3.3 [69] was used to test for the existence of population structuring among all samples, by estimating the number of distinct populations ($K$) present in the set of samples, using a Bayesian clustering approach. We assessed likelihoods for models with the number of clusters ranging $K$ = (1 to 15). The length of the initial burn-in period was set to 100,000 iterations, followed by a run of 1,000,000 Markov chain Monte Carlo (MCMC) repetitions, of which the analysis was replicated 10 times, to ensure convergence on parameters

**Formatted:** Font: 12 pt, Font color: Red

**Formatted:** Font color: Red

**Formatted:** Font: 12 pt, Font color: Red

**Formatted:** Font color: Red

**Formatted:** Font color: Red

**Formatted:** Font: 12 pt, Font color: Red

**Formatted:** Font color: Red

and likelihood values. Parameter sets of ancestry, allele frequency, and advanced models remained as defaults. Following the method of Evanno, Regnaut (70), we calculated Δ*K* based on the second-order rate of change in the log probability of data with respect to the number of population clusters from the STRUCTURE analysis. To determine the correct value of *K*, both the likelihood distribution being to plateau or decrease [69] and the peak value of the Δ*K* statistic of Evanno, Regnaut (70) was estimated. The single run at each *K* yielding the highest likelihood of the data given the parameter values was used for plotting the distributions of individual membership coefficients (Q) with the program DISTRUCT [71].

We performed assignment tests using GENECLASS 2 [72], in which populations were assigned to 36 HAPs of the two species. For each individual of a population, the program calculates the probability of belonging to any other reference population, or of being a resident of the population where it was sampled. The sample with the highest probability of assignment was considered the most likely source for the assigned genotype. In this study, we checked the mean assignment rate from 391 *A. gossypii* or ~~145~~ 187 *A. rhamnicola* individuals into each population (source), to confirm the possible origin of each HAP. We used a Bayesian method of estimating population allele frequencies [73]. Monte Carlo re-sampling computation (100,000 simulated individuals) was used to infer the significance of assignments (alpha = 0.01).

## Approximate Bayesian computation analysis

**Formatted:** Font: 16 pt, Not Italic

To estimate the relative likelihood of the most likely ancestral HAP of *A. gossypii*, an approximate Bayesian computation (ABC) was performed for the microsatellite dataset as implemented in DIYABC version 1.0.4 [74]. DIYABC allows the comparison of complex

scenarios involving bottlenecks, serial or independent introductions, and genetic admixture events in introduced populations [75]. The parameters for modeling scenarios are the times of split or admixture events, the stable effective population size, the effective number of founders in introduced populations, the duration of the bottleneck during colonization, and the rate of admixture [76]. The software generates a simulated data set used to estimate the posterior distribution of parameters, in order to select the most likelihood scenario [76]. DIYABC generates a simulated data set that is then used to select those most similar to the observed data set, and the so-called selected data set ($n_\delta$), which are finally used to estimate the posterior distribution of parameters [74]. Recently, this ABC software package has been widely used, such as for inferring the demographic history of populations and species [77, 78], and testing potential bottleneck events [79].

To infer the most likely ~~primitive~~ancestral primary host of *A. gossypii*, among the whole microsatellite dataset, we tested three different ABC analyses using the original or partial dataset. We hypothesized the evolutionary scenarios following our results obtained from the *COI* haplotype network and two Bayesian analyses, STRUCTURE and GENECLASS2 (see “Results”). The previous studies have already revealed that *A. rhamnicola* was located in the more ~~primitive~~ancestral position within the phylogeny of the *A. gossypii* group [43, 44], which population was therefore set to the most ancestral position on the genealogy of the two ABC tests.

In the first analysis, based on the result of STRUCTURE ($K$ = 3), we compared eight evolutionary scenarios (A1–A8) using a dataset that included 578 individuals from four population groups, which consisted of 75 individuals from the ‘BLUE’ group (A~~g~~r_SE, A~~g~~r_PE, A~~n~~r_IX, A~~n~~r_YO, Ar_CO, Ar_PH, Ar_RH, Ar_LE), 90 from the ‘GREEN’ group (Ar_ST, Ar_VE, Ar_LY, Ar_CB, Ar_RU), 30 from the ‘MIXBW (BLUE+WHITE)’ group (Ag_RH, Ag_CJ), and 361 from the ‘WHITE’ group (Ag-IL, Ag_CE, Ag_EU, Ag_EJ, Ag_PU,

**Formatted:** Font color: Red

**Formatted:** Font color: Red

Ag_CU, Ag_CM, Ag_KA, Ag_EL, Ag_HI, Ag_HR, Ag_FO, Ag_CI, Ag_ER, Ag_SN, Ag_CO, Ag_SO, Ag_CA, Ag_CP, Ag_CL, Ag_CT) (~~Fig.~~ Fig S1). Scenario A1 considered (1) GREEN originated from BLUE, (2) MIXBW subsequently originated from GREEN, and then (3) WHITE originated from MIXBW. Scenario A2 considered (1) WHITE originated from BLUE, (2) MIXBW subsequently originated from WHITE, and then (3) GREEN originated from MIXBW. Scenario A3 considered (1) BLUE originated from GREEN, (2) MIXBW subsequently originated from BLUE, and then (3) WHITE originated from MIXBW. Scenario A4 considered (1) WHITE originated from MIXBW, (2) BLUE subsequently originated from WHITE, and then (3) GREEN originated from BLUE. Scenario A5 considered (1) GREEN originated from MIXBW, (2) BLUE subsequently originated from GREEN, and then (3) WHITE originated from BLUE. Scenario A6 considered (1) GREEN originated from WHITE, (2) MIXBW subsequently originated from GREEN, and then (3) BLUE originated from MIXBW. Scenario A7 considered (1) BLUE originated from MIXBW, (2) GREEN subsequently originated from BLUE, and then (3) WHITE originated from GREEN. Scenario A8 considered (1) BLUE originated from WHITE, (2) MIXBW subsequently originated from BLUE, and then (3) GREEN originated from MIXBW.

In the second analysis, based on the result of STRUCTURE ($K$ = 4), we compared six evolutionary scenarios (B1–B6) using a dataset that included 311 individuals from four population groups, which consisted of 75 individuals from the ‘BLUE’ group (Ar_CO, Ar_PH, Ar_RH, A~~g~~r_SE, A~~g~~r_PE, Ar_LE), 90 from the ‘GREEN’ group (Ar_ST, Ar_VE, Ar_LY, Ar_CB, Ar_RU), 60 from the ‘RED’ group (Ag_IL, Ag_CU, Ag_CA, Ag_CP), and 86 from the ‘WHITE’ group (Ag_CE, Ag_FO, Ag_ER, Ag_SN, Ag_CO, Ag_CL, Ag_CT) (~~Fig.~~ Fig S2). Scenario B1 considered (1) GREEN originated from BLUE, (2) WHITE subsequently originated from GREEN, and then (3) RED originated from WHITE. Scenario B2 was basically similar to scenario B1, except for (1) WHITE originated from RED. Scenario B3 considered

(1) WHITE originated from RED, (2) GREEN subsequently originated from WHITE, and then (3) BLUE originated from GREEN. Scenario B4 considered (1) GREEN originated from BLUE, (2) WHITE formerly originated from GREEN, and then (3) RED later originated from GREEN. Scenario B5 considered (1) GREEN originated from BLUE, (2) RED formerly originated from GREEN, and then (3) WHITE later originated from GREEN. Scenario B6 considered (1) WHITE originated from RED, (2) GREEN formerly originated from WHITE, and then (3) BLUE later originated from GREEN.

In the third analysis, based on the result of STRUCTURE (*K* = 4), we compared six evolutionary scenarios (C1–C6) using a dataset including 391 individuals from four population groups, except for BLUE and GREEN groups in the first and second analysis, which consisted of 30 individuals from the 'MBW (BLUE+WHITE)' group (Ag_RH, Ag_CJ), 207 from the 'MRW (RED+WHITE)' group (Ag_EU, Ag_EJ, Ag_PU, Ag_SO, Ag_CM, Ag_EL, Ag_HI, Ag_HR, Ag_CI), 68 from the 'RED' group (Ag~~-~~_IL, Ag_CU, Ag_KA, Ag_CA, Ag_CP), and 86 from the 'WHITE' group (Ag_CE, Ag_FO, Ag_ER, Ag_SN, Ag_CO, Ag_CL, Ag_CT) (~~Fig.~~ Fig S3). Scenario C1 considered (1) MRW originated from MBW, (2) WHITE subsequently originated from MRW, and then (3) RED originated from WHITE. Scenario C2 was basically similar to scenario C1, except for (3) WHITE originated from RED. Scenario C3 considered (1) WHITE originated from MBW, (2) RED subsequently originated from WHITE, and then (3) MRW originated from RED. Scenario C4 was basically similar to scenario C3, except for (2) MRW subsequently originated from WHITE, and then (3) RED originated from MRW. Scenario C5 considered (1) RED originated from MBW, (2) WHITE subsequently originated from RED, and then (3) MRW originated from WHITE. Scenario C6 was basically similar to scenario C5, except for (3) WHITE originated from MRW.

We produced 1 000 000 simulated data sets for each scenario. We used a generalized stepwise model (GSM) as the mutational model for microsatellites, which assumes increases

or reductions by single repeat units [74]. To identify the posterior probability (PP) of these three scenarios, the $n_{\delta}$ = 30 000 (1 %) simulated datasets closest to the pseudo-observed dataset were selected for the logistic regression, which is similar to the $n_{\delta}$ = 300 (0.01 %) ones for the direct approach [76]. The summary of statistics was calculated from the simulated and observed data for each of the tested scenarios, such as the mean number of alleles per locus (*A*), mean genetic diversity for each group and between group, genetic differentiation between pairwise groups ($F_{ST}$), classification index, shared alleles distance ($D_{AS}$), and Goldstein distance.

# Results

**Formatted:** Font: 18 pt

## Haplotype analysis

**Formatted:** Font: 16 pt

A total of 18 haplotypes were recognized from the 187 *COI* sequences of 36 host-associated populations of the two *Aphis* species (Fig~~s.~~ 2~~A and B~~). The most common haplotype was H9, followed by H2. Aphid samples from the three primary hosts: *Hibiscus*, *Rhamnus*, and *Rubia* were spread across these two major haplotypes (~~Fig.~~ Fig 2A). All the samples from the remaining primary hosts (i.e. *Catalpa*, *Celastrua*, *Citrus*, *Euonymus*, and *Punica*) had H9 haplotype (~~Fig.~~ Fig 2A). Unique haplotypes were mostly observed among the *Rubia* population (~~Fig.~~ Fig 2A). *Cucurbita*, *Hibiscus*, *Punica*, *Rhamnus*, *Sedum*, and *Youngia* associated populations also had unique haplotypes. Among the secondary HAPs, samples from the *Capsella* were found in both H2 and H9 haplotypes (~~Fig.~~ Fig 2A). Samples from the *Youngia* was also observed in H1, H2, and H3 haplotypes (~~Fig.~~ Fig 2A). However, the populations

associated with the majority of secondary hosts only had one haplotype. H1 consisted of samples from secondary hosts, such as *Ixeris*, *Leonurus*, *Perilla*, *Phryma*, and *Youngia*. To compare *COI* haplotype and microsatellite genotype results, we overlaid the five biotypes that were identified from STRUCTURE ($K$ = 5) on the haplotype network (~~Fig.~~ Fig 2B). The result of haplotype analysis was highly discordant with the STRUCTURE results (see below). Among the five biotypes, red, blue, and green types were observed in both H2 and H9 haplotypes (~~Fig.~~ Fig 2B). The majority of white type aphids belonged to H9, while blue and green types were mostly found in H2 (~~Fig.~~ Fig 2B). Aphids with green type showed the most diverse haplotype diversity (~~Fig.~~ Fig 2B). The haplotype H1 contained blue and dark blue types.

**Fig 2. (A) Haplotype network for *COI* DNA barcode dataset (583 bp) using NETWORK ver. 5.0.1.1 [80]~~(Bandelt et al., 1999)~~.** Pie chart distribution based on each HAP~~ in the two species~~; **(B) Haplotype network for *COI* DNA barcode dataset (583 bp) using NETWORK.** Pie chart distribution based on each ~~species or~~ group ~~within the species~~of five lineages in the two species.

## Microsatellite data analysis

We successfully genotyped 578 aphid individuals of 36 HAPs of the two species using 9 microsatellite loci, and then found 463 non-clonal mLGs from all samples (Table 1). Generally, genetic diversity was high throughout the HAPs collected from woody perennials, which seemed to be regarded as the primary (overwintering) hosts. The mean number of alleles ($N_A$) and gene diversity ($H_S$) in *A. gossypii* host populations averaged (4.17 and 0.45), respectively, whereas *A. rhamnicola* populations averaged (4.98 and 0.48), respectively. Similarly, allelic richness ($R_S$) ($R_S$, mean ± s.d., 2.67 ± 0.67) in the *A. gossypii* populations was

**Formatted:** Font color: Red

**Formatted:** Font color: Red

**Formatted:** Font color: Red

**Formatted:** Font: 16 pt

**Formatted:** Space After: 0 pt, No widow/orphan control, Don't adjust space between Latin and Asian text, Don't adjust space between Asian text and numbers

slightly lower than $R_S$ (2.79 ± 0.50) in those of *A. rhamnicola*. Surprisingly, among all HAPs, Ag_RH, Ag_HI, and Ar_CO had relatively very high $N_A$ at (8.56, 7.33, and 6.67), respectively, of which the $R_S$ values were also high at (4.62, 3.25, and 3.58), respectively. The expected heterozygosity ($H_E$) values in the *A. gossypii* populations ranged (0.21 to 0.70), whereas $H_E$ values in the *A. rhamnicola* populations ranged (0.30 to 0.59). In HWE, there were significant deviations in Ag_CA, Ag_CP, and Agr_PE by heterozygote excess, and in Ag_CJ, Agr_YO, Ar_PH, and Ar_RU by heterozygote deficit. Heterozygote excess in Ag_CA, Ag_CP, and Agr_PE were likely the result of heterosis or over-dominance related to selection preference toward heterozygous combination or fixation of heterozygous genotypes due to parthenogenetic lifesis of aphids in secondary host, especially under anholocyclic (permanently asexual) life [81], or fixation of heterozygous genotypes; and, correspondingly, . Similar to our results, this phenomenon was already reported from several aphid species such as *Sitobion avenae*, *Myzus persicae* and *Rhopalosiphum padi* having permanently or temporary asexual life, which showed the significant heterozygote excess [82-84]. nNegative *FIS* values also showed an increase in heterozygosity that was generally due to random mating or outbreeding, where as positive *FIS* values explained that the amount of heterozygous offspring in the population decreased, usually due to inbreeding [85]. In contrast, heterozygote deficit (i.e., homozygote excess) in Ag_CJ, Agr_YO, Ar_PH, and Ar_RU was likely caused by retaining numerous unique genotypes with private alleles within a population, and positive *FIS* values explained that the amount of heterozygous offspring in the population decreased, usually due to inbreeding [85]. There was no evidence of significant linkage disequilibrium or frequency of null alleles.

## Genetic differentiation between host-associated populations and AMOVA

**Formatted:** Font color: Red

**Formatted:** Font color: Red

**Formatted:** Font color: Red

**Formatted:** Font: Italic

**Formatted:** Font: Italic

**Formatted:** Font color: Red

**Formatted:** Font color: Red

**Formatted:** Font color: Red

**Formatted:** Font: 14 pt, Bold, Not Italic

We estimated pairwise genetic differentiation ($F_{ST}$) between 36 different HAPs of the two species (Table 2). The averaging pairwise $F_{ST}$ values among the HAPs of all, only *A. gossypii* (Ar_SE, Ar_PE, Ar_YO, and Ar_IX) and only *A. rhamnicola* were 0.329, 0.209 and 0.392, respectively. In *A. gossypii*, it appeared that the averaging pairwise $F_{ST}$ values among the different HAPs obtained from host plants within the same plant genus or family were relatively low.The average $F_{ST}$ values among the HAPs of *A. gossypii* (Ag_SE, Ag_PE, Ag_YO, and Ag_IX) and *A. rhamnicola* were (0.209, 0.407, and 0.388), respectively. In *A. gossypii*, individuals of different HAPs obtained from host plants within the same plant genus or family were mostly merged into one HAP, such as Cucurbitaceae (Ag_CU, Ag_CM; averaging pairwise $F_{ST}$ = 0.040), Solanaceae (Ag_SO, Ag_CA, Ag_CP; 0.029), *Euonymus* (Ag_EU, Ag_EJ; 0.016), and Asteraceae (Ag_ER, Ag_SN, Ag_CO; 0.025). Remarkably, Ag_PU (0.130), Ag_HI (0.152), Ag_FO (0.159), and Ag_RH (0.134), considered to be the primary host, showed relatively low average $F_{ST}$ values toward the other *A. gossypii* populations. In *A. rhamnicola*, Ar_LY, Ar_CB, and Ar_VE were genetically close (0.050) to each other, despite belonging to the same host family/genus or being locally similar. In addition, Ar_RH was close to Ar_CO (0.089). Between *A. gossypii* and *A. rhamnicola* populations, Ar_ST and Ag_HI showed the lowest $F_{ST}$ value (0.095).

Two cases to confirm the genetic variance between the preordained groups were analyzed using AMOVA implemented in ARLEQUIN [67]. In the case of the analysis grouped by host plantscase 1, the percentages of the genetic variance (PV) 'among groups' and 'among populations within groups' were similar at about 18 %14.59 % and 22.60 %, respectively, which shows that there is some grouping effect by host plants, even though the majority of genetic variation was found 'among individuals within populations' as approximately 623 % (Table 3). However, the genetic variance of about -1 ~ 0 % 'among groups' and 6 % 'among

Formatted: Font color: Red

Commented [오전3]: 삭제

Formatted: Font color: Red

Formatted: Font color: Red

Formatted: Font color: Red

Formatted: Font color: Red

~~populations within groups'~~ in the both ~~the case of the~~ analys~~i~~es grouped by ~~geographic location~~cases 2 and 3 suggests that there are no ~~regional~~ grouped structures~~structures between the compared locations~~according to their lives in the perennial or non-perennial hosts on both *A. gossypii* and *A. rhamnicola* (Table 3). Interestingly, PV of 'among populations within groups' in *A. rhamnicola* was about 20 % higher than that in *A. gossypii*, which means that the HAPs of *A. rhamnicola* is genetically differentiated further than those of *A. gossypii* (Table 3).

**Formatted:** Font: Italic, Font color: Red
**Formatted:** Font color: Red
**Formatted:** Font: Italic, Font color: Red
**Formatted:** Font color: Red
**Formatted:** Font: Italic, Font color: Red
**Formatted:** Font color: Red
**Formatted:** Font: Italic, Font color: Red
**Formatted:** Font color: Red
**Formatted:** Font: Italic, Font color: Red
**Formatted:** Font color: Red
**Formatted:** Font: Italic, Font color: Red
**Formatted:** Font color: Red

**Table 2. Pairwise $F_{ST}$ divergence between 36 ~~host-associated populations~~HAPs of the two species, *A. gossypii* groups (Ag) and *A. rhamnicola* groups (Ar)**. Values are significantly different from zero at $P <$ 0.05 unless indicated as bold and italic

Formatted: Font color: Red

| | Ag_IL | Ag_CU | Ag_CM | Ag_KA | Ag_SO | Ag_CA | Ag_CP | Ag_PU | Ag_EL | Ag_HI | Ag_HR | Ag_EU | Ag_EJ | Ag_CI | Ag_FO | Ag_CE | Ag_ER | Ag_SN | Ag_CO |
|---|---|---|---|---|---|---|---|---|---|---|---|---|---|---|---|---|---|---|---|
| Ag_CU | 0.230 | | | | | | | | | | | | | | | | | | |
| Ag_CM | 0.183 | 0.040 | | | | | | | | | | | | | | | | | |
| Ag_KA | 0.202 | 0.145 | 0.092 | | | | | | | | | | | | | | | | |
| Ag_SO | 0.217 | 0.147 | 0.101 | 0.106 | | | | | | | | | | | | | | | |
| Ag_CA | 0.339 | 0.223 | 0.206 | 0.199 | 0.066 | | | | | | | | | | | | | | |
| Ag_CP | 0.352 | 0.209 | 0.187 | 0.163 | ***0.049*** | ***-0.030*** | | | | | | | | | | | | | |
| Ag_PU | 0.194 | 0.196 | 0.140 | 0.128 | 0.117 | 0.234 | 0.219 | | | | | | | | | | | | |
| Ag_EL | 0.387 | 0.267 | 0.203 | 0.140 | 0.224 | 0.337 | 0.311 | 0.216 | | | | | | | | | | | |
| Ag_HI | 0.169 | 0.137 | 0.060 | 0.090 | 0.121 | 0.244 | 0.216 | 0.111 | 0.196 | | | | | | | | | | |
| Ag_HR | 0.350 | 0.293 | 0.255 | 0.291 | 0.329 | 0.453 | 0.486 | 0.202 | 0.384 | 0.223 | | | | | | | | | |
| Ag_EU | 0.227 | 0.206 | 0.096 | 0.128 | 0.120 | 0.252 | 0.227 | 0.060 | 0.165 | 0.061 | 0.305 | | | | | | | | |
| Ag_EJ | 0.173 | 0.178 | 0.090 | 0.141 | 0.094 | 0.231 | 0.211 | 0.046 | 0.216 | 0.048 | 0.258 | 0.016 | | | | | | | |
| Ag_CI | 0.254 | 0.240 | 0.160 | 0.132 | 0.116 | 0.219 | 0.204 | 0.061 | 0.206 | 0.144 | 0.327 | 0.067 | 0.093 | | | | | | |
| Ag_FO | 0.234 | 0.260 | 0.159 | 0.184 | 0.149 | 0.300 | 0.295 | ***0.017*** | 0.266 | 0.106 | 0.235 | 0.064 | 0.040 | 0.087 | | | | | |
| Ag_CE | 0.299 | 0.295 | 0.194 | 0.169 | 0.215 | 0.332 | 0.312 | 0.134 | 0.114 | 0.152 | 0.283 | 0.090 | 0.124 | 0.119 | 0.137 | | | | |
| Ag_ER | 0.329 | 0.327 | 0.225 | 0.269 | 0.178 | 0.339 | 0.351 | 0.066 | 0.369 | 0.151 | 0.389 | 0.093 | 0.065 | 0.121 | 0.084 | 0.240 | | | |
| Ag_SN | 0.214 | 0.246 | 0.158 | 0.202 | 0.160 | 0.300 | 0.289 | 0.036 | 0.294 | 0.106 | 0.307 | ***0.045*** | 0.027 | 0.094 | 0.039 | 0.169 | ***0.028*** | | |
| Ag_CO | 0.204 | 0.240 | 0.161 | 0.179 | 0.146 | 0.287 | 0.274 | 0.015 | 0.279 | 0.100 | 0.237 | 0.057 | 0.033 | 0.086 | ***-0.008*** | 0.162 | 0.040 | ***0.008*** | |
| Ag_CL | 0.563 | 0.499 | 0.404 | 0.391 | 0.422 | 0.516 | 0.584 | 0.331 | 0.193 | 0.362 | 0.577 | 0.310 | 0.361 | 0.290 | 0.408 | 0.147 | 0.531 | 0.457 | 0.398 |
| Ag_CT | 0.432 | 0.353 | 0.279 | 0.311 | 0.271 | 0.346 | 0.386 | 0.191 | 0.371 | 0.257 | 0.435 | 0.214 | 0.209 | 0.176 | 0.254 | 0.241 | 0.297 | 0.232 | 0.232 |
| Ag_CJ | 0.234 | 0.255 | 0.205 | 0.225 | 0.201 | 0.308 | 0.301 | 0.119 | 0.342 | 0.170 | 0.312 | 0.176 | 0.127 | 0.213 | 0.136 | 0.264 | 0.213 | 0.126 | 0.115 |
| Ag_RH | 0.209 | 0.200 | 0.144 | 0.120 | 0.111 | 0.215 | 0.185 | 0.037 | 0.181 | 0.121 | 0.188 | 0.063 | 0.072 | 0.085 | 0.045 | 0.128 | 0.107 | 0.085 | 0.058 |
| Ar_SE | 0.473 | 0.439 | 0.400 | 0.351 | 0.344 | 0.417 | 0.403 | 0.304 | 0.411 | 0.361 | 0.515 | 0.359 | 0.335 | 0.356 | 0.367 | 0.379 | 0.435 | 0.392 | 0.365 |
| Ar_PE | 0.626 | 0.572 | 0.546 | 0.549 | 0.524 | 0.591 | 0.625 | 0.478 | 0.587 | 0.492 | 0.659 | 0.533 | 0.507 | 0.522 | 0.572 | 0.547 | 0.623 | 0.587 | 0.530 |
| Ar_YO | 0.469 | 0.411 | 0.411 | 0.397 | 0.421 | 0.479 | 0.468 | 0.374 | 0.466 | 0.387 | 0.496 | 0.412 | 0.395 | 0.428 | 0.448 | 0.457 | 0.491 | 0.435 | 0.415 |
| Ar_IX | 0.502 | 0.436 | 0.424 | 0.429 | 0.446 | 0.509 | 0.516 | 0.400 | 0.507 | 0.397 | 0.534 | 0.437 | 0.415 | 0.458 | 0.486 | 0.484 | 0.526 | 0.473 | 0.444 |
| Ar_RH | 0.463 | 0.428 | 0.383 | 0.338 | 0.348 | 0.437 | 0.393 | 0.347 | 0.396 | 0.330 | 0.490 | 0.337 | 0.352 | 0.382 | 0.387 | 0.390 | 0.449 | 0.403 | 0.383 |
| Ar_CO | 0.444 | 0.410 | 0.387 | 0.357 | 0.355 | 0.419 | 0.389 | 0.362 | 0.388 | 0.370 | 0.452 | 0.353 | 0.368 | 0.382 | 0.389 | 0.394 | 0.429 | 0.397 | 0.388 |
| Ar_LE | 0.474 | 0.437 | 0.413 | 0.368 | 0.411 | 0.491 | 0.443 | 0.376 | 0.398 | 0.348 | 0.509 | 0.363 | 0.369 | 0.410 | 0.430 | 0.394 | 0.481 | 0.427 | 0.410 |
| Ar_PH | 0.487 | 0.443 | 0.416 | 0.394 | 0.389 | 0.484 | 0.475 | 0.358 | 0.462 | 0.339 | 0.545 | 0.382 | 0.362 | 0.411 | 0.431 | 0.414 | 0.491 | 0.428 | 0.389 |
| Ar_ST | 0.356 | 0.301 | 0.191 | 0.239 | 0.313 | 0.444 | 0.458 | 0.263 | 0.367 | 0.095 | 0.440 | 0.216 | 0.208 | 0.295 | 0.294 | 0.274 | 0.387 | 0.304 | 0.270 |
| Ar_LY | 0.451 | 0.392 | 0.347 | 0.311 | 0.365 | 0.437 | 0.407 | 0.351 | 0.397 | 0.265 | 0.483 | 0.328 | 0.320 | 0.384 | 0.397 | 0.382 | 0.450 | 0.405 | 0.382 |
| Ar_CB | 0.406 | 0.361 | 0.321 | 0.282 | 0.355 | 0.425 | 0.395 | 0.344 | 0.366 | 0.239 | 0.436 | 0.318 | 0.315 | 0.363 | 0.377 | 0.355 | 0.432 | 0.389 | 0.367 |
| Ar_VE | 0.442 | 0.375 | 0.335 | 0.295 | 0.360 | 0.448 | 0.414 | 0.341 | 0.379 | 0.252 | 0.469 | 0.328 | 0.312 | 0.378 | 0.386 | 0.371 | 0.436 | 0.389 | 0.372 |
| Ar_RU | 0.451 | 0.417 | 0.383 | 0.381 | 0.389 | 0.463 | 0.442 | 0.352 | 0.421 | 0.333 | 0.456 | 0.343 | 0.336 | 0.381 | 0.380 | 0.376 | 0.417 | 0.388 | 0.362 |

**Table 2. (continued)**

| | Ag_CL | Ag_CT | Ag_CJ | Ag_RH | Ar_SE | Ar_PE | Ar_YO | Ar_IX | Ar_RH | Ar_CO | Ar_LE | Ar_PH | Ar_ST | Ar_LY | Ar_CB | Ar_VE |
|---|---|---|---|---|---|---|---|---|---|---|---|---|---|---|---|---|
| Ag_CT | 0.502 | | | | | | | | | | | | | | | |
| Ag_CJ | 0.520 | 0.340 | | | | | | | | | | | | | | |
| Ag_RH | 0.291 | 0.182 | 0.130 | | | | | | | | | | | | | |
| Ar_SE | 0.563 | 0.508 | 0.413 | 0.248 | | | | | | | | | | | | |
| Ar_PE | 0.720 | 0.667 | 0.561 | 0.399 | 0.549 | | | | | | | | | | | |
| Ar_YO | 0.617 | 0.521 | 0.407 | 0.307 | 0.494 | 0.570 | | | | | | | | | | |
| Ar_IX | 0.657 | 0.559 | 0.449 | 0.338 | 0.529 | 0.617 | 0.187 | | | | | | | | | |
| Ar_RH | 0.562 | 0.508 | 0.377 | 0.200 | 0.387 | 0.532 | 0.438 | 0.480 | | | | | | | | |
| Ar_CO | 0.492 | 0.456 | 0.381 | 0.209 | 0.383 | 0.440 | 0.418 | 0.447 | 0.089 | | | | | | | |
| Ar_LE | 0.560 | 0.531 | 0.407 | 0.294 | 0.410 | 0.428 | 0.454 | 0.470 | 0.292 | 0.317 | | | | | | |
| Ar_PH | 0.628 | 0.575 | 0.339 | 0.291 | 0.462 | 0.521 | 0.484 | 0.532 | 0.320 | 0.331 | 0.340 | | | | | |
| Ar_ST | 0.596 | 0.507 | 0.321 | 0.233 | 0.501 | 0.662 | 0.500 | 0.520 | 0.400 | 0.413 | 0.377 | 0.422 | | | | |
| Ar_LY | 0.573 | 0.495 | 0.403 | 0.267 | 0.418 | 0.570 | 0.455 | 0.463 | 0.295 | 0.327 | 0.253 | 0.384 | 0.245 | | | |
| Ar_CB | 0.509 | 0.467 | 0.391 | 0.280 | 0.423 | 0.535 | 0.446 | 0.441 | 0.314 | 0.347 | 0.253 | 0.360 | 0.178 | ***0.000*** | | |
| Ar_VE | 0.569 | 0.504 | 0.388 | 0.269 | 0.412 | 0.570 | 0.450 | 0.460 | 0.299 | 0.342 | 0.243 | 0.370 | 0.206 | ***0.023*** | 0.027 | |
| Ar_RU | 0.503 | 0.484 | 0.380 | 0.303 | 0.453 | 0.528 | 0.452 | 0.479 | 0.383 | 0.407 | 0.363 | 0.358 | 0.323 | 0.343 | 0.329 | 0.308 |

**Table 3. Analysis of molecular variance (AMOVA) results for microsatellite data analysis of aphids grouped by three cases:** (~~A~~1) *gossypii* vs *rhamnicola*, (~~B~~2) perennial vs non-perennial host groups in *A. gossypii*, (~~C~~3) perennial vs non-perennial host groups in *A. rhamnicola*. $F_{st}$: among groups, $F_{sc}$: among populations within groups, $F_{ct}$: within populations, V: variance components. PV: percentage of variation.

| | Among groups | | | Among populations within groups | | | Within populations | | |
|---|---|---|---|---|---|---|---|---|---|
| Case | $V_a$ | PV | *P* | $V_b$ | PV | *P* | $V_c$ | PV | *P* |
| 1 | 0.50 | 14.59 | <0.0001 | 0.77 | 22.60 | <0.0001 | 2.14 | 62.81 | <0.0001 |
| 2 | -0.01 | -0.55 | <0.0001 | 0.48 | 18.54 | <0.0001 | 2.13 | 82.01 | 0.4458 |
| 3 | -2.59 | -0.09 | 0.0001 | 1.47 | 41.55 | <0.0001 | 2.16 | 61.04 | 0.8602 |

Formatted: Font color: Red

Formatted: Font color: Red

Formatted: Font color: Red

Formatted: Font color: Red

Formatted: Font color: Red

Formatted: Font color: Red

## Genetic similarity, structure, and assignment

A plot of PCoA between 36 HAPs based on codominant genotypic genetic distances showed that the two species, *A. gossypii* and *A. rhamnicola*, were completely separated in each of the left, upper–right, and lower–right sides on the plot (~~Fig. ~~Fig 3). Plots of *A. gossypii* populations being closely aggregated along the line of factor 1 means that they are genetically close to each other, whereas the plots of ~~*the other remaining HAPs*~~*A. rhamnicola* being relatively largely scattered show genetic isolations between them. Among all *A. gossypii* populations, Ag_HI and Ag_RH were relatively located near to the HAPs of *A. rhamnicola*, Ar_ST~~,~~ and A~~g~~r_SE, respectively. Plots of A~~g~~r_YO and A~~g~~r_IX, which had been taxonomically considered to *A. gossypii*, were closely located to each other, but distant from the majority group of *A. gossypii*.

**Fig 3. A plot of the principal coordinate analysis based on the first two factors for 578 individuals of the four *gossypii* group species.** Each color corresponds to that shown in the results of STRUCTURE when *K* = 3 (Fig 3); white~~red – *A. gossypii* with~~ 23 HAPs of *A. gossypii*; blue – *Rhamnus* group, ~~A. gossypii (Ar_SE, Ar_PE, Ar_YO and Ar_IX) + 4 host-associated populations8~~7 HAPs of *A. rhamnicola*; green – *Rubia* group, ~~5~~6 HAPs of *A. rhamnicola*. First and second coordinate axes account for (26.13 and 11.90) %, respectively.

The genetic structure of 36 HAPs of the two species (*A. gossypii* and *A. rhamnicola*) for 578 individuals was analyzed by STRUCTURE 2.3.3 [69]. In all STRUCTURE analyses from *K* = (1 to 15), the most likely number of clusters was *K* = 4, using the Δ*K* calculation according to the method of Evanno, Regnaut (70). Here, we show the structure results from *K*

**Formatted:** Font: 14 pt, Bold, Not Italic

**Formatted:** Indent: First line: 0"

**Formatted:** Font: Italic, Font color: Red

**Formatted:** Font color: Red

**Formatted:** Font color: Red

**Formatted:** Font: Italic, Font color: Red

**Formatted:** Font color: Red

**Formatted:** Font: Not Italic

**Formatted:** Font: Not Italic

**Formatted:** Font: Not Italic

= (2 to 5), in order to observe the change of genetic structure and assignment pattern according to the *K* value (~~Fig. ~~Fig 4). When *K* = 2, the (first) white cluster dominantly appeared to *A. gossypii* populations, except for Ag_RH with a large green assignment, while the (second) green cluster was largely distributed among populations of ~~*A. gossypii* (Agr_SE, Agr_PE, Agr_YO, and Agr_IX) and~~ *A. rhamnicola*. When *K* = 3, the (first) white cluster also was dominant in *A. gossypii* HAPs, except for Ag_RH with large blue assignment and Ag_Hi with small green and blue ones, the (third) blue cluster as the '*Rhamnus* group' prevalent in ~~*A. gossypii* (~~Agr_SE, Agr_PE, Agr_YO, ~~and~~ Agr_IX~~)~~, Ar_RH, Ar_CO, Ar_LE, Ar_PH~~,~~ and Ar_ST, and the (second) green cluster in the rest as the '*Rubia* group'. When *K* = ~~3~~4, the genetic structure was basically similar to that at *K* = ~~4~~3, except that the (fourth) red cluster ~~as the '*Rubia* group'~~ was dominant in Ag_IL, Ag_CU, Ag_CM, Ag_KA, Ag_SO, Ag_CA, and Ag_CP, and partially appeared in Ag_EL, Ag_HI, Ag_HR, Ag_EU, Ag_EJ, and Ag_CI. When *K* = 5, the genetic structure was basically similar to that at *K* = ~~5~~4, except that both Agr_YO and Agr_IX showed the (fifth) dark-blue cluster.

**Fig 4. Genetic structure of 36 HAPs of the two *gossypii* ~~group~~complex species (*A. gossypii* and *A. rhamnicola*) for 578 individuals performed by STRUCTURE 2.3.3 [69]~~(Pritchard et al. 2000)~~.** Results are shown for *K* = (2 to 5). Pop ID. (top) corresponds to Table 1, and the scientific plant name of each HAP is shown (bottom).

The Bayesian assignment tests using GENECLASS 2 (Piry et al. 2004) were carried out to identify the HAP (as population) membership of 578 individuals from all the 36 HAPs. The result of the assignment test (Table S2) indicated the average probability with which individuals were assigned to the corresponding reference HAP (as population). The self-assignment probability values (SA) averaged (0.482 ± 0.106) (mean ± s.d.) in overall HAPs,

(0.515 ± 0.103) in *A. gossypii*, and (0.427 ± 0.08) in *A. rhamnicola*. In *A. gossypii*, the mean assignment probability from 391 *A. gossypii* individuals into Ag_RH had the highest value (0.446, SA = 0.381), which was followed by the assignment value into each reference HAP of Ag_HI (0.219, SA = 0.478) and Ag_PU (0.214, SA = 0.458) (~~Fig.~~ Fig 5). In *A. rhamnicola*, the mean assignment probability from 145 *A. rhamnicola* individuals into Ar_RU had the highest value (0.137, SA = 0.489), which was similar to the assignment rate into each reference HAP of Ar_CB (0.131, SA = 0.463) and Ag_LY (0.129, SA = 0.309) (~~Fig.~~ Fig 6).

**Fig 5. Mean assignment rate (blue bar, values on left) from 391 *Aphis gossypii*** individuals into each population (x column), and self-assignment rate (orange line, values on right) of individuals of each population using GENECLASS 2 [72]~~(Piry et al. 2004).~~

**Fig 6. Mean assignment rate (blue bar, values on left) from 187 *Aphis rhamnicola*** individuals into each population (x column), and self-assignment rate (orange line, values on right) of individuals of each population using GENECLASS 2 [72]~~(Piry et al. 2004).~~

## Inferring a ~~primitive~~ancestral primary host to test hypothetical scenarios by ABC analysis

To propose the most ~~likelihood~~ likely ‘ancestral host evolution’ scenario followed by the hypothesis that most of the *A. gossypii* populations originated from two possible ancestral HAPs (e.g. Ag_RH, Ag_HI), which had diverged from *A. rhamnicola*, the ABC test was conducted. We tested four scenarios to determine which HAP is the most ancestral among all the HAPs in *A. gossypii* (see “M&M”). The generated results are presented as a logistic

**Formatted:** Font: 14 pt, Bold, Not Italic

**Formatted:** Font: 14 pt, Bold, Not Italic, Font color: Red

**Formatted:** Font color: Red

**Formatted:** Font: 14 pt, Bold, Not Italic, Font color: Red

**Formatted:** Font: 14 pt, Bold, Not Italic

**Formatted:** Font color: Red

regression using DIYABC software, estimating the PP of each tested evolutionary scenario of the hypothesis for the selected simulated data ($n_{\delta}$) (Cornuet et al. 2008), which ranged between (8 000 ~~(~~(or 6 000) and 80 000 (or 60 000)) $n_{\delta}$.

In the result of the first analysis (~~Fig.~~ Fig S4), scenario A1 obtained the highest PP ranging (0.664 ($n_{\delta}$ = 8000) to 0.697 ($n_{\delta}$ = 80 000)), with a 95 % CI of (0.601–0.727) and (0.677–0.716). Scenario A2 showed a PP (Table ~~3~~4). ~~ranging (0.007 ($n_{\delta}$ = 8 000) to 0.008 ($n_{\delta}$ = 80 000)), with a 95 % CI of (0.003–0.011) and (0.007–0.009). Scenario A3 showed a PP ranging (0.313 ($n_{\delta}$ = 8 000) to 0.290 ($n_{\delta}$ = 80 000)), with a 95 % CI of (0.251–0.375) and (0.270–0.309). Scenario A4 showed a PP ranging (0.001 ($n_{\delta}$ = 8 000) to 0.001 ($n_{\delta}$ = 80 000)), with a 95 % CI of (0.000–0.002) and (0.001–0.001). Scenario A5 showed a PP ranging (0.000 ($n_{\delta}$ = 8 000) to 0.0001 ($n_{\delta}$ = 80 000)), with a 95 % CI of (0.000–0.000) and (0.0001–0.0001). Scenario A6 showed a PP ranging (0.012 ($n_{\delta}$ = 8000) to 0.003 ($n_{\delta}$ = 80 000)), with a 95 % CI of (0.000–0.003) and (0.002–0.004). Scenario A7 showed a PP ranging (0.001 ($n_{\delta}$ = 8 000) to 0.001 ($n_{\delta}$ = 80 000)), with a 95 % CI of (0.000–0.001) and (0.001–0.001). Scenario A8 showed a PP ranging (0.0943 ($n_{\delta}$ = 8 000) to 0.0819 ($n_{\delta}$ = 80 000)), with a 95 % CI of (0.019–0.169) and (0.059–0.104).~~ As a result, scenario A1 appeared as the most robust hypothesis with the highest PP among the four scenarios tested, which suggests that, compared to the other remaining hosts, *Rhamnus* is the most ~~primitive~~ancestral host for *A. gossypii* and *A. rhamnicola*, respectively.

In the result of the second analysis (~~Fig.~~ Fig S5), ~~two~~ the scenarios ~~B1 and~~ B2 w~~ere~~as estimated more highly than the other four scenarios (Table ~~4~~3). ~~Scenario B1 showed a PP ranging (0.448 ($n_{\delta}$ = 6 000) to 0.434 ($n_{\delta}$ = 60 000)), with a 95 % CI of (0.390–0.506) and (0.453–0.537). B2 showed a PP ranging (0.520 ($n_{\delta}$ = 6 000) to 0.517 ($n_{\delta}$ = 60 000)), with a 95 % CI of (0.462–0.579) and (0.497–0.537). Scenario B3 showed a PP ranging (0.0001 ($n_{\delta}$ = 6 000) to 0.001 ($n_{\delta}$ = 60 000)), with a 95 % CI of (0.000–0.0002) and (0.0003–0.001). Scenario B4 showed a PP ranging (0.016 ($n_{\delta}$ = 6 000) to 0.021 ($n_{\delta}$ = 60 000)), with a 95 % CI of (0.007–~~

**Formatted:** Font color: Red

**Formatted:** Font color: Red

**Commented [오전4]:** Remove -> converted to Table 3

**Formatted:** Font color: Red

**Formatted:** Font color: Red

**Formatted:** Font color: Red

**Formatted:** Font color: Red

0.026) and (0.022 0.030). Scenario B5 showed a PP ranging (0.015 ($n_{♂}$ = 6 000) to 0.023 ($n_{♂}$ = 60 000)), with a 95 % CI of (0.006 0.024) and (0.019 0.026). Scenario B6 showed a PP ranging (0.0002 ($n_{♂}$ = 6 000) to 0.0001 ($n_{♂}$ = 60 000)), with a 95 % CI of (0.000 0.0007) and (0.0001 0.0001). As a result, although the direct approach estimated a slightly higher PP for scenario B1 (0.520 and 0.480) than for B2 (0.460 and 0.448) (Fig. Fig S5), the scenario B2 appeared as the the highest PP in the logistic regression. It is well supported that *A. rhamnicola* is the origin of *A. gossypii* (B1, B2), but is not conclusive whether the RED group in *A. gossypii* is diverged from the WHITE group, or vice versa.

In the result of the third analysis (Fig. Fig S6), scenario C4 obtained the highest PP (Table 43). ranging (0.361 ($n_{♂}$ = 6 000) to 0.492 ($n_{♂}$ = 60 000)), with a 95 % CI of (0.229 0.493) and (0.446 0.538). Scenario C1 showed a PP ranging (0.003 ($n_{♂}$ = 6 000) to 0.002 ($n_{♂}$ = 60 000)), with a 95 % CI of (0.0001 0.006) and (0.001 0.002). Scenario C2 showed a PP ranging (0.001 ($n_{♂}$ = 6 000) to 0.002 ($n_{♂}$ = 60 000)), with a 95 % CI of (0.000 0.001) and (0.001 0.002). Scenario C3 showed a PP ranging (0.251 ($n_{♂}$ = 6 000) to 0.202 ($n_{♂}$ = 60 000)), with a 95 % CI of (0.126 0.376) and (0.169 0.235). Scenario C5 showed a PP ranging (0.101 ($n_{♂}$ = 6 000) to 0.096 ($n_{♂}$ = 60 000)), with a 95 % CI of (0.047 0.155) and (0.079 0.114). Scenario C6 showed a PP ranging (0.283 ($n_{♂}$ = 6 000) to 0.207 ($n_{♂}$ = 60 000)), with a 95 % CI of (0.166 0.400) and (0.174 0.239). As a result, although the direct approach estimated a slightly higher PP for scenario C6 (0.400 and 0.326) and C5 (0.140 and 0.234) than for C4 (0.180 and 0.198) (Fig. Fig S6), the scenario C4 appeared as the highest PP among the four scenarios tested in the logistic regression. This suggests that, within *A. gossypii*, the WHITE group is more primitiveancestral than the MRW and RED groups, and then RED is originated from MRW, which hypothesizes that *Hibiscus* is the secondarily primary host, and can be still a refuge for the RED group.

**Commented [오전5]:** Remove -> converted to Table 3

**Formatted:** Font color: Red

**Formatted:** Font color: Red

**Formatted:** Font color: Red

**Formatted:** Font color: Red

**Formatted:** Font color: Red

**Commented [오전6]:** Remove -> converted to Table 3

**Formatted:** Font color: Red

**Formatted:** Font color: Red

**Formatted:** Font color: Red

**Table 43. Probabilities (with 95% confidence intervals in brackets) of the logistic regression for the scenarios in three different analyses inferred from DIYABC [76].** For each comparison, the selected scenario (bold entry in shaded cell) was the one with the highest probability value

| | Posterior probability of each historical scenario | | | | | |
|---|---|---|---|---|---|---|
| | First analysis (Scenario A#) | | Second analysis (Scenario B#) | | Third analysis (Scenario C#) | |
| No. | $n_\delta$ = 8000 | $n_\delta$ = 80 000 | $n_\delta$ = 8000 | $n_\delta$ = 80 000 | $n_\delta$ = 8000 | $n_\delta$ = 80 0[illegible] |
| 1 | **0.6640 [0.6010,0.7269]** | **0.6965 [0.6767,0.7162]** | 0.4480 [0.3899,0.5061] | 0.4335 [0.4142,0.4529] | 0.0029 [0.0001,0.0056] | 0.0019 [0.0013,0.[illegible] |
| 2 | 0.0072 [0.0032,0.0112] | 0.0081 [0.0069,0.0094] | **0.5207 [0.4624,0.5790]** | **0.5169 [0.4974,0.5365]** | 0.0008 [0.0000,0.0017] | 0.0018 [0.0013,0.[illegible] |
| 3 | 0.3131 [0.2511,0.3751] | 0.2899 [0.2702,0.3095] | 0.0001 [0.0000,0.0002] | 0.0005 [0.0003,0.0006] | 0.2510 [0.1258,0.3762] | 0.2017 [0.1686,0.[illegible] |
| 4 | 0.0009 [0.0000,0.0023] | 0.0007 [0.0005,0.0009] | 0.0164 [0.0065,0.0263] | 0.0264 [0.0224,0.0303] | **0.3609 [0.2288,0.4929]** | **0.4917 [0.4460,0.[illegible]** |
| 5 | 0.0000 [0.0000,0.0001] | 0.0001 [0.0001,0.0001] | 0.0146 [0.0056,0.0236] | 0.0226 [0.0192,0.0260] | 0.1011 [0.0469,0.1554] | 0.0963 [0.0786,0.[illegible] |
| 6 | 0.0124 [0.0000,0.0259] | 0.0034 [0.0024,0.0044] | 0.0002 [0.0000,0.0007] | 0.0001 [0.0001,0.0001] | 0.2834 [0.1663,0.4004] | 0.2066 [0.1742,0.[illegible] |
| 7 | 0.0005 [0.0000,0.0012] | 0.0007 [0.0005,0.0009] | N/A | N/A | N/A | N/A |
| 8 | 0.0020 [0.0000,0.0042] | 0.0007 [0.0005,0.0008] | N/A | N/A | N/A | N/A |

**Formatted:** Font color: Red
**Formatted:** Font color: Red
**Formatted:** Font color: Red
**Formatted:** Font color: Red
**Formatted:** Font color: Red
**Formatted:** Font color: Red
**Formatted:** Font color: Red
**Formatted:** Font color: Red
**Formatted:** Font color: Red
**Formatted:** Font color: Red
**Formatted:** Font color: Red
**Formatted:** Font color: Red
**Formatted:** Font color: Red

# Discussion

## Complex evolution in *Aphis gossypii*

Our results identify the genetic structure between the various primary and secondary HAPs of the two species, *A. gossypii* and *A. rhamnicola*, encompassing the most various aphid samples from wild host plants. Our population genetic analyses reveal that *A. gossypii* and *A. rhamnicola* are mainly split into ~~four~~three (~~white,~~ red, white, blue~~, dark blue~~) and ~~two~~the other three (dark-blue, blue, green) biotypes, respectively, based on the STRUCTURE result~~s~~ (~~Fig.~~ Fig 4, *K* = 5). The evolutionary trend of these aphids cannot be defined in any particular direction, and they show complex and various speciation tendencies. Here, we highlight major cases in these species.

One of the notable results is that some secondary HAPs seem to use a specific primary host (~~Fig.~~ Fig 4, *K* = 4~~3~~). In other words, *A. gossypii* and *A. rhamnicola* do not ~~randomly~~ promiscuously use their primary and secondary host plants; instead, certain biotypes use only some secondary and specific primary hosts. For example, secondary HAPs having green biotype (e.g. *Capsella*, *Lysimachia*, *Stellaria*, and *Veronica*) seem to use only *Rubia* as the primary host in our dataset. On the other hand, *Rhamanus* serves as the primary host for the secondary HAPs having blue biotypes (e.g. *Commellina*, *Leonurus*, *Perilla*, *Phryma*, and *Sedum*). These cases indicate that a group that apparently uses several primary hosts is actually a complex of groups using a specific primary host.

In contrast to the previous cases, the white and red biotypes were found to share some primary and secondary hosts (~~Fig.~~ Fig 4, *K* = 4). In particular, the white biotype has been extensively found in the most diverse primary hosts, such as *Callicarpa*, *Catalpa*, *Celastrus*, *Citrus*, *Eunonymus*, *Hibiscus*, and *Punica*. The red biotype occurs in *Citrus*, *Eunonymus*

**Formatted:** Font: 18 pt

**Formatted:** Top: 1", Bottom: 1.18"

**Formatted:** Font: 16 pt

**Formatted:** Font: 16 pt, Italic

**Formatted:** Font: Italic

*japonica*, *Hibiscus*, *Ilex*, and *Punica*. However, some primary hosts were exclusively occupied by the white (e.g. *Catalpa* and *Celastrus*,) or red type (e.g. *Ilex*), suggesting that these biotypes are possibly in a state of diverging through specialization to specific primary hosts. Interestingly, similar to the first case (i.e. blue and green types), the white and red types also tended to use specific secondary host groups, respectively. Except for a few secondary hosts (e.g. *Cucurbita* and *Solanum*), most of them represented only one biotype. For example, *Cucumis sativus* and *Capsicum annuum* were completely occupied by the red biotype. This is similar to the tendency found in most polyphagous aphids that the primary host is shared, but the secondary host is completely different [32, 81-84].

In the STRUCTURE results, the dark-blue biotype (~~Fig.~~ Fig 4, $K$ = 5) represents the third case. The dark-blue biotype was represented only by two secondary hosts, *Ixeris* and *Youngia*, and was not found in any primary host. Thus, we assume that this case seems to be an ecologically isolated host race through the loss of a primary host. Although we did not confirm the lifecycle of this biotype in this study, there is a reference to *A. gossypii* inhabiting some Asteraceae plants in the previous study, even though those HAPs are identified to *A. rhamnicola*, based on our results (Figs 3 and 4). Blackman and Eastop [22] found that populations producing eggs on the roots of *Ixeris*, including some Asteraceae plants in China identified as *A. gossypii*, may be other closely-related species.With large genetic differences from the main group of both *A. gossypii* and *A. rhamnicola* (~~Fig.~~ Fig 4), they were possibly isolated to the secondary host directly from the ~~primitive~~ancestral primary HAP in *Rhamnus* by the host alternation, supporting the possibility of differentiation from their ~~primitive~~ancestral host race according to the loss of primary host [32]. Thus, the dark-blue biotype is likely to be an ecologically ~~ecological~~incipient species of *A. rhamnicola*~~*A. gossypii*~~, ~~that~~ which has recently been derived by secondary host isolation.

**Formatted:** Font: Italic

**Formatted:** No underline

**Formatted:** Font: Italic

**Formatted:** Font color: Red

**Formatted:** Font color: Red

Our results show strong evidence of ecological specialization through a primary host shift in both *A. gossypii* and *A. rhamnicola*. ABC analyses yielded the biotypes of the two species that were formed by shifting from the shared resource, *Rhamnus*, to different primary hosts, respectively (~~Figs.~~ Figs S4 and S5). In particular, the series of primary host transitions identified in *A. gossypii* seem to have played an important role in the formation of their biotypes. For heteroecious aphids, a distinct choice of the primary host means not only utilizing different resources, but also genetic isolation between populations. This is because at one and the same time, the primary host is a resource, and a mating place. Accordingly, primary host selection in aphids is closely linked to genetic structure. Interestingly, in these species, primary host transitions occur more commonly than expected. As a traditional notion, primary hosts in aphids have been considered to be very fixed, and to not be able to easily escape, due to a highly adapted fundatrix morph [32, 33]. In particular, the white biotype that appears in many *A. gossypii* uses a wide variety of taxonomically unrelated primary hosts, which show a variable relationship between primary and fundatrix (~~Fig.~~ Fig 4). But the results of our ABC analyses identified that *A. gossypii* was firstly derived from the biotype associated with *Rhamnus* to a white biotype, and then *Hibiscus* associated biotype was derived (~~Fig.~~ Fig S4). Thus, having multiple primary hosts is possibly a transitional step to shifting to another primary host. In fact, *Hibiscus* is a plant closely related to *Gossypium* (i.e. cotton), a representative secondary host of *A. gossypii*. Unfortunately, although *Gossypium* associated population was not included in this study, it can be inferred that there is a possibility that the transition of primary host through secondary host may have occurred. However, similar to our results, Carletto, Lombaert (12) also suggested the possibility that *Hibiscus* was a shared ancestral host from which the agricultural divergence originated. in light of its HAP being genetically shared with the other HAPs in agricultural crops, such as *Gossypium*, cucurbits, and other secondary hosts.

Since the fundatrix specialization hypothesis [32, 33] has been proposed, the complex lifecycle of aphids has long been regarded as a by-product of aphid evolution. However, the identification of several heteroecious ~~races~~ HAPs in *A. gossypii* and *A. rhamnicola* in our study is largely in conflict with the expectations of this hypothesis. In our results, except for one case (i.e. the dark-blue biotype), ~~all~~ the ~~races~~HAPs appear not to be genetically isolated completely but still to be linked together ~~associated heteroecious~~between some group of primary and secondary hosts, in contrast to the assumption that monoecy as a dead-end is evolutionarily favorable over heteroecy. Moran's hypothesis predicts that the dead-end of heteroecy always leads to specialization on the secondary host by loss of the primary host. ~~However~~Nevertheless, our results indicate that a new heteroecy race can commonly be derived from the heteroecy ancestors. In other words, our results show that lifecycle evolution is not a one-way process, but can be ~~highly~~much more variable than we expected. These results are similar to the recent study on the genus *Brachycaudus* (Aphidinae: Macrosiphini), which provided strong evidence of the evolutionary lability of a complex lifecycle in *Brachycaudus* [84]~~(Jousselin et al., 2010)~~. In addition, the use of several primary hosts found in some races (i.e. red and white biotypes) negates the core assumption of the fundatrix specialization hypothesis that the fundatrix is fully adapted to the only primary host, and is inadequate to other hosts. Using multiple primary hosts is possibly a strategy for their migration success. Indeed, a migration failure can lead to high risk. For example, aphids using only a single primary host, such as *Rhopalosiphum padi*, have only a 0.6 % migration success rate [85].

## Ancestral host association in *A. gossypii* complex

*Rhamnus* appears to be the most ~~primitive~~ancestral host plant for both *A. gossypii* and *A. rhamnicola*. Several species in the *gossypii* complex group have intimate relationships with

**Formatted:** Font: 16 pt

**Formatted:** Font: 16 pt

**Formatted:** Font color: Red

*Rhamnus* (e.g. *A. frangulae*, *A. glycines*, *A. gossypii*, *A. nasturtii* and *A. rhamnicola*), which have been considered *Rhamnus* as an ancestral host for this aphid group (Blackman and Eastop, 2020; Kim et al., 2010). Our ABC result is consistent with this assumption (~~Figs.~~ Figs S4 and S5). As in the previous study, *Rhamnus* appears to serve as a shared primary host for both *A. gossypii* and *A. rhamnicola*. In the GENECLASS2 analys~~is~~es (Figs 5 and 6) as well, the assignment of most of the *gossypii* HAPs to *Rhamnus* was very high, corroborating that it was differentiated from the ancestral host, *Rhamnus*.

Despite the differentiation of aphids into various species using the different hosts (mainly secondary hosts), host utilization of *Rhamnus* still remains in several species of the *gossypii* group. The phylogenetic studies of *Aphis* showed that heteroecious species using *Rhamnus* as the primary host were derived non-consecutively from monoecious species [44, 86]. In other words, even if a monoecious species has been derived by loss of heteroecy, it seems likely to not be the dead-end of evolution [33], as well as a complete disconnect from the ancestral primary host. For example, *A. rhamnicola* and *A. gossypii* are heteroecious species, which use *Rhamnus* as the primary host, and several monoecious species on various host plants appear to have been derived between them [46]. Our ABC analyses confirmed that *Rhamnus* was lost once when branching from blue type to green type, and was then regained in white type (~~Fig.~~ Fig S4). Surprisingly, these ABC results, similarly supported by the GenClass2 results (Figs 5 and 6), almost coincided with our haplotype network results (~~Fig.~~ Fig 2).

These results ~~clearly refute~~conflict the fundatrix specialization hypothesis [32, 33], which predicts that once aphids leave the ancestral primary host, they cannot regain it again. Recently, the phylogenetic study of *Brachycaudus* demonstrated that even if they lost their potential ancestral Rosaceae hosts, they can easily regain their hosts to be the primary host for heteroecy, or the sole host for monoecy [84]. The ancestral primary host does not seem to be

an absolute being that cannot be changed due to the adaptation of the fundatrix, but seems to be a conserved resource within a specific aphid group. In fact, such a labile of aphid lifecycle related to the use of primary hosts may also occur within a species. Host alternation for some species is often not obligatory but facultative, in which the migration to the secondary host can often be omitted [15, 87]. As an example, a facultative alternation lifecycle has been reported in populations of *Aphis fabae*, even although~~which~~ ~~does~~the vast majoriy of them ~~not often~~ migrate routinely between ~~from a~~ primary ~~host to a~~and secondary hosts [87]. Although there is little known about the facultative use of the primary host in *A. gossypii*, it may be related to the primary host range expansion and lifecycle lability.

## The evidence of hybridization between *A. gossypii* and *A. rhamnicola*

Our population genetic analyses based on microsatellite and *COI* gene show that there is a significant conflict between the two results. Regardless of the primary and secondary hosts, we found individuals that are difficult to identify in some host-associated populations. *A. gossypii* and *A. rhamnicola* appeared to share major haplotypes, H9 and H2, respectively, of their counterpart species with each other. Although the PCoA and STRUCTURE results (~~Figs.~~ Figs 3 and 4) based on the microsatellites clearly showed identification of *A. gossypii*, the individuals corresponding to H2 (major haplotype of *A. rhamnicola*) were two individuals from Ag_Hi and one from Ag_KA, whereas the individuals corresponding to H9 (major haplotype of *A. gossypii*) were also identified as *A. rhamnicola*, but six from Ar_CB, one from Ar_PH ,and four from Ar_RU. Surprisingly, the cross-sharing haplotypes (H5, H9) between these two species unexpectedly contained several kinds of both primary and secondary hosts.

**Formatted:** Font color: Red

**Formatted:** Font: 16 pt

**Formatted:** Font: 16 pt, Italic

Comparing the host races between *A. gossypii* and *A. rhamnicola*, most of them were distributed in two haplotypes (H9 and H2), and they were clearly identified as distinct species, based on the microsatellite analysis (~~Fig.~~ Fig 2). However, a number of intermediate haplotypes, H13, H11, H12, H10, and H18, were observed among the species (~~Fig.~~ Fig 2). The H1 haplotype shared by several wild plant populations, such as *Ixeris*, *Leonurus*, *Perilla*, *Phryma*, and *Youngia*, is closely related to the H9 (major haplotype of *A. gossypii*). However, according to our microsatellite data, these populations appear to be closer to *A. rhamnicola* (~~Figs.~~ Figs 3 and 4). Similarly, collected from *Rubia akane*, individuals of H4, H6, H7, and H8 haplotypes apparently have alleles of *A. rhamnicola* in microsatellite data; it is most unusual that they have the haplotypes closely related to and derived from *A. gossypii*, rather than *A. rhamnicola*. In the case of H18, it is inferred to be the similar haplotype of true *A. gossypi* in Curcubitaceae, and H13 of Agr_SE, which was often cryptically recognized from *A. gossypii* as *A. rhamnicola* based on morphology and mtDNA [42, 88], was nested between the two species, *A. gossypii* and *A. rhamnicola*. Since it was reported that populations with a sexual phase on *Rubia cordifolia* in Japan appeared to be isolated from those on other primary hosts [25], it is suspected that most of the populations collected from *Rubia* in the past might actually be host races of *A. rhamnicola*, but misidentified.

**Formatted:** Font: Italic

Hybridization can have important evolutionary consequences, including speciation in association with novel host plants in insects [89]. In our study, as the two species, *A. gossypii* and *A. rhamnicola*, with distinct taxonomic and phylogenetic differences shared *COI* haplotypes of counterpart species with each other, there is a possibility of introgression by hybridization between them. These two species share overwintering (primary) host, such as *Rhamnus*, so mating and reproducing contemporarily in the same leaves or branches, because there are no physiological or ecological significant differences [43, 46]; there is therefore the possibility that hybridization occurs between them. In fact, interspecific cross mating between

sexuparae of *A. glycines*, *A. gossypii*, and *A. rhamnicola* in *Rhamnus* spp. has often been observed (unpublished data). It is an interesting phenomenon that a hybrid zone mediated not by geography, but by a resource, can exist. Lozier et al. [81] first detected hybridization and introgression between plum and almond associated *Hyalopterus* spp. on these host plants, which surprisingly were capable of feeding and developing on apricot from each species. For that possibility of hybridization, it was suggested that imperfections in any number of mechanisms associated with host plant choice [90, 91] could lead to strong selection against hybrids on parental host plants, but less so on apricot [81]. Although apricot was introduced later than other host species in the studied area, it remains a mystery why only it is able to attract all *Hyalopterus* groups and permit hybridization, whereas the other *Prunus* hosts are more restrictive [81]. Based on the phylogenetic results of *COI* or *Buchnera 16S* for *Hyalopterus* spp. [81], peach or apricot could be inferred to their ancestral host like the *Rhamnus* as a hybridization host utilized by the *gossypii* group. These results from the *gossypii* group reaffirm the hypothesis of Lozier et al. [81], which corroborates that such hybridization in the aphid group often occurs by co-existence in the primary host. However, further research is needed to determine whether a primary host (i.e. hosts that can be utilized by various host races, where they co-exist and overwinter together) are ~~primitive~~ancestral or derivational for those aphids.

**Formatted:** Font color: Red

# Supporting Information

**Formatted:** Space After: 8 pt, Line spacing: Double, Widow/Orphan control, Adjust space between Latin and Asian text, Adjust space between Asian text and numbers

**S1 ~~Fig.~~ Fig. The first eight scenarios (A1–A8) for the DIYABC analyses to infer the host evolution of the two *Aphis* species**, using a dataset that includes 578 individuals from four population groups, which consisted of

75 individuals from the ‘BLUE’ group (Ar_SE, Ar_PE, An_IX, An_YO, Ar_CO, Ar_PH, Ar_RH, Ar_LE); 90 from the ‘GREEN’ group (Ar_ST, Ar_VE, Ar_LY, Ar_CB, Ar_RU); 30 from the ‘MIXBW (BLUE+WHITE)’ group (Ag_RH, Ag_CJ); and 361 from the ‘WHITE’ group (Ag-IL, Ag_CE, Ag_EU, Ag_EJ, Ag_PU, Ag_CU, Ag_CM, Ag_KA, Ag_EL, Ag_HI, Ag_HR, Ag_FO, Ag_CI, Ag_ER, Ag_SN, Ag_CO, Ag_SO, Ag_CA, Ag_CP, Ag_CL, Ag_CT).

**S2 ~~Fig.~~ Fig. The second six scenarios (B1–B6) for the DIYABC analyses to infer the host evolution of the two *Aphis* species**, using a dataset that includes 311 individuals from four population groups, which consisted of 75 individuals from the ‘BLUE’ group (Ar_CO, Ar_PH, Ar_RH, Ar_SE, Ar_PE, Ar_LE); 90 from the ‘GREEN’ group (Ar_ST, Ar_VE, Ar_LY, Ar_CB, Ar_RU); 60 from the ‘RED’ group (Ag_IL, Ag_CU, Ag_CA, Ag_CP); and 86 from the ‘WHITE’ group (Ag_CE, Ag_FO, Ag_ER, Ag_SN, Ag_CO, Ag_CL, Ag_CT).

**S3 ~~Fig.~~ Fig. The third six scenarios (C1–C6) for the DIYABC analyses to infer the host evolution of *Aphis gossypii***, using a dataset that includes 391 individuals from four population groups except for BLUE and GREEN groups in the first and second analysis, which consisted of 30 individuals from the ‘MBW (BLUE+WHITE)’ group (Ag_RH, Ag_CJ); 207 from the ‘MRW (RED+WHITE)’ group (Ag_EU, Ag_EJ, Ag_PU, Ag_SO, Ag_CM, Ag_EL, Ag_HI, Ag_HR, Ag_CI); 68 from the ‘RED’ group (Ag-IL, Ag_CU, Ag_KA, Ag_CA, Ag_CP); and 86 from the ‘WHITE’ group (Ag_CE, Ag_FO, Ag_ER, Ag_SN, Ag_CO, Ag_CL, Ag_CT).

**S4 ~~Fig.~~ Fig. Plots output by DIYABC showing the PP (y-axis) of the first eight scenarios (A1–A8) through the direct estimate (left), and the logistic regression (right) approaches, as output by DIYABC.** The x-axis corresponds to the different $n_\delta$ values used in the computations. The results have been obtained by performing the first analysis with four scenarios.

**S5 ~~Fig.~~ Fig. Plots output by DIYABC showing the PP (y-axis) of the second six scenarios (B1–B6) through the direct estimate (left), and the logistic regression (right) approaches, as output by DIYABC.** The x-axis corresponds to the different $n_\delta$ values used in the computations. The results have been obtained by performing the first analysis with four scenarios.

**S6 ~~Fig.~~ Fig. Plots output by DIYABC showing the PP (y-axis) of the third six scenarios (C1–C6) through the direct estimate (left), and the logistic regression (right) approaches, as output by DIYABC.** The x-axis corresponds to the different $n_\delta$ values used in the computations. The results have been obtained by performing the first analysis with four scenarios.

**S1 Table. Collection data for 578 aphids analyzed in this study.** † possibly *A. rhamnicola* or other cryptic species †† possibly other cryptic species

**S2 Table. Mean assignment rate of individuals into (rows) and from (columns) each population using GeneClass 2 (Piry et al. 2004).** Values in bold indicate the proportions of individuals assigned to the source population (self-assignment). Values less than 0.001 were excluded from the table

**S3 Table. Probabilities (with 95% confidence intervals in brackets) of the logistic regression for the scenarios in three different analyses inferred from DIYABC**. For each comparison, the selected scenario (bold entry in shaded cell) was the one with the highest probability value.

# References

Formatted: Font: 18 pt, Bold

Formatted: Indent: Left: 0", First line: 0 ch, Line spacing: Multiple 1.08 li


1. Chapman R. Foraging and food choice in phytophagous insects. In: Hardege JD, editor. Chemical ecology. Oxford: Eolss Publishers; 2009. p. 99-141.
2. Berlocher SH, Feder JL. Sympatric speciation in phytophagous insects: Moving beyond controversy? Annual Review of Entomology. 2002;47:773-815. PubMed PMID: ISI:000173421900025.
3. Jaenike J. Host Specialization in Phytophagous Insects. Annual Review of Ecology and Systematics. 1990;21(1):243-73. doi: 10.1146/annurev.es.21.110190.001331.
4. Mitter C, Farrell B, Wiegmann B. The phylogenetic study of adaptive zones: Has phytophagy promoted insect diversification? The American Naturalist. 1988;132(1):107-28.
5. Futuyma DJ, Agrawal AA. Macroevolution and the biological diversity of plants and herbivores. Proceedings of the National Academy of Sciences of the United States of America. 2009;106(43):18054-61. doi: DOI 10.1073/pnas.0904106106. PubMed PMID: ISI:000271222500006.
6. Mitter C, Farrell B, Futuyma D. Phylogenetic studies of insect-plant interactions: Insights into the genesis of diversity. Trends in Ecology and Evolution. 1991;6:290-3.
7. Ferrari J, Via S, Godfray HC. Population differentiation and genetic variation in performance on eight hosts in the pea aphid complex. Evolution. 2008;62(10):2508-24. doi: 10.1111/j.1558-5646.2008.00468.x. PubMed PMID: 18647340.
8. Lopez-Vaamonde C, Rasplus JY, Weiblen GD, Cook JM. Molecular phylogenies of fig wasps: Partial cocladogenesis of pollinators and parasites. Molecular Phylogenetics and Evolution. 2001;21(1):55-71. doi: DOI 10.1006/mpev.2001.0993. PubMed PMID: ISI:000171486700006.
9. Walsh BD. On phytophagic varieties and phytophagic species. Proc Entomol Soc Phila. 1864;3:403-30.
10. Matsubayashi KW, Ohshima I, Nosil P. Ecological speciation in phytophagous insects. Entomologia Experimentalis et Applicata. 2010;134(1):1-27. doi: DOI 10.1111/j.1570-7458.2009.00916.x. PubMed PMID: ISI:000272308300001.
11. Peccoud J, Ollivier A, Plantegenest M, Simon JC. A continuum of genetic divergence from sympatric host races to species in the pea aphid complex. Proceedings of the National Academy of Sciences of the United States of America. 2009;106(18):7495-500. PubMed PMID: ISI:000265783600043.
12. Carletto J, Lombaert E, Chavigny P, Brevault T, Lapchin L, Vanlerberghe-Masutti F. Ecological specialization of the aphid *Aphis gossypii* Glover on cultivated host plants. Molecular Ecology. 2009;18(10):2198-212. doi: DOI 10.1111/j.1365-294X.2009.04190.x. PubMed PMID: WOS:000265774300013.
13. Margaritopoulos JT, Malarky G, Tsitsipis JA, Blackman RL. Microsatellite DNA and behavioural studies provide evidence of host-mediated speciation in *Myzus persicae* (Hemiptera : Aphididae). Biological Journal of the Linnean Society. 2007;91(4):687-702. doi: DOI 10.1111/j.1095-8312.2007.00828.x. PubMed PMID: WOS:000248599900011.
14. Simon JC, Carre S, Boutin M, Prunier-Leterme N, Sabater-Muñoz, Latorre A, et al. Host-based divergence in populations of the pea aphid: insights from nuclear markers and the prevalence of facultative symbionts. Proceedings of the Royal Entomological Society of London (B). 2003;270:1703-12.
15. Peccoud J, Simon JC, von Dohlen C, Coeur d'acier A, Plantegenest M, Vanlerberghe-Masutti F, et al. Evolutionary history of aphid-plant associations and their role in aphid diversification. C R Biol. 2010;333(6-7):474-87. doi: 10.1016/j.crvi.2010.03.004. PubMed PMID: 20541159.
16. Kergoat GJ, Meseguer AS, Jousselin E. Chapter Two - Evolution of Plant–Insect Interactions: Insights From Macroevolutionary Approaches in Plants and Herbivorous Insects. In: Sauvion N, Thiéry D, Calatayud P-A, editors. Advances in Botanical Research. 81: Academic Press; 2017. p. 25-53.
17. Caillaud MC, Via S. Specialized feeding behavior influences both ecological specialization and assortative mating in sympatric host races of pea aphids. The American Naturalist. 2000;156(6):606-21. PubMed PMID: ISI:000165870000004.
18. Via S, Bouck AC, Skillman S. Reproductive isolation between divergent races of pea aphids on two hosts. II. Selection against migrants and hybrids in the parental environments. Evolution. 2000;54(5):1626-37. PubMed PMID: ISI:000165471000015.
19. Kanbe T, Akimoto SI. Allelic and genotypic diversity in long-term asexual populations of the pea aphid, *Acyrthosiphon pisum* in comparison with sexual populations. Molecular Ecology. 2009;18(5):801-16. doi: DOI 10.1111/j.1365-294X.2008.04077.x. PubMed PMID: WOS:000263521800006.
20. Moran NA, Kaplan ME, Gelsey MJ, Murphy TG, Scholes EA. Phylogenetics and evolution of the aphid genus *Uroleucon* based on mitochondrial and nuclear DNA sequences. Systematic Entomology. 1999;24(1):85-93. PubMed PMID: ISI:000078781800006.
21. Favret C, Voegtlin DJ. Speciation by host-switching in pinyon Cinara (Insecta: Hemiptera: Aphididae). Mol Phylogenet Evol. 2004;32(1):139-51. doi: 10.1016/j.ympev.2003.12.005. PubMed PMID: 15186803.

22. Blackman RL, Eastop VF. Aphids on the World's Plants. Available from: http://www.aphidsonworldsplant.info. 2019;(retrieval date 08/05/2019).
23. Inaizumi M. Studies on the life-cycle and polymorphism of *Aphis gossypii* Glover (Homoptera, Aphididae).) Special Bulletin of the College of Agriculture, Utsunomiya University. 1980;37(1-132).
24. Lee S, Kim H. Economic Insects of Korea 28 (Insecta Koreana Suppl. 35), Aphididae: Aphidini (Hemiptera: Sternorrhyncha). Suwon, Rep. of Korea: National Institue of Agricultural Science and Technology; 2006.
25. Inaizumi M. Life cycle of *Aphis gossypii* Glover (Homoptera, Aphididae) with special reference to biotype differentiation on various host plants. Kontyû. 1981;49:219-40.
26. Heie OE. The Aphidoidea (Hemiptera) of Fennoscandia and Denmark. III. Family Aphididae: subfamily Pterocommatinae & tribe Aphidini of subfamily Aphidinae. Klampenborg, Denmark: Scandinavian Science Press Ltd.; 1986.
27. Kring JB. The life cycle of the melon aphid, *Aphis gossypii* Glover, an example of facultative migration. Annals of the Entomological Society of America. 1959;52:284-6.
28. Blackman RL, Eastop VF. Aphids on the World's Herbaceous Plants and Shrubs, Vol. 2, The Aphids. Chichester: John Wiley & Sons Ltd.; 2006. 1025-439 p.
29. Eastop VF, Hille Ris Lambers D. Survey of the World's Aphids: Dr. W. Junk, The Hague (The Netherlands); 1976.
30. Favret C. Aphid Species File. Version 1.0/4.0. Available at: http://Aphid.SpeciesFile.org. Accessed 2019 Aug 6. 2019.
31. Moran NA. Adaptation and Constraint in the Complex Life-Cycles of Animals. Annual Review of Ecology and Systematics. 1994;25:573-600. doi: DOI 10.1146/annurev.ecolsys.25.1.573. PubMed PMID: WOS:A1994PU88300023.
32. Moran NA. The Evolution of Aphid Life-Cycles. Annual Review of Entomology. 1992;37:321-48. doi: DOI 10.1146/annurev.ento.37.1.321. PubMed PMID: WOS:A1992GY50200014.
33. Moran NA. The Evolution of Host-Plant Alternation in Aphids - Evidence for Specialization as a Dead End. Am Nat. 1988;132(5):681-706. doi: Doi 10.1086/284882. PubMed PMID: WOS:A1988R012000006.
34. von Dohlen CD, Rowe CA, Heie OE. A test of morphological hypotheses for tribal and subtribal relationships of Aphidinae (Insecta : Hemiptera : Aphididae) using DNA sequences. Molecular Phylogenetics and Evolution. 2006;38(2):316-29. doi: DOI 10.1016/j.ympev.2005.04.035. PubMed PMID: WOS:000235121800003.
35. Blackman RL, Eastop VF. Aphids on the World's Trees: An Identification and Information Guide. Wallingford: CAB International; 1994. 987 p.
36. Dixon AFG. The way of life of aphids: host specificity, speciation and distribution. In: Minks AK, Harrewijn P, editors. Aphids their biology, natural enemies and control, Vol A. A. Amsterdam: Elsevier; 1987. p. 197-207.
37. Ward SA. Reproduction and host selection by aphids: the importance of ''rendezvous'' hosts. In: Bailey WJ, Ridshill-Smith J, editors. Reproductve Behaviour of Insects. New york: Chapman and Hall; 1991. p. 202-26.
38. Blackman RL, Eastop VF. Aphids on the World's Crops: An Identification and Information Guide. 2nd ed. Chichester: John Wiley & Sons Ltd.; 2000. 466 p.
39. Hardy NB, Peterson DA, von Dohlen CD. The evolution of life cycle complexity in aphids: Ecological optimization or historical constraint? Evolution. 2015;69(6):1423-32. doi: 10.1111/evo.12643.
40. Moran NA. Aphid Life-Cycles - 2 Evolutionary Steps. Am Nat. 1990;136(1):135-8. doi: Doi 10.1086/285087. PubMed PMID: WOS:A1990DY11500009.
41. Charaabi K, Carletto J, Chavigny P, Marrakchi M, Makni M, Vanlerberghe-Masutti F. Genotypic diversity of the cotton-melon aphid *Aphis gossypii* (Glover) in Tunisia is structured by host plants. Bulletin of Entomological Research. 2008;98(4):333-41. doi: Doi 10.1017/S0007485307005585. PubMed PMID: WOS:000259233700002.
42. Cocuzza G, Cavalieri V, Barbagallos S. Preliminary results in the taxonomy of the cryptic group *Aphis frangulae*/*gossypii* obtained from mitochondrial DNA sequences. Bulletin of Insectology. 2008;61(1):125-6.
43. Kim H, Hoelmer KA, Lee W, Kwon YD, Lee S. Molecular and morphological identification of the soybean aphid and other *Aphis* species on the primary host *Rhamnus davurica* in Asia. Annals of the Entomological Society of America. 2010;103(4):532-43. doi: Doi 10.1603/An09166. PubMed PMID: ISI:000279548300008.
44. Kim H, Lee S, Jang Y. Macroevolutionary patterns in the Aphidini aphids (Hemiptera: Aphididae): diversification, host association, and biogeographic origins. PLoS One. 2011;6(9):e24749. doi: 10.1371/journal.pone.0024749. PubMed PMID: 21935453; PubMed Central PMCID: PMCPMC3174202.
45. Richardson JE, Chatrou LW, Mols JB, Erkens RHJ, Pirie MD. Historical biogeography of two cosmopolitan families of flowering plants: Annonaceae and Rhamnaceae. Philos T R Soc B. 2004;359(1450):1495-508. doi: DOI 10.1098/rstb.2004.1537. PubMed PMID: ISI:000224918700005.

46. Lee Y, Lee W, Lee S, Kim H. A cryptic species of *Aphis gossypii* (Hemiptera: Aphididae) complex revealed by genetic divergence and different host plant association. Bulletin of Entomological Research. 2015;105(1):40-51. Epub 11/21. doi: 10.1017/S0007485314000704.
47. Carletto J, Blin A, Vanlerberghe-Masutti F. DNA-based discrimination between the sibling species *Aphis gossypii* Glover and *Aphis frangulae* Kaltenbach. Systematic Entomology. 2009;34(2):307-14. PubMed PMID: ISI:000264374300008.
48. Foottit RG, Maw HEL, Havill NP, Ahern RG, Montgomery ME. DNA barcodes to identify species and explore diversity in the Adelgidae (Insecta: Hemiptera: Aphidoidea). Molecular Ecology Resources. 2009;9:188-95. PubMed PMID: ISI:000265227700019.
49. Hebert PDN, Cywinska A, Ball SL, DeWaard JR. Biological identifications through DNA barcodes. Proceedings of the Royal Society B-Biological Sciences. 2003;270(1512):313-21. doi: DOI 10.1098/rspb.2002.2218. PubMed PMID: WOS:000181064200013.
50. Lee W, Kim H, Lim J, Choi HR, Kim Y, Kim YS, et al. Barcoding aphids (Hemiptera: Aphididae) of the Korean Peninsula: updating the global data set. Mol Ecol Resour. 2011;11(1):32-7. doi: 10.1111/j.1755-0998.2010.02877.x. PubMed PMID: 21429098.
51. Katoh K, Rozewicki J, Yamada KD. MAFFT online service: multiple sequence alignment, interactive sequence choice and visualization. Briefings in Bioinformatics. 2017;20(4):1160-6. doi: 10.1093/bib/bbx108.
52. Rozas J, Ferrer-Mata A, Sánchez-DelBarrio JC, Guirao-Rico S, Librado P, Ramos-Onsins SE, et al. DnaSP 6: DNA Sequence Polymorphism Analysis of Large Data Sets. Molecular Biology and Evolution. 2017;34(12):3299-302. doi: 10.1093/molbev/msx248.
53. Bandelt H-J, Forster P, Röhl A. Median-joining networks for inferring intraspecific phylogenies. Molecular biology and evolution. 1999;16:37-48.
54. Andree K, Axtner J, Bagley MJ, Barlow EJ, Beebee TJC, Bennetzen JL, et al. Permanent Genetic Resources added to Molecular Ecology Resources Database 1 April 2010-31 May 2010. Molecular Ecology Resources. 2010;10(6):1098-105. doi: DOI 10.1111/j.1755-0998.2010.02898.x. PubMed PMID: ISI:000282876300023.
55. Vanlerberghe-Masutti F, Chavigny P, Fuller SJ. Characterization of microsatellite loci in the aphid species *Aphis gossypii* Glover. 1999.
56. Wilson ACC, Massonnet B, Simon JC, Prunier-Leterme N, Dolatti L, Llewellyn KS, et al. Cross-species amplification of microsatellite loci in aphids: assessment and application. Molecular Ecology Notes. 2004;4(1):104-9. doi: DOI 10.1046/j.1471-8286.2003.00584.x. PubMed PMID: WOS:000189159500032.
57. Michel AP, Zhang W, Jung JK, Kang ST, Mian MAR. Cross-Species Amplification and Polymorphism of Microsatellite Loci in the Soybean Aphid, Aphis glycines. J Econ Entomol. 2009;102(3):1389-92. PubMed PMID: ISI:000266641700068.
58. Peakall R, Smouse PE. GenAlEx 6.5: genetic analysis in Excel. Population genetic software for teaching and research--an update. Bioinformatics. 2012;28(19):2537-9. doi: 10.1093/bioinformatics/bts460. PubMed PMID: 22820204; PubMed Central PMCID: PMCPMC3463245.
59. Goudet J. FSTAT, A Program to Estimate and Test Gene Diversities and Fixation Indices (version 2.9.3.2). Institute of Ecology, University of Lausanne, Switzerland. Available from http//www.unil.ch/izea/softwares/fstat.html. 2002.
60. Raymond M, Rousset F. Genepop (version-1.2) - population genetics software for exact tests and ecumenicism. J Hered. 1995;86(3):248-9. PubMed PMID: ISI:A1995RB30200017.
61. Rice WR. Analyzing tables of statistical tests. Evolution. 1989;43(1):223-5. doi: Doi 10.2307/2409177. PubMed PMID: WOS:A1989R828900018.
62. Sunnucks P, DeBarro PJ, Lushai G, Maclean N, Hales D. Genetic structure of an aphid studied using microsatellites: Cyclic parthenogenesis, differentiated lineages and host specialization. Molecular Ecology. 1997;6(11):1059-73. doi: DOI 10.1046/j.1365-294X.1997.00280.x. PubMed PMID: WOS:A1997YJ88300006.
63. Oosterhout Cv, Hutchinson WF, Wills DPM, Shipley P. micro-checker: software for identifying and correcting genotyping errors in microsatellite data. Molecular Ecology Notes. 2004;4(3):535-8.
64. Brookfield JFY. A simple new method for estimating null allele frequency from heterozygote deficiency. Molecular Ecology. 1996;5(3):453-5. doi: 10.1046/j.1365-294X.1996.00098.x.
65. Excoffier L, Lischer HE. Arlequin suite ver 3.5: a new series of programs to perform population genetics analyses under Linux and Windows. Mol Ecol Resour. 2010;10(3):564-7. doi: 10.1111/j.1755-0998.2010.02847.x. PubMed PMID: 21565059.
66. Weir BS, Cockerham CC. Estimating F-statistics for the analysis of population structure. Evolution. 1984;38(6):1358-70. doi: Doi 10.2307/2408641. PubMed PMID: WOS:A1984TY40400017.
67. Excoffier L, Laval G, Schneider S. Arlequin (version 3.0): An integrated software package for population genetics data analysis. Evol Bioinform. 2005;1:47-50. PubMed PMID: ISI:000207065900004.
68. Excoffier L, Smouse PE, Quattro JM. Analysis of molecular variance inferred from metric distances

among DNA haplotypes - application to human mitochondrial DNA restriction data. Genetics. 1992;131(2):479-91. PubMed PMID: WOS:A1992HW75900021.

69. Pritchard JK, Stephens M, Donnelly P. Inference of population structure using multilocus genotype data. Genetics. 2000;155(2):945-59. PubMed PMID: WOS:000087475100039.

70. Evanno G, Regnaut S, Goudet J. Detecting the number of clusters of individuals using the software STRUCTURE: A simulation study. Molecular Ecology. 2005;14(8):2611-20. doi: DOI 10.1111/j.1365-294X.2005.02553.x. PubMed PMID: WOS:000229961500029.

71. Rosenberg NA. distruct: a program for the graphical display of population structure. Molecular Ecology Notes. 2004;4(1):137-8. doi: 10.1046/j.1471-8286.2003.00566.x.

72. Piry S, Alapetite A, Cornuet JM, Paetkau D, Baudouin L, Estoup A. GENECLASS2: A software for genetic assignment and first-generation migrant detection. J Hered. 2004;95(6):536-9. doi: DOI 10.1093/jhered/esh074. PubMed PMID: ISI:000224482500012.

73. Rannala B, Mountain JL. Detecting immigration by using multilocus genotypes. Proceedings of the National Academy of Sciences of the United States of America. 1997;94(17):9197-201. doi: DOI 10.1073/pnas.94.17.9197. PubMed PMID: WOS:A1997XR76500053.

74. Cornuet JM, Santos F, Beaumont MA, Robert CP, Marin JM, Balding DJ, et al. Inferring population history with DIY ABC: a user-friendly approach to approximate Bayesian computation. Bioinformatics. 2008;24(23):2713-9. doi: DOI 10.1093/bioinformatics/btn514. PubMed PMID: WOS:000261168600008.

75. Estoup A, Guillemaud T. Reconstructing routes of invasion using genetic data: why, how and so what? Mol Ecol. 2010;19(19):4113-30. doi: DOI 10.1111/j.1365-294X.2010.04773.x. PubMed PMID: WOS:000282180500005.

76. Cornuet JM, Ravigne V, Estoup A. Inference on population history and model checking using DNA sequence and microsatellite data with the software DIYABC (v1.0). Bmc Bioinformatics. 2010;11. doi: Artn 401 Doi 10.1186/1471-2105-11-401. PubMed PMID: WOS:000281442400001.

77. Portnoy DS, Hollenbeck CM, Belcher CN, Driggers Iii WB, Frazier BS, Gelsleichter J, et al. Contemporary population structure and post-glacial genetic demography in a migratory marine species, the blacknose shark, Carcharhinus acronotus. Molecular Ecology. 2014;23(22):5480-95. doi: 10.1111/mec.12954.

78. Cabrera AA, Palsbøll PJ. Inferring past demographic changes from contemporary genetic data: A simulation-based evaluation of the ABC methods implemented in diyabc. Molecular Ecology Resources. 2017;17(6):e94-e110. doi: 10.1111/1755-0998.12696.

79. Techer MA, Clémencet J, Turpin P, Volbert N, Reynaud B, Delatte H. Genetic characterization of the honeybee (Apis mellifera) population of Rodrigues Island, based on microsatellite and mitochondrial DNA. Apidologie. 2015;46(4):445-54. doi: 10.1007/s13592-014-0335-9.

80. Bandelt HJ, Forster P, Röhl A. Median-joining networks for inferring intraspecific phylogenies. Molecular Biology and Evolution. 1999;16(1):37-48. doi: 10.1093/oxfordjournals.molbev.a026036.

81. Lozier JD, Roderick GK, Mills NJ. Genetic evidence from mitochondrial, nuclear, and endosymbiont markers for the evolution of host plant associated species in the aphid genus *Hyalopterus* (Hemiptera : Aphididae). Evolution. 2007;61(6):1353-67. doi: DOI 10.1111/j.1558-5646.2007.00110.x. PubMed PMID: WOS:000247125100009.

82. Margaritopoulos JT, Tsitsipis JA, Goudoudaki S, Blackman RL. Life cycle variation of *Myzus persicae* (Hemiptera: Aphididae) in Greece. Bulletin of Entomological Research. 2002;92(4):309-19. Epub 2002/08/23. doi: 10.1079/BER2002167. PubMed PMID: 12191439.

83. Guldemond JA. Host Plant Relationships and Life-Cycles of the Aphid Genus *Cryptomyzus*. Entomologia Experimentalis Et Applicata. 1991;58(1):21-30. PubMed PMID: WOS:A1991EZ54000003.

84. Jousselin E, Genson G, Coeur d'Acier A. Evolutionary lability of a complex life cycle in the aphid genus *Brachycaudus*. BMC evolutionary biology. 2010;10(1):295. doi: 10.1186/1471-2148-10-295. PubMed PMID: 20920188; PubMed Central PMCID: PMCPMC2958166.

85. Ward SA, Leather SR, Pickup J, Harrington R. Mortality during dispersal and the cost of host-specificity in parasites:how many aphids find hosts? Journal of Animal Ecology. 1998;67:763-73.

86. Coeur d'Acier A, Jousselin E, Martin JF, Rasplus JY. Phylogeny of the genus *Aphis* Linnaeus, 1758 (Homoptera : Aphididae) inferred from mitochondrial DNA sequences. Molecular Phylogenetics and Evolution. 2007;42(3):598-611. doi: DOI 10.1016/j.ympev.2006.10.006. PubMed PMID: WOS:000245717300003.

87. Mackenzie A. A trade-off for host plant utilization in the black bean aphid, *Aphis fabae*. Evolution. 1996;50(1):155-62. doi: Doi 10.2307/2410790. PubMed PMID: WOS:A1996TX89100016.

88. Lagos-Kutz DM, Favret C, Giordano R, Voegtlin D. Molecular and morphological differentiation between *Aphis gossypii* Glover (Hemiptera, Aphididae) and related species, with particular reference to the North American Midwest. ZooKeys. 2014;459:49-72.

89. Taylor SA, Larson EL. Insights from genomes into the evolutionary importance and prevalence of hybridization in nature. Nature Ecology & Evolution. 2019;3(2):170-7. doi: 10.1038/s41559-018-0777-y.

90. Dixon AFG. Aphid Ecology–An optimization approach. Norwell: Kluwer Academic Pub; 1998.

91. Powell G, Tosh CR, Hardie J. Host plant selection by aphids: behavioral, evolutionary, and applied perspectives. Annu Rev Entomol. 2006;51:309-30. doi: 10.1146/annurev.ento.51.110104.151107. PubMed PMID: 16332214.

[1]

Reviewer 1: The potential effects of incomplete lineage sorting should not be ignored in the Discussion. You should consider stating that incomplete lineage sorting cannot be discounted as a cause before revealing that interspecific matings have been observed (which gives more credence to the hybridization hypothesis).

Editor: In other respects, this revision failed to address justified criticism by the reviewers. Reviewer 2 made the perfectly valid point that shared mitochondrial haplotypes between *A. gossypii* and *A. rhamnicola* (and between other potential species showing strong differentiation with nuclear markers) could be the result of incomplete lineage sorting rather than evidence of ongoing hybridization. I cannot judge which explanation is more likely, and the authors have every right to discuss why they consider hybridization more likely based on the available evidence. But this is not done in the paper. There is some unconvincing rebuttal in the cover letter and the term incomplete lineage sorting does not even show up in the paper's Discussion. This is not thorough.

>>
[Response and rebuttal]

We have reflected in the manuscript what the reviewer and editor pointed out. As pointed out by reviews comments, doing lineage sorting by *COI* haplotype results is likely unclear at the first submitted manuscript. Since *A. gossypii* and *A. rhamnicola* cross-sharing haplotypes in several host plants, there is a problem with lineage sorting or DNA identification with only *COI* haplotypes (barcoding). However, I am not saying clearly that our sample is misidentified with the two species, *A. gossypii* and *A. rhamnicola*. In particular, it was revealed that genotyping by microsatellite was the only way to distinguish between these two evolutionarily intermediate species, even for organizing the sampled individuals in HAP type and utilizing them for analysis.

Accepting these editor and reviewer's opinions, the identification content by *COI* haplotype (barcoding) was deleted and supplemented with the concept of species lineage (group) sorting instead. Therefore, 'Groups' sorted by lineage in the table are specified and subdivided into *A. gossypii* Group1, *A. g.* Group 2, *A. rhamnicola* Group 1, *A. r.* Group 2, *A. r.* Group 3. In addition, according to our new lineage sorting, species names were deleted in Fig 2 and Fig 4. since specifying the species creates a rather confusing situation and misunderstanding of original purpose of study. The changes in the text are as follows.

"The two *Aphis species, A. gossypii* and *A. rhamnicola*, we study here are not only very similar in morphology, but also share several host plants due to the polyphagy. Although we performed species identification through morphology and host plant relationships as a first step and also tested DNA barcoding for all individuals collected on their shared host plants (eg *Capsella*, *Rhamnus*, and *Rubia*), we found that there were a lot of the haplotypes cross-shared between *A. gossypii* and *A. rhamnicola* (see Results). Therefore, instead of identifying the species with 36 HAPs, we applied the dominant assignment (white, green, blue, red, dark blue) of the genetic structure (K =3, 4, 5) by STRUCTURE as well as the PCoA results (see Results) to sort their lineags into five groups as *Aphis gossypii* Group 1, A. g. Group 2, *A. rhmanicola* Group 1, A. r. Group 2 and A. r. Group 3 (Table 1). '*Aphis gossypii*' and'A. *ramincola*', which are mentioned later, are meant to include all group lin eages containing the HAPs assigned by the results. Table S3 shows detailed information for lineage sorted samples used in DNA analyses." **-> LINE 202-211, Table 1, Fig2, Fig4 revised**

In addition, the content of HAP mixing in the intermediating host plant based on *COI* or *16S* hybridization is sufficiently mentioned in the discussion by citing a paper by Lozier et al. [89]. Please check the contents at the end of the discussion. In relation to *COI* hybridization, hybridized population of *Hyalopterus pruni* has already found by Lozier et al. [89]. It has been explored and studied by and presented the second observation in aphids and corroborating results in our study. It is emphasized once again that the phenomenon of cross-sharing of *COI* haplotypes between the two species is by no means due to errors in lineage sorting.

[2]

Reviewer 1 continues to be concerned with over- or mis-interpretation of your population genetics data. You should address the following points in your revision:

The conclusions you draw about the cause of heterozygote excess or heterozygote deficit (lines 455-464) are not appropriate for the Results section. If retained, this section should be moved to the Discussion, and the comments of Reviewer 1 addressed.

Editor: Similarly, reviewer 1 criticized the misleading interpretation of some standard population genetic indices (heterozygosities etc.). While the authors did check whether some of the deviations from HWE might be due to the inclusion of multiple clonal copies of the same genotypes within populations, the (over-)interpreted summary of the results remained completely unchanged in the paper. For example, this part:

"Heterozygote excess in Ag_CA, Ag_CP, and Ar_PE were likely the result of heterosis or over-dominance related to selection preference toward heterozygous combination [81], or fixation of heterozygous genotypes; and, correspondingly, negative FIS values also showed an increase in heterozygosity that was generally due to random mating or outbreeding [82]. In contrast, heterozygote deficit (i.e., homozygote excess) in Ag_CJ, Ar_YO, Ar_PH, and Ar_RU was likely caused by retaining numerous unique genotypes with private alleles within a population, and positive FIS values explained that the amount of heterozygous offspring in the population decreased, usually due to inbreeding [82]."

This is scientifically unsound in several respects. Without other, independent evidence it is simply not possible to infer heterosis as the cause of heterozygote excess at neutral markers.
Random mating will restore HWE and not lead to "an increase in heterozygosity". Etc.
>>
[Response and rebuttal]

Because aphids 'parthenogenetically' reproduce in their secondary host, especially under anholocyclic life, the result of heterosis or over-dominance related to selection preference toward heterozygous combination or fixation of heterozygous genotypes have been commonly reported in study of aphids. This is far from the normal reproductive situation under random mating on other diploid organisms. We cited additional references. Please see citations below:

81. Delmotte F, Sabater-Munoz B, Prunier-Leterme N, Latorre A, Sunnucks P, Rispe C, et al. Phylogenetic evidence for hybrid origins of asexual lineages in an aphid species. Evolution. 2003;57(6):1291-303. doi: Doi 10.1554/02-557. PubMed PMID: WOS:000183997400007.
82. Simon JC, Baumann S, Sunnucks P, Hebert PDN, Pierre JS, Le Gallic JF, et al. Reproductive mode and population genetic structure of the cereal aphid Sitobion avenae studied using phenotypic and microsatellite markers. Molecular Ecology. 1999;8(4):531-45. doi: DOI 10.1046/j.1365-294x.1999.00583.x. PubMed PMID: WOS:000080177700003.
83. Delmotte F, Leterme N, Gauthier JP, Rispe C, Simon JC. Genetic architecture of sexual and asexual populations of the aphid *Rhopalosiphum padi* based on allozyme and microsatellite markers. Molecular Ecology. 2002;11(4):711-23. doi: DOI 10.1046/j.1365-294X.2002.01478.x. PubMed PMID: WOS:000175250300007.
84. Wilson ACC, Sunnucks P, Blackman RL, Hales DF. Microsatellite variation in cyclically parthenogenetic populations of *Myzus persicae* in south-eastern Australia. Heredity. 2002;88(4):258-66. doi: 10.1038/sj.hdy.6800037.

In the respects to the description of heterozygote deficit, in the contrary to parthenogenetic life, sexual lineages generally show the heterozygote deficits [81-84]. However, as you pointed out, I cannot connect it without confirming clear evidence of correlation between sexual lineages and ecology of HAPs sampled from our study, thus deleted it (explanation of heterozygote deficits. Finally, the sentences were amended as below:

"Heterozygote excess in Ag_CA, Ag_CP, and Ar_PE were likely the result of heterosis or over-dominance related to selection preference toward heterozygous combination or fixation of heterozygous genotypes due to parthenogenesis of aphids in secondary host, especially under anholocyclic (permanently asexual) life [81]. Similar to our results, this phenomenon was already reported from several aphid species such as *Sitobion*

*avenae, Myzus persicae* and *Rhopalosiphum padi* having permanently or temporary asexual life, which showed the significant heterozygote excess [82-84]. Negative *FIS* values also showed an increase in heterozygosity that was generally due to random mating or outbreeding, whereas positive *FIS* values explained that the amount of heterozygous offspring in the population decreased, usually due to inbreeding [85]." **-> LINE 459-466 revised**

Unlike the reviewer's request, because we do not treat discussion of the heterozygote excess in Ag_CA, Ag_CP, and Ar_PE, we still remain these contents in the results section in view of consistency of manuscript content.

[3]

You might also consider:
Providing a citation and/or example species in support of the statement in lines 632-634.

Add the related references to support the statement:

"This is similar to the tendency found in most polyphagous aphids that the primary host is shared, but the secondary host is completely different [32, 86-89]."


32. Moran NA. The Evolution of Aphid Life-Cycles. Annual Review of Entomology. 1992;37:321-48. doi: DOI 10.1146/annurev.ento.37.1.321. PubMed PMID: WOS:A1992GY50200014.

86. Lozier JD, Roderick GK, Mills NJ. Genetic evidence from mitochondrial, nuclear, and endosymbiont markers for the evolution of host plant associated species in the aphid genus *Hyalopterus* (Hemiptera : Aphididae). Evolution. 2007;61(6):1353-67. doi: DOI 10.1111/j.1558-5646.2007.00110.x. PubMed PMID: WOS:000247125100009.

87. Margaritopoulos JT, Tsitsipis JA, Goudoudaki S, Blackman RL. Life cycle variation of *Myzus persicae* (Hemiptera: Aphididae) in Greece. Bulletin of Entomological Research. 2002;92(4):309-19. Epub 2002/08/23. doi: 10.1079/BER2002167
S0007485302000366 [pii]. PubMed PMID: 12191439.

88. Guldemond JA. Host Plant Relationships and Life-Cycles of the Aphid Genus *Cryptomyzus*. Entomologia Experimentalis Et Applicata. 1991;58(1):21-30. PubMed PMID: WOS:A1991EZ54000003.

89. Jousselin E, Genson G, Coeur d'Acier A. Evolutionary lability of a complex life cycle in the aphid genus *Brachycaudus*. BMC evolutionary biology. 2010;10(1):295. doi: 10.1186/1471-2148-10-295. PubMed PMID: 20920188; PubMed Central PMCID: PMCPMC2958166.
