# Supplementary material for: Complex evolution in Aphis gossypii group (Hemiptera: Aphididae), evidence of primary host shift and hybridization between sympatric species
Source: PLoS One. 2021 Feb 4;16(2):e0245604. doi: 10.1371/journal.pone.0245604 (PMC7861460; doi:10.1371/journal.pone.0245604)
Supplement: Attachment — Submitted filename: !+Responses to Reviewers comments_201231_PONE_2st REVISION.docx [file pone.0245604.s011.docx]

[1]

Reviewer 1: The potential effects of incomplete lineage sorting should not be ignored in the Discussion.  You should consider stating that incomplete lineage sorting cannot be discounted as a cause before revealing that interspecific matings have been observed (which gives more credence to the hybridization hypothesis).

Editor: In other respects, this revision failed to address justified criticism by the reviewers. Reviewer 2 made the perfectly valid point that shared mitochondrial haplotypes between *A. gossypii* and *A. rhamnicola* (and between other potential species showing strong differentiation with nuclear markers) could be the result of incomplete lineage sorting rather than evidence of ongoing hybridization. I cannot judge which explanation is more likely, and the authors have every right to discuss why they consider hybridization more likely based on the available evidence. But this is not done in the paper. There is some unconvincing rebuttal in the cover letter and the term incomplete lineage sorting does not even show up in the paper’s Discussion. This is not thorough.

>>

[Response and rebuttal]

We have reflected in the manuscript what the reviewer and editor pointed out. As pointed out by reviews comments, doing lineage sorting by *COI* haplotype results is likely unclear at the first submitted manuscript. Since *A. gossypii* and *A. rhamnicola* cross-sharing haplotypes in several host plants, there is a problem with lineage sorting or DNA identification with only *COI* haplotypes (barcoding). However, I am not saying clearly that our sample is misidentified with the two species, *A. gossypii* and *A. rhamnicola*. In particular, it was revealed that genotyping by microsatellite was the only way to distinguish between these two evolutionarily intermediate species, even for organizing the sampled individuals in HAP type and utilizing them for analysis.

Accepting these editor and reviewer’s opinions, the identification content by *COI* haplotype (barcoding) was deleted and supplemented with the concept of species lineage (group) sorting instead. Therefore, ‘Groups’ sorted by lineage in the table are specified and subdivided into *A. gossypii* Group1, *A. g.* Group 2, *A. rhamnicola* Group 1, *A. r.* Group 2, *A. r.* Group 3. In addition, according to our new lineage sorting, species names were deleted in Fig 2 and Fig 4. since specifying the species creates a rather confusing situation and misunderstanding of original purpose of study. The changes in the text are as follows.

"The two *Aphis species*, *A. gossypii* and *A. rhamnicola*, we study here are not only very similar in morphology, but also share several host plants due to the polyphagy. Although we performed species identification through morphology and host plant relationships as a first step and also tested DNA barcoding for all individuals collected on their shared host plants (eg *Capsella*, *Rhamnus*, and *Rubia*), we found that there were a lot of the haplotypes cross-shared between *A. gossypii* and *A. rhamnicola* (see Results). Therefore, instead of identifying the species with 36 HAPs, we applied the dominant assignment (white, green, blue, red, dark blue) of the genetic structure (K =3, 4, 5) by STRUCTURE as well as the PCoA results (see Results) to sort their lineags into five groups as *Aphis gossypii* Group 1, A. g. Group 2, *A. rhmanicola* Group 1, A. r. Group 2 and A. r. Group 3 (Table 1). *'Aphis gossypii*' and'A*. rhamincola*', which are mentioned later, are meant to include all group lin eages containing the HAPs assigned by the results. Table S3 shows detailed information for lineage sorted samples used in DNA analyses." **-> LINE 202-211, Table 1, Fig2, Fig4 revised**

In addition, the content of HAP mixing in the intermediating host plant based on *COI* or *16S* hybridization is sufficiently mentioned in the discussion by citing a paper by Lozier et al. [89]. Please check the contents at the end of the discussion. In relation to *COI* hybridization, hybridized population of *Hyalopterus pruni* has already found by Lozier et al. [89]. It has been explored and studied by and presented the second observation in aphids and corroborating results in our study. It is emphasized once again that the phenomenon of cross-sharing of *COI* haplotypes between the two species is by no means due to errors in lineage sorting.

[2]

Reviewer 1 continues to be concerned with over- or mis-interpretation of your population genetics data.  You should address the following points in your revision:

The conclusions you draw about the cause of heterozygote excess or heterozygote deficit (lines 455-464) are not appropriate for the Results section. If retained, this section should be moved to the Discussion, and the comments of Reviewer 1 addressed.

Editor: Similarly, reviewer 1 criticized the misleading interpretation of some standard population genetic indices (heterozygosities etc.). While the authors did check whether some of the deviations from HWE might be due to the inclusion of multiple clonal copies of the same genotypes within populations, the (over-)interpreted summary of the results remained completely unchanged in the paper. For example, this part:

“Heterozygote excess in Ag_CA, Ag_CP, and Ar_PE were likely the result of heterosis or over-dominance related to selection preference toward heterozygous combination [81], or fixation of heterozygous genotypes; and, correspondingly, negative FIS values also showed an increase in heterozygosity that was generally due to random mating or outbreeding [82]. In contrast, heterozygote deficit (i.e., homozygote excess) in Ag_CJ, Ar_YO, Ar_PH, and Ar_RU was likely caused by retaining numerous unique genotypes with private alleles within a population, and positive FIS values explained that the amount of heterozygous offspring in the population decreased, usually due to inbreeding [82].”

This is scientifically unsound in several respects. Without other, independent evidence it is simply not possible to infer heterosis as the cause of heterozygote excess at neutral markers.

Random mating will restore HWE and not lead to “an increase in heterozygosity”. Etc.

>>

[Response and rebuttal]

Because aphids ‘parthenogenetically’ reproduce in their secondary host, especially under anholocyclic life, the result of heterosis or over-dominance related to selection preference toward heterozygous combination or fixation of heterozygous genotypes have been commonly reported in study of aphids. This is far from the normal reproductive situation under random mating on other diploid organisms. We cited additional references. Please see citations below:

81.	Delmotte F, Sabater-Munoz B, Prunier-Leterme N, Latorre A, Sunnucks P, Rispe C, et al. Phylogenetic evidence for hybrid origins of asexual lineages in an aphid species. Evolution. 2003;57(6):1291-303. doi: Doi 10.1554/02-557. PubMed PMID: WOS:000183997400007.

82.	Simon JC, Baumann S, Sunnucks P, Hebert PDN, Pierre JS, Le Gallic JF, et al. Reproductive mode and population genetic structure of the cereal aphid Sitobion avenae studied using phenotypic and microsatellite markers. Molecular Ecology. 1999;8(4):531-45. doi: DOI 10.1046/j.1365-294x.1999.00583.x. PubMed PMID: WOS:000080177700003.

83.	Delmotte F, Leterme N, Gauthier JP, Rispe C, Simon JC. Genetic architecture of sexual and asexual populations of the aphid *Rhopalosiphum padi* based on allozyme and microsatellite markers. Molecular Ecology. 2002;11(4):711-23. doi: DOI 10.1046/j.1365-294X.2002.01478.x. PubMed PMID: WOS:000175250300007.

84.	Wilson ACC, Sunnucks P, Blackman RL, Hales DF. Microsatellite variation in cyclically parthenogenetic populations of *Myzus persicae* in south-eastern Australia. Heredity. 2002;88(4):258-66. doi: 10.1038/sj.hdy.6800037.

In the respects to the description of heterozygote deficit, in the contrary to parthenogenetic life, sexual lineages generally show the heterozygote deficits [81-84]. However, as you pointed out, I cannot connect it without confirming clear evidence of correlation between sexual lineages and ecology of HAPs sampled from our study, thus deleted it (explanation of heterozygote deficits. Finally, the sentences were amended as below:

“Heterozygote excess in Ag_CA, Ag_CP, and Ar_PE were likely the result of heterosis or over-dominance related to selection preference toward heterozygous combination or fixation of heterozygous genotypes due to parthenogenesis of aphids in secondary host, especially under anholocyclic (permanently asexual) life [81]. Similar to our results, this phenomenon was already reported from several aphid species such as *Sitobion avenae, Myzus persicae* and *Rhopalosiphum padi* having permanently or temporary asexual life, which showed the significant heterozygote excess [82-84]. Negative *FIS* values also showed an increase in heterozygosity that was generally due to random mating or outbreeding, whereas positive *FIS* values explained that the amount of heterozygous offspring in the population decreased, usually due to inbreeding [85].” **-> LINE 459-466 revised**

Unlike the reviewer's request, because we do not treat discussion of the heterozygote excess in Ag_CA, Ag_CP, and Ar_PE, we still remain these contents in the results section in view of consistency of manuscript content.

[3]

You might also consider:

Providing a citation and/or example species in support of the statement in lines 632-634.

Add the related references to support the statement:

“This is similar to the tendency found in most polyphagous aphids that the primary host is shared, but the secondary host is completely different [32, 86-89].”

32.	Moran NA. The Evolution of Aphid Life-Cycles. Annual Review of Entomology. 1992;37:321-48. doi: DOI 10.1146/annurev.ento.37.1.321. PubMed PMID: WOS:A1992GY50200014.

86.	Lozier JD, Roderick GK, Mills NJ. Genetic evidence from mitochondrial, nuclear, and endosymbiont markers for the evolution of host plant associated species in the aphid genus *Hyalopterus* (Hemiptera : Aphididae). Evolution. 2007;61(6):1353-67. doi: DOI 10.1111/j.1558-5646.2007.00110.x. PubMed PMID: WOS:000247125100009.

87.	Margaritopoulos JT, Tsitsipis JA, Goudoudaki S, Blackman RL. Life cycle variation of *Myzus persicae* (Hemiptera: Aphididae) in Greece. Bulletin of Entomological Research. 2002;92(4):309-19. Epub 2002/08/23. doi: 10.1079/BER2002167

S0007485302000366 [pii]. PubMed PMID: 12191439.

88.	Guldemond JA. Host Plant Relationships and Life-Cycles of the Aphid Genus *Cryptomyzus*. Entomologia Experimentalis Et Applicata. 1991;58(1):21-30. PubMed PMID: WOS:A1991EZ54000003.

89.	Jousselin E, Genson G, Coeur d'Acier A. Evolutionary lability of a complex life cycle in the aphid genus *Brachycaudus*. BMC evolutionary biology. 2010;10(1):295. doi: 10.1186/1471-2148-10-295. PubMed PMID: 20920188; PubMed Central PMCID: PMCPMC2958166.
